# Synthesis and biological evaluation of novel *N*^9^-heterobivalent β-carbolines as angiogenesis inhibitors

**DOI:** 10.1080/14756366.2018.1497619

**Published:** 2019-01-02

**Authors:** Liang Guo, Qin Ma, Wei Chen, Wenxi Fan, Jie Zhang, Bin Dai

**Affiliations:** a School of Chemistry and Chemical Engineering/Key Laboratory for Green Processing of Chemical Engineering of XinJiang Bingtuan, Shihezi University, Shihezi, China;; b XinJiang Huashidan Pharmaceutical Research Co. Ltd., Urumqi, China

**Keywords:** Heterobivalent β-carboline, cytotoxic activities, angiogenesis inhibitors, structure–activity relationship

## Abstract

A series of novel *N*^9^-heterobivalent β-carbolines has been synthesized. All the novel compounds were tested for their anticancer activity against six tumour cell lines *in vitro*. Among these molecules, compounds **5b**, and **5w** exhibited strong cytotoxic activities with IC_50_ value of lower than 20 μM. Acute toxicities and antitumor efficacies of the selected compounds in mice were also evaluated, compounds **5b** and **5w** exhibited that tumour inhibition rate of over 40% in the Sarcoma 180 and Lewis lung cancer animal models. Preliminary structure–activity relationships (SARs) analysis indicated that: (1) *C*^1^-methylation and *C*^7^-methoxylation were favorable for increased activities; (2) 3-Pyridyl or 2-thienyl group substituent into position-1 of the β-carboline core, and the aryl substituent into another β-carboline ring might be detrimental to cytotoxic effects of this class compounds. Investigation of the preliminary mechanism of action demonstrated that compound **5b** had obvious angiogenesis inhibitory effects in the chicken chorioallantoic membrane (CAM) assay.

## Introduction

The population growth and aging associated with some risk factors leveraged the incidence of new cases of cancer and related deaths in developed countries and in developing countries^1^. Cancer resistance to therapy is becoming a common phenomenon that threatens the current strategies adopted against this disease. For that reason we need to discover new anticancer agents. One of the successful and effective methods for the discovery of new anticancer drugs from natural products is to synthesize novel compounds through chemical structural modifications on the basis of leading compounds.

*Peganum harmala* L. have been traditionally used for hundreds of years to treat the alimentary tract cancers and malaria in Northwest China. Harmine, originally isolated from the seeds of *Peganum harmala* L. in 1847, is the most representative natural occurring β-carboline alkaloid, having a common tricyclic pyrido [3,4-*b*] indole ring structure. In the past several decades, it has confirmed that harmine was the important active ingredients to treat the alimentary tract cancers^2^^,^^3^. Recent reports demonstrated that harmine and its derivatives had remarkable antitumor activities, together with potential neurotoxicity^2–5^. Moreover, it has been reported that harmine and its derivatives can exert antitumor activities through multiple mechanisms, such as DNA binding^6–8^, inhibition topoisomerases I and II^9^^,^^10^, CDK (cyclin-dependent kinase)^11^^,^^12^, PLK1 (polo-like kinase)^13^, lipoxygenase^14^^,^^15^ and IκB kinases^16^.

**Figure 1.**
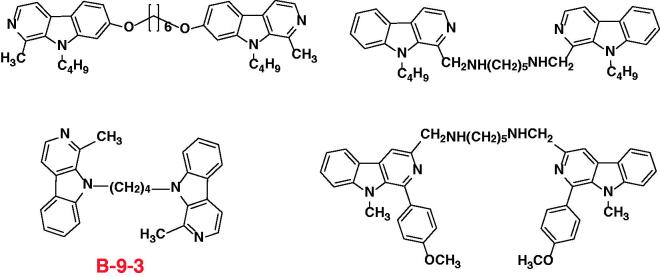
The chemical structure of the representative reported symmetric bivalent β-carbolines.

Previous investigations has shown that some dimer antitumor agents via an appropriate linker could lead to significantly improved antitumor activities (100- to 500-fold improvement over the corresponding monomers)^17–20^. Therefore, bivalent β-carbolines were expected to exhibit more potent antitumor efficacies than monomers. Inspired by this information, our group reported the synthesis, *in vitro* evaluation, *in vivo* efficacies and structure-activity relationships for the novel homobivalent β-carbolines with an alkyl spacer or alkylamino spacer in positon-1, 3, 7, and 9 of the β-carboline nucleus, respectively (Figure 1)^21–26^. In these compounds, 1-Methyl-9-[4–(1-methyl-β-carboline-9-yl)butyl]-β-carboline (B-9–3)^21^^,^^27^ exhibited potent antitumor activity. It was a symmetric dimeric β-carboline compound that contains two molecules of harman bound to each other by a tetramethylene group. The pharmacological mechanisms showed that the angiogenesis inhibitor B-9–3 selectively induces apoptosis of endothelial cells, in part through disruption of VEGF-A/VEGFR2 signaling^28^.

Our strategy was based on the modification of the prototype B-9–3, following this previous work, we have continued our search for novel antitumor agents endowed with better antitumor activities, and we provide detailed studies of structure–activity relationships (SARs) on the antitumor efficacies *in vitro* and *in vivo* of this class of compounds. Here, we designed and synthesized a series of novel *N*^9^-heterobivalent β-carboline derivatives as potent antitumor agents.

## Materials and methods

### Chemistry

All reagents were purchased from commercial suppliers and were dried and purified when necessary. The following intermediates **3a**-**3b**^29^, **3c**-**3e**^21^, **3f**^30^, **3g**^31^, **3h**^32^, **4g**-**4h**^33^ were prepared as previously described.

Melting points were determined in capillary tubes on an electrothermal X-5 apparatus and without correction. NMR spectra were recorded at room temperature on a Bruker Avance III HD 400 instrument at 400 MHz for ^1^H NMR and 100 MHz for ^13^C NMR. Chemical shifts (*δ*) are reported in ppm relatively to the residual solvent peak and the multiplicity of each signal is designated by the following abbreviations: s, singlet; d, doublet; t, triplet; q, quartet; m, multiplet. Coupling constants (*J*) are quoted in Hz. High resolution mass spectra (HRMS) were recorded on Bruker ultrafleXtreme MALDI-TOF/TOF-MS and HCCA (alpha-cyano-4-hydroxycinnamic acid) is used as matrix. Elemental analyses (C, H, and N) were carried out on an Elementar Vario ELIII CHNS Elemental Analyzer. Column chromatography was performed with silica gel (200–300 mesh) and analytical TLC on silica gel 60-F_254_.

### General procedure for the preparation of compounds 4a-h

A mixture of **3a** (1.68 g, 10 mmol) and anhydrous DMF (50 ml) was stirred at room temperature for 0.5 h, then NaH (0.50 g, 20 mmol) and the 1,4-dibromobutane (20 mmol) were added. The mixture was stirred at room temperature. After that period, the mixture was monitored via TLC and at the end of the reaction the mixture was poured into H_2_O (150 ml) and extracted with ethyl acetate. The organic phase was collected and washed with water and brine, then dried under anhydrous sodium sulfate, filtered, and evaporated. The resulting oil was crystallized from ethyl ether or ethyl ether-petroleum ether to afford the compound **4a**. Products **4b**-**h** were prepared according to the same method of **4a**.

#### 9–(4-bromobutyl)-β-carboline (4a)

Colorless crystals, yield 87%, m.p. 285.3–286.8 °C. ^1^H NMR (400 MHz, DMSO-*d_6_*): *δ* 9.10 (s, 1H), 8.40 (d, *J* = 5.2 Hz, 1H), 8.28 (d, *J* = 8.0 Hz, 1H), 8.14 (dd, *J* = 5.2, 0.8 Hz, 1H), 7.75 (d, *J* = 8.4 Hz, 1H), 7.60–7.64 (m, 1H), 7.27–7.31 (m, 1H), 4.56 (t, *J* = 6.8 Hz, 2H), 3.56 (t, *J* = 6.4 Hz, 2H), 1.90–1.97 (m, 2H), 1.81–1.88 (m, 2H). ^13^C NMR (100 MHz, DMSO-*d*_6_): *δ* 141.13, 138.88, 136.47, 133.13, 128.77, 127.66, 122.44, 120.80, 119.92, 115.04, 110.62, 42.15, 35.09, 30.23, 27.87.

#### 9–(4-bromobutyl)-1-methyl-β-carboline (4b)

Colorless crystals, yield 88%, m.p. 213.9–215.6 °C. ^1^H NMR (400 MHz, DMSO-*d_6_*): *δ* 8.71 (dd, *J* = 6.4, 1.2 Hz, 1H), 8.54 (dd, *J* = 8.0, 1.2 Hz, 1H), 8.49 (dd, *J* = 6.4, 2.0 Hz, 1H), 8.00 (d, *J* = 8.4 Hz, 1H), 7.83–7.87 (m, 1H), 7.46–7.50 (m, 1H), 4.75 (t, *J* = 7.6 Hz, 2H), 3.70 (t, *J* = 6.4 Hz, 2H), 3.27 (s, 3H), 2.04–1.77 (m, 4H). ^13^C NMR (100 MHz, DMSO-*d_6_*): *δ* 144.33, 139.00, 133.74, 133.26, 132.07, 129.29, 123.86, 122.14, 119.67, 116.15, 111.78, 45.35, 44.31, 29.63, 28.29, 18.31.

#### 9–(5-bromopentyl)-1-methyl-β-carboline (4c)

Colorless crystals, yield 90%, m.p. 187.7–189.5 °C. ^1^H NMR (400 MHz, CDCl_3_): *δ* 8.42–8.33 (m, 2H), 8.29 (dt, *J* = 8.0, 1.2 Hz, 1H), 7.82–7.86 (m, 1H), 7.66 (dt, *J* = 8.4, 0.8 Hz, 1H), 7.48–7.51 (m, 1H), 4.69 (t, *J* = 7.6 Hz, 2H), 3.52 (s, 3H), 1.82–1.98 (m, 4H), 1.60–1.68 (m, 2H). ^13^C NMR (100 MHz, CDCl_3_): *δ* 144.30, 137.12, 133.87, 133.76, 132.22, 128.57, 123.11, 122.38, 119.70, 115.13, 110.68, 45.35, 44.43, 31.86, 30.29, 24.04, 18.07.

#### 9–(6-bromohexyl)-1-methyl-β-carboline (4d)

Colorless crystals, yield 69%, m.p. 73.9–74.8 °C. ^1^H NMR (400 MHz, CDCl_3_): *δ* 8.33 (d, *J* = 5.2 Hz, 1H), 8.12 (d, *J* = 7.6 Hz, 1H), 7.84 (d, *J* = 5.2 Hz, 1H), 7.56–7.60 (m, 1H), 7.45 (d, *J* = 8.4 Hz, 1H), 7.25–7.29 (m, 1H), 4.54 (t, *J* = 7.6 Hz, 2H), 3.38 (t, *J* = 6.4 Hz, 2H), 3.05 (s, 3H), 1.81–1.89 (m, 4H), 1.40–1.55 (m, 4H). ^13^C NMR (100 MHz, CDCl_3_): *δ* 141.51, 141.13, 137.94, 135.09, 129.14, 128.17, 121.55, 121.33, 119.66, 112.99, 109.67, 44.76, 33.56, 30.70, 27.90, 26.13, 23.55.

#### 9–(4-bromobutyl)-1–(3-pyridyl)-β-carboline (4e)

Yellow crystals, yield 71%, m.p. 135.0–136.7 °C. ^1^H NMR (400 MHz, CDCl_3_): *δ* 8.67 (d, *J* = 6.0 Hz, 1H), 8.53 (d, *J* = 6.0 Hz, 1H), 8.37 (d, *J* = 8.0 Hz, 1H), 7.87–7.81 (m, 2H), 7.68–7.64 (m, 2H), 7.54–7.49 (m, 1H), 7.36–7.33 (m, 1H), 4.19 (t, *J* = 8.0 Hz, 2H), 3.32 (dt, *J* = 55.2, 6.4 Hz, 2H), 1.77–1.69 (m, 2H), 1.60–1.44 (m, 2H). ^13^C NMR (100 MHz, CDCl_3_): *δ* 149.60, 149.24, 142.21, 139.90, 138.50, 137.27, 131.21, 129.04, 128.23, 125.31, 123.53, 121.85, 121.25, 120.45, 114.54, 110.17, 43.77, 32.59, 29.44, 27.35.

#### 9–(4-bromobutyl)-1–(2-thienyl)-β-carboline (4f)

Light yellow crystals, yield 60%, m.p. 197.6–199.8 °C. ^1^H NMR (400 MHz, CDCl_3_): *δ* 8.55 (d, *J* = 5.2 Hz, 1H), 8.19 (d, *J* = 7.6 Hz, 1H), 8.03 (d, *J* = 5.2 Hz, 1H), 7.62–7.54 (m, 3H), 7.54–7.51 (m, 1H), 7.49–7.41 (m, 3H), 7.30 (t, *J* = 7.2 Hz, 1H), 4.03–3.78 (m, 2H), 3.20 (dt, *J* = 54.8, 6.4 Hz, 2H), 1.71–1.49 (m, 2H), 1.44–1.24 (m, 2H). ^13^C NMR (100 MHz, CDCl_3_): *δ* 141.58, 140.89, 138.69, 138.31, 134.19, 131.47, 130.09, 129.47, 128.58, 126.95, 121.70, 121.26, 119.96, 114.31, 109.76, 43.23, 32.60, 29.77, 27.94.

### General procedure for the preparation of compounds 5a-x

A solution of compound **3c** (2 mmol) in anhydrous DMF (6 ml) was added slowly with stirring to a solution of **4a** (3 mmol), NaH (0.25 g, 10 mmol), potassium iodide (1.68 g, 10 mmol) in anhydrous DMF (25 ml). The mixture was stirred at room temperature until the reaction is completed. Then the mixture was poured into ice-cold water. The reaction mixture was extracted with ethyl acetate (3 × 30 ml), washed with water and brine, dried over anhydrous Na_2_SO_4_, and filtered. Purification by column chromatography (DCM/MeOH 100:1 as the eluent) furnished the dimeric β-carboline **5a**. Compounds **5b-x** were synthesized using similar procedure as compound **5a**.

#### 1–(3-pyridyl)-9–(4-(β-carboline-9-yl)butyl)-β-carboline (5a)

This compound was obtained as colourless crystals in 65% yield, m.p. 173.3–175.1 °C. ^1^H NMR (400 MHz, CDCl_3_) *δ*: 8.77 (dd, *J* = 2.4, 0.8 Hz, 1H), 8.65 (d, *J* = 0.8 Hz, 1H), 8.61 (dd, *J* = 4.8, 1.6 Hz, 1H), 8.50 (d, *J =* 5.2 Hz, 1H), 8.47 (d, *J =* 5.2 Hz, 1H), 8.15–8.18 (m, 2H), 8.00 (dd, *J =* 5.2, 0.8 Hz, 1H), 7.98 (d, *J =* 5.2 Hz, 1H), 7.56–7.63 (m, 3H), 7.30–7.34 (m, 3H), 7.24 (d, *J =* 8.4 Hz, 1H), 7.11–7.15 (m, 1H), 4.14 (t, *J* = 7.2 Hz, 2H), 3.96 (t, *J* = 7.2 Hz, 2H), 1.38–1.41 (m, 4H). ^13^C NMR (100 MHz, CDCl_3_) *δ:* 150.00, 149.59, 142.09, 141.14, 140.25, 138.92, 138.05, 136.35, 136.06, 135.77, 134.09, 130.89, 128.85, 128.80, 123.04, 122.11, 121.81, 121.26, 120.90, 120.33, 120.02, 114.80, 114.22, 109.90, 109.37, 44.08, 42.65, 26.53, 26.01. HRMS calcd for C_31_H_26_N_5_ [M + H]^+^ 468.2183, found 468.2183. Anal. calcd for C_31_H_25_N_5_: C, 79.63; H, 5.39; N, 14.98; found C 79.16, H 5.49, N 14.38.

#### 1–(3-pyridyl)-9–(4-(1-methyl-β-carboline-9-yl)butyl)-β-carboline (5b)

This compound was obtained as slight yellow crystals in 54% yield, m.p. 201.2–202.2 °C. ^1^H NMR (400 MHz, CDCl_3_) *δ:* 8.79 (dd, *J =* 2.4, 0.8 Hz, 1H), 8.65 (dd, *J =* 4.8, 1.6 Hz, 1H), 8.52 (d, *J =* 5.2 Hz, 1H), 8.33 (d, *J =* 5.2 Hz, 1H), 8.17–8.19 (m, 1H), 8.11 (d, *J =* 7.6 Hz, 1H), 7.98 (d, *J =* 5.2 Hz, 1H), 7.84 (d, *J =* 5.2 Hz, 1H), 7.69–7.71 (m, 1H), 7.52–7.61 (m, 2H), 7.27–7.35 (m, 3H), 7.18–7.24 (m, 2H), 4.23 (t, *J =* 7.2 Hz, 2H), 3.96 (t, *J =* 7.2 Hz, 2H), 2.81 (s, 3H), 1.37–1.45 (m, 2H), 1.28–1.35 (m, 2H). ^13^C NMR (100 MHz, CDCl_3_) *δ*_:_ 150.06, 149.64, 142.18, 141.40, 140.66, 140.31, 139.05, 136.41, 135.83, 134.70, 134.12, 130.97, 129.35, 128.86, 128.39, 123.11, 121.85, 121.63, 121.34, 121.17, 120.38, 119.91, 114.23, 113.07, 109.95, 109.55, 44.13, 43.90, 29.70, 27.71, 26.07. HRMS calcd for C_32_H_28_N_5_ [M + H]^+^ 482.2339, found 482.2342. Anal. calcd for C_32_H_27_N_5_: C, 79.81; H, 5.65; N, 14.54; found C 79.39, H 5.37, N 14.75.

#### 1–(3-pyridyl)-9–(5-(1-methyl-β-carboline-9-yl)pentyl)-β-carboline (5c)

This compound was obtained as yellow crystals in 62% yield, m.p. 189.4–190.5 °C. ^1^H NMR (400 MHz, CDCl_3_) *δ*: 8.82 (d, *J =* 2.0 Hz, 1H), 8.55 (d, *J =* 5.2 Hz, 1H), 8.51 (dd, *J* = 4.8, 1.6 Hz, 1H), 8.34 (d, *J* = 5.2 Hz, 1H), 8.19 (d, *J =* 7.6 Hz, 1H), 8.14 (d, *J =* 8.4 Hz, 1H), 8.02 (d, *J* = 5.2 Hz, 1H), 7.85–7.89 (m, 2H), 7.57–7.61 (m, 2H), 7.37 (d, *J =* 8.4 Hz, 1H), 7.29–7.33 (m, 3H), 7.23–7.25 (m, 1H), 4.35 (t, *J =* 7.6 Hz, 2H), 3.95 (t, *J =* 7.6 Hz, 2H), 2.96 (s, 3H), 1.47–1.55 (m, 2H), 1.35–1.42 (m, 2H), 0.86–0.94 (m, 2H). ^13^C NMR (100 MHz, CDCl_3_) *δ*: 150.12, 149.51, 142.03, 141.61, 140.69, 140.38, 138.84, 137.29, 136.56, 135.99, 134.87, 134.27, 130.85, 129.51, 128.80, 128.52, 123.01, 121.76, 121.67, 121.30, 121.16, 120.27, 119.98, 114.26, 113.15, 110.01, 109.69, 44.48, 44.24, 30.29, 28.56, 23.87, 23.07. HRMS calcd for C_33_H_30_N_5_ [M + H]^+^ 496.2496, found 496.2503. Anal. calcd for C_33_H_29_N_5_: C, 79.97; H, 5.90; N, 14.13; found C 79.11, H 5.88, N 13.90.

#### 1–(3-pyridyl)-9–(6-(1-methyl-β-carboline-9-yl)hexyl)-β-carboline (5d)

This compound was obtained as yellow crystals in 66% yield, m.p. 141.3–142.9 °C. ^1^H NMR (400 MHz, CDCl_3_) *δ*: 8.89 (dd, *J* = 2.0, 0.8 Hz, 1H), 8.72 (dd, *J* = 4.8, 1.6 Hz, 1H), 8.56 (d, *J* = 5.2 Hz, 1H), 8.31 (d, *J* = 5.2 Hz, 1H), 8.17–8.19 (m, 1H), 8.08–8.10 (m, 1H), 8.02 (d, *J* = 5.2 Hz, 1H), 7.93–7.96 (m, 1H), 7.82 (d, *J* = 5.2 Hz, 1H), 7.53–7.60 (m, 2H), 7.30–7.41 (m, 4H), 7.24–7.28 (m, 1H), 4.39 (t, *J* = 7.2 Hz, 2H), 3.97 (t, *J* = 7.2 Hz, 2H), 2.97 (s, 3H), 1.58–1.66 (m, 2H), 1.29–1.37 (m, 2H), 1.07–1.15 (m, 2H), 0.81–0.90 (m, 2H). ^13^C NMR (100 MHz, CDCl_3_) *δ*: 150.16, 149.54, 142.11, 141.45, 141.02, 140.44, 138.79, 137.84, 136.65, 136.07, 134.98, 134.35, 130.86, 129.13, 128.73, 128.15, 123.07, 121.72, 121.53, 121.31, 121.26, 120.19, 119.66, 114.23, 112.98, 110.10, 109.60, 44.54, 44.39, 30.59, 28.66, 26.33, 23.44. HRMS calcd for C_34_H_32_N_5_ [M + H]^+^ 510.2652, found 510.2656. Anal. calcd for C_34_H_31_N_5_: C, 80.13; H, 6.13; N, 13.74; found C 79.72, H 6.14, N 13.32.

#### 1–(2-thienyl)-9–(4-(β-carboline-9-yl)butyl)-β-carboline (5e)

This compound was obtained as yellow crystals in 56% yield, m.p. 141.6–143.1 °C. ^1^H NMR (400 MHz, CDCl_3_) *δ*: 8.72 (d, *J =* 0.8 Hz, 1H), 8.49 (d, *J* = 5.2 Hz, 1H), 8.46 (d, *J* = 5.2 Hz, 1H), 8.15–8.16 (m, 1H), 8.13–8.14 (m, 1H), 7.97 (dd, *J* = 5.2, 0.8 Hz, 1H), 7.95 (d, *J* = 5.2 Hz, 1H), 7.51–7.71 (m, 3H), 7.33–7.35 (m, 1H), 7.30–7.32 (m, 1H), 7.28–7.29 (m, 1H), 7.24–7.28 (m, 2H), 7.05 (dd, *J* = 4.0, 1.2 Hz, 1H), 6.94 (dd, *J* = 5.2, 3.6 Hz, 1H), 4.14 (t, *J* = 7.2 Hz, 2H), 4.13 (t, *J* = 7.2 Hz, 2H), 1.47–1.58 (m, 4H). ^13^C NMR (100 MHz, CDCl_3_) *δ*: 141.95, 141.05, 140.84, 138.62, 136.84, 136.22, 134.60, 132.44, 131.55, 130.86, 130.80, 128.78, 128.68, 128.54, 128.49, 128.21, 127.03, 126.58, 122.04, 121.73, 121.28, 121.02, 120.17, 119.79, 114.70, 114.23, 109.90, 109.34, 43.91, 42.68, 26.98, 26.28. HRMS calcd for C_30_H_25_N_4_S [M + H]^+^ 473.1794, found 473.1792. Anal. calcd for C_30_H_24_N_4_S: C, 76.24; H, 5.12; N, 11.86; S, 6.78; found C, 76.01; H, 5.61; N, 11.46; S, 6.34.

#### 1–(2-thienyl)-9–(4-(1-methyl-β-carboline-9-yl)butyl)-β-carboline (5f)

This compound was obtained as yellow crystals in 47% yield, m.p. 214.8–216.0 °C. ^1^H NMR (400 MHz, CDCl_3_) *δ*: 8.50 (d, *J* = 5.2 Hz, 1H), 8.32 (d, *J* = 5.2 Hz, 1H), 8.14–8.17 (m, 1H), 8.12–8.06 (m, 1H), 7.96 (d, *J* = 5.2 Hz, 1H), 7.83 (d, *J* = 5.2 Hz, 1H), 7.56–7.60 (m, 1H), 7.51–7.55 (m, 1H), 7.35–7.37 (m, 1H), 7.33–7.34 (m, 1H), 7.28–7.31(m, 1H), 7.24–7.26 (m, 1H), 7.20–7.23 (m, 1H), 7.12 (dd, *J =* 4.0, 1.2 Hz, 1H), 6.99 (dd, *J =* 5.2, 3.6 Hz, 1H), 4.26 (t, *J* = 7.2 Hz, 2H), 4.13 (t, *J* = 7.2 Hz, 2H), 2.89 (s, 3H), 1.41–1.55 (m, 4H). ^13^C NMR (100 MHz, CDCl_3_) *δ*: 142.02, 141.36, 140.95, 140.86, 138.72, 137.86, 136.90, 134.85, 134.59, 130.89, 129.24, 128.70, 128.22, 127.07, 126.67, 121.76, 121.60, 121.36, 121.25, 120.22, 119.79, 114.23, 113.03, 109.94, 109.55, 44.04, 43.94, 27.94, 26.52, 23.35. HRMS calcd for C_31_H_27_N_4_S [M + H]^+^ 487.1951, found 487.1950. Anal. calcd for C_31_H_26_N_4_S: C, 76.51; H, 5.39; N, 11.51; S, 6.59; found C, 76.27; H, 5.59; N, 10.93; S, 6.38.

#### 1–(2-thienyl)-9–(5-(1-methyl-β-carboline-9-yl)pentyl)-β-carboline (5g)

This compound was obtained as yellow crystals in 62% yield, m.p. 158.4–159.2 °C. ^1^H NMR (400 MHz, CDCl_3_) *δ*: 8.51 (d, *J* = 5.2 Hz, 1H), 8.34 (d, *J* = 5.2 Hz, 1H), 8.15–8.17 (m, 1H), 8.11–8.14 (m, 1H), 7.97 (d, *J* = 5.2 Hz, 1H), 7.86 (d, *J* = 5.2 Hz, 1H), 7.54–7.60 (m, 2H), 7.40 (d, *J =* 8.4 Hz, 1H), 7.27–7.32 (m, 3H), 7.21 (dd, *J =* 5.2, 1.2 Hz, 1H), 7.16 (dd, *J =* 3.2, 1.2 Hz, 1H), 6.91 (dd, *J =* 5.2, 3.6 Hz, 1H), 4.37 (t, *J =* 7.6 Hz, 2H), 4.12 (t, *J* = 7.6 Hz, 2H), 2.96 (s, 3H), 1.53–1.60 (m, 2H), 1.42–1.50 (m, 2H), 0.98–1.06 (m, 2H). ^13^C NMR (100 MHz, CDCl_3_) *δ*: 142.08, 141.45, 141.00, 140.94, 138.55, 137.76, 136.96, 134.97, 134.66, 130.82, 129.21, 128.62, 128.29, 128.23, 126.85, 126.51, 121.67, 121.55, 121.27, 121.25, 120.09, 119.75, 114.16, 113.02, 110.02, 109.66, 44.54, 44.06, 30.22, 28.89, 23.98, 23.40 . HRMS calcd for C_32_H_29_N_4_S [M + H]^+^ 501.2107, found 501.2113. Anal. calcd for C_32_H_28_N_4_S: C, 76.71; H, 6.24; N, 10.84; S, 6.20; found C, 76.24; H, 5.92; N, 10.45; S, 6.07.

#### 1–(2-thienyl)-9–(6-(1-methyl-β-carboline-9-yl)hexyl)-β-carboline (5h)

This compound was obtained as yellow crystals in 57% yield, m.p. 238.5–239.6 °C. ^1^H NMR (400 MHz, CDCl_3_) *δ*: 8.52 (d, *J* = 5.2 Hz, 1H), 8.32 (d, *J* = 5.2 Hz, 1H), 8.15–8.17 (m, 1H), 8.09–8.11 (m, 1H), 7.97 (d, *J* = 5.2 Hz, 1H), 7.83 (d, *J* = 5.2 Hz, 1H), 7.53–7.59 (m, 2H), 7.45 (dd, *J =* 5.2, 1.2 Hz, 1H), 7.40 (d, *J* = 8.4 Hz, 1H), 7.35 (d, *J* = 8.4 Hz, 1H), 7.24–7.30 (m, 3H), 7.13 (dd, *J* = 5.2, 3.6 Hz, 1H), 4.39 (t, *J* = 7.6 Hz, 2H), 4.14 (t, *J* = 7.6 Hz, 2H), 2.98 (s, 3H), 1.61–1.68 (m, 2H), 1.38–1.46 (m, 2H), 1.13–1.21 (m, 2H), 0.91–0.98 (m, 2H). ^13^C NMR (100 MHz, CDCl_3_) *δ*: 142.18, 141.47, 141.21, 141.01, 138.54, 137.75, 137.09, 134.98, 134.77, 130.86, 129.14, 128.57, 128.27, 128.16, 126.98, 126.65, 121.66, 121.53, 121.34, 121.23, 120.06, 119.66, 114.12, 112.99, 110.20, 109.62, 44.58, 44.17, 30.61, 28.96, 26.43, 26.34, 23.40 . HRMS calcd for C_33_H_31_N_4_S [M + H]^+^ 515.2264, found 515.2268. Anal. calcd for C_33_H_30_N_4_S: C, 77.01; H, 5.88; N, 10.89; S, 6.23; found C, 76.80; H, 5.76; N, 10.72; S, 5.97.

#### 1–(2-chlorophenyl)-9–(4-(1-methyl-β-carboline-9-yl)butyl)-β-carboline (5i)

This compound was obtained as yellow crystals in 51% yield, m.p. 232.0–232.8 °C. ^1^H NMR (400 MHz, CDCl_3_) *δ*: 8.52 (d, *J* = 5.2 Hz, 1H), 8.34 (d, *J* = 5.2 Hz, 1H), 8.21–8.16 (m, 1H), 8.12–8.09 (m, 1H), 8.01 (d, *J* = 5.2 Hz, 1H), 7.85 (d, *J* = 5.2 Hz, 1H), 7.60–7.51 (m, 2H), 7.35–7.31 (m, 4H), 7.30–7.26 (m, 2H), 7.24–7.23 (m, 1H), 7.17–7.13 (m, 1H), 4.34–4.22 (m, 2H), 3.94–3.78 (m, 2H), 2.90 (s, 3H), 1.63–1.34 (m, 4H). ^13^C NMR (100 MHz, CDCl_3_) *δ*: 141.57, 141.41, 140.87, 140.83, 138.62, 138.44, 137.97, 134.89, 134.02, 133.93, 131.34, 130.13, 130.05, 129.45, 129.30, 128.63, 128.27, 126.72, 121.81, 121.64, 121.31, 120.05, 119.86, 114.34, 113.08, 109.62, 109.58, 44.06, 43.78, 28.23, 26.73, 23.45. HRMS calcd for C_33_H_28_ClN_4_ [M + H]^+^ 515.1997, found 515.2003. Anal. calcd for C_33_H_27_ClN_4_: C, 76.96; H, 5.28; N, 10.88; found C 76.39, H 5.37, N 10.57.

#### 1–(2-chlorophenyl)-9–(5-(1-methyl-β-carboline-9-yl)pentyl)-β-carboline (5j)

This compound was obtained as yellow crystals in 70% yield, m.p. 169.1–171.2 °C. ^1^H NMR (400 MHz, CDCl_3_) *δ*: 8.52 (d, *J* = 5.2 Hz, 1H), 8.36 (d, *J* = 5.2 Hz, 1H), 8.17–8.19 (m, 1H), 8.13–8.15 (m, 1H), 8.01 (d, *J* = 5.2 Hz, 1H), 7.87 (d, *J* = 5.2 Hz, 1H), 7.55–7.59 (m, 2H), 7.41 (dd, *J =* 7.6, 1.6 Hz, 1H), 7.36 (d, *J =* 8.4 Hz, 1H), 7.30–7.33 (m, 3H), 7.24–7.28 (m, 1H), 7.09–7.13 (m, 1H), 6.98–7.02 (m, 1H), 4.37 (t, *J =* 7.6 Hz, 2H), 3.76–3.93 (m, 2H), 2.97 (s, 3H), 1.35–1.56 (m, 4H), 0.88–1.02 (m, 2H). ^13^C NMR (100 MHz, CDCl_3_) *δ*: 141.58, 141.41, 140.97, 140.86, 138.72, 138.27, 137.92, 134.99, 134.09, 133.94, 131.43, 130.06, 129.85, 129.30, 129.17, 128.54, 128.23, 126.56, 121.71, 121.59, 121.29, 121.22, 119.91, 119.82, 114.30, 113.04, 109.68, 109.65, 44.52, 43.80, 30.20, 28.98, 24.03, 23.50. HRMS calcd for C_34_H_30_ClN_4_ [M + H]^+^ 529.2154, found 529.2161. Anal. calcd for C_34_H_29_ClN_4_: C, 77.19; H, 5.53; N, 10.59; found C 76.74, H 5.32, N 10.82.

#### 1–(4-methoxyphenyl)-9–(4-(1–(3-pyridyl)-β-carboline-9-yl)butyl)-β-carboline (5k)

This compound was obtained as yellow solid in 57% yield, m.p. 241.2–242.4 °C. ^1^H NMR (400 MHz, CDCl_3_) *δ*: 8.67 (d, *J* = 2.0 Hz, 1H), 8.62 (dd, *J* = 4.8, 1.6 Hz, 1H), 8.51 (d, *J* = 5.2 Hz, 1H), 8.47 (d, *J* = 5.2 Hz, 1H), 8.17 (m, 2H), 7.97 (d, *J* = 5.2 Hz, 1H), 7.91 (d, *J* = 5.2 Hz, 1H), 7.54–7.59 (m, 3H), 7.29–7.35 (m, 2H), 7.13–7.23 (m, 5H), 6.87 (s, 1H), 6.84 (s, 1H), 3.78 (s, 3H), 3.70–3.75 (m, 2H), 3.61–3.67 (m, 2H), 0.78–0.85 (m, 4H). ^13^C NMR (100 MHz, CDCl_3_) *δ*: 159.68, 149.96, 149.45, 143.69, 142.07, 141.98, 140.22, 138.83, 138.42, 136.39, 135.75, 133.95, 133.70, 132.04, 130.74, 130.44, 130.40, 128.73, 128.40, 122.96, 121.75, 121.65, 121.34, 121.19, 120.20, 119.95, 114.16, 113.43, 113.32, 110.01, 109.95, 55.34, 43.73, 43.41, 25.96, 25.80. HRMS calcd for C_38_H_32_N_5_O [M + H]^+^ 574.2601, found 574.2606. Anal. calcd for C_38_H_31_N_5_O: C, 79.56; H, 5.45; N, 12.21; found C 79.51, H 5.66, N 11.72.

#### 1–(3,4-dimethoxyphenyl)-9–(4-(1–(3-pyridyl)-β-carboline-9-yl)butyl)-β-carboline (5l)

This compound was obtained as yellow crystals in 63% yield, m.p. 239.8–240.5 °C. ^1^H NMR (400 MHz, CDCl_3_) *δ*: 8.68 (d, *J* = 2.0 Hz, 1H), 8.61 (dd, *J =* 4.8, 1.6 Hz, 1H), 8.51 (d, *J* = 5.2 Hz, 1H),8.48 (d, *J* = 5.2 Hz, 1H), 8.18 (d, *J* = 7.6 Hz, 1H), 8.16 (d, *J* = 7.6 Hz, 1H),7.97 (d, *J* = 5.2 Hz, 1H),7.95 (d, *J* = 5.2 Hz, 1H), 7.55–7.60 (m, 3H), 7.31–7.35 (m, 2H), 7.13–7.20 (m, 3H), 6.96 (d, *J* = 2.0 Hz, 1H), 6.85 (d, *J* = 8.0 Hz, 1H), 6.79 (d, *J* = 8.0 Hz, 1H), 3.84 (s, 3H), 3.80 (s, 3H), 3.76 (t, *J* = 6.4 Hz, 2H), 3.66 (t, *J* = 6.4 Hz, 2H), 0.82–0.92 (m, 4H). ^13^C NMR (100 MHz, CDCl_3_) *δ*: 149.95, 149.44, 149.20, 148.54, 143.62, 142.00, 141.87, 140.20, 138.83, 138.28, 136.36, 135.75, 133.98, 133.77, 132.17, 130.74, 130.46, 128.79, 128.49, 122.97, 121.73, 121.68, 121.34, 121.19, 120.23, 120.04, 114.19, 113.52, 112.45, 110.61, 110.09, 109.89, 55.99, 55.96, 43.82, 43.46, 26.05, 25.89. HRMS calcd for C_39_H_34_N_5_O_2_ [M + H]^+^ 604.2707, found 604.2705. Anal. calcd for C_39_H_33_N_5_O_2_: C, 77.59; H, 5.51; N, 11.60; found C 77.36, H 5.57, N 11.51.

#### 1–(3,4,5-trimethoxyphenyl)-9–(4-(1–(3-pyridyl)-β-carboline-9-yl)butyl)-β-carboline (5m)

This compound was obtained as yellow crystals in 49% yield, m.p. 231.0–232.3 °C. ^1^H NMR (400 MHz, CDCl_3_) *δ*: 8.71 (dd, *J* = 2.0, 0.8 Hz, 1H), 8.60 (dd, *J =* 4.8, 1.6 Hz, 1H), 8.51 (d, *J =* 0.8 Hz, 1H), 8.49 (d, *J =* 0.8 Hz, 1H), 8.18 (d, *J* = 8.0 Hz, 1H), 8.14 (d, *J* = 8.0 Hz, 1H), 7.98 (d, *J* = 5.2 Hz, 1H), 7.96 (d, *J* = 5.2 Hz, 1H), 7.57–7.62 (m, 3H), 7.30–7.36 (m, 2H), 7.21 (d, *J* = 8.4 Hz, 1H), 7.17 (d, *J* = 8.4 Hz, 1H), 7.11–7.15 (m, 1H), 6.67 (s, 2H), 3.80 (s, 3H), 3.78 (s, 6H), 3.76 (t, *J* = 6.4 Hz, 2H), 3.68 (t, *J* = 7.2 Hz, 2H), 0.95–0.98 (m, 4H). ^13^C NMR (100 MHz, CDCl_3_) *δ*: 153.03, 149.94, 149.43, 143.58, 141.94, 141.68, 140.15, 138.78, 138.28, 138.24, 136.34, 135.79, 135.24, 133.98, 133.74, 130.72, 130.39, 128.97, 128.57, 122.98, 121.69, 121.67, 121.31, 121.11, 120.28, 120.11, 114.20, 113.79, 110.13, 109.86, 106.54, 60.93, 56.24, 43.91, 43.54, 26.09, 25.94. HRMS calcd for C_40_H_36_N_5_O_3_ [M + H]^+^ 634.2813, found 634.2817. Anal. calcd for C_40_H_35_N_5_O_3_: C, 75.81; H, 5.57; N, 11.05; found C 75.79, H 5.56, N 11.32.

#### 1–(2-chlorophenyl)-9–(4-(1–(3-pyridyl)-β-carboline-9-yl)butyl)-β-carboline (5n)

This compound was obtained as yellow crystals in 58% yield, m.p. 177.6–178.9 °C. ^1^H NMR (400 MHz, CDCl_3_) *δ*: 8.73 (dd, *J* = 2.4, 0.8 Hz, 1H), 8.66 (dd, *J* = 4.8, 1.6 Hz, 1H), 8.53 (d, *J* = 5.2 Hz, 1H), 8.50 (d, *J* = 5.2 Hz, 1H), 8.17–8.21 (m, 2H), 8.00 (d, *J =* 2.4 Hz, 1H), 7.99 (d, *J =* 2.4 Hz, 1H), 7.64 (d, *J* = 7.6 Hz, 1H), 7.60–7.63 (m, 1H), 7.57–7.59 (m, 1H), 7.26–7.37 (m, 4H), 7.22–7.25 (m, 2H), 7.13–7.20 (m, 3H), 3.72 (t, *J =* 7.2 Hz, 2H), 3.48–3.64 (m, 2H), 0.84–0.93 (m, 4H). ^13^C NMR (100 MHz, CDCl_3_) *δ*: 149.99, 149.55, 142.03, 141.51, 140.52, 140.19, 138.80, 138.34, 138.14, 136.48, 135.71, 134.02, 134.00, 133.71, 131.32, 130.86, 130.04, 129.92, 129.23, 128.76, 128.62, 126.60, 123.02, 121.81, 121.72, 121.24, 121.09, 120.31, 120.02, 114.31, 114.24, 109.97, 109.71, 43.72, 43.19, 26.30, 26.08. HRMS calcd for C_37_H_29_ClN_5_ [M + H]^+^ 578.2106, found 578.2111. Anal. calcd for C_37_H_28_ClN_5_: C, 76.87; H, 4.88; N, 12.11; found C 76.38, H 4.85, N 11.75.

#### 1–(4-methoxyphenyl)-9–(4-(1–(2-thienyl)-β-carboline-9-yl)butyl)-β-carboline (5o)

This compound was obtained as yellow crystals in 64% yield, m.p. 232.5–233.3 °C. ^1^H NMR (400 MHz, CDCl_3_) *δ*: 8.50 (d, *J* = 5.2 Hz, 1H), 8.49 (d, *J* = 5.2 Hz, 1H), 8.16 (t, *J* = 7.6 Hz, 2H), 7.96 (d, *J* = 5.2 Hz, 1H), 7.94 (d, *J* = 5.2 Hz, 1H), 7.55–7.59 (m, 2H), 7.29–7.38 (m, 5H), 7.23 (d, *J* = 8.4 Hz, 1H), 7.18 (d, *J* = 8.4 Hz, 1H), 7.00–7.02 (m, 1H), 6.96–6.98 (m, 1H), 6.90–6.94 (m, 2H), 3.74–3.84 (m, 7H), 0.95–1.01 (m, 4H). ^13^C NMR (100 MHz, CDCl_3_) *δ*: 159.87, 142.11, 141.94, 140.82, 138.52, 136.77, 134.54, 133.87, 130.72, 130.55, 128.60, 128.52, 128.10, 126.92, 126.55, 121.77, 121.69, 121.38, 121.25, 120.10, 120.03, 114.18, 113.58, 113.48, 110.04, 109.93, 55.37, 43.60, 43.59, 26.18, 26.10. HRMS calcd for C_37_H_31_N_4_OS [M + H]^+^ 579.2213, found 579.2209. Anal. calcd for C_37_H_30_N_4_OS: C, 76.79; H, 5.23; N, 9.68; S, 5.54; found C, 76.10; H, 5.50; N, 9.29; S, 5.16.

#### 1–(3,4-dimethoxyphenyl)-9–(4-(1–(2-thienyl)-β-carboline-9-yl)butyl)-β-carboline (5p)

This compound was obtained as yellow crystals in 49% yield, m.p. 217.2–218.4 °C. ^1^H NMR (400 MHz, CDCl_3_) *δ*: 8.51 (d, *J* = 5.2 Hz, 1H), 8.47 (d, *J* = 5.2 Hz, 1H), 8.18 (d, *J* = 7.6 Hz, 1H), 8.14 (d, *J* = 7.6 Hz, 1H), 7.96 (d, *J* = 5.2 Hz, 1H), 7.94 (d, *J =* 5.2 Hz, 1H), 7.55–7.59 (m, 2H), 7.22 (d, *J* = 8.4 Hz, 1H), 7.19 (d, *J* = 8.4 Hz, 1H), 7.03 (d, *J* = 2.0 Hz, 1H), 6.98 (dd, *J* = 3.2, 1.2 Hz, 1H), 6.96 (dd, *J* = 8.0, 2.0 Hz, 1H), 6.92 (dd, *J* = 5.2, 3.2 Hz, 1H), 6.85 (d, *J* = 8.4 Hz, 1H), 3.86 (s, 3H), 3.82(s, 3H),3.78–3.85 (m, 4H), 0.98–1.08 (m, 4H). ^13^C NMR (100 MHz, CDCl_3_) *δ*: 149.24, 148.63, 143.72, 141.92, 141.90, 140.81, 138.53, 138.26, 136.75, 134.54, 133.98, 132.30, 130.69, 130.51, 128.65, 128.41, 128.04, 126.86, 126.50, 121.77, 121.73, 121.68, 121.46, 121.23, 120.10, 119.98, 114.20, 113.57, 112.47, 110.63, 110.11, 109.88, 55.99, 55.97, 43.66, 43.59, 26.29, 26.22. HRMS calcd for C_38_H_33_N_4_O_2_S [M + H]^+^ 609.2319, found 609.2314. Anal. calcd for C_38_H_32_N_4_O_2_S: C, 74.98; H, 5.30; N, 9.20; S, 5.27; found C, 74.47; H, 5.24; N, 9.36; S, 5.02.

#### 1–(3,4,5-trimethoxyphenyl)-9–(4-(1–(2-thienyl)-β-carboline-9-yl)butyl)-β-carboline (5q)

This compound was obtained as yellow crystals in 65% yield, m.p. 226.5–227.8 °C. ^1^H NMR (400 MHz, CDCl_3_) *δ*: 8.50 (d, *J* = 5.2 Hz, 1H), 8.46 (d, *J* = 5.2 Hz, 1H), 8.19 (d, *J* = 7.6 Hz, 1H), 8.12 (d, *J* = 7.6 Hz, 1H), 7.99 (d, *J* = 5.2 Hz, 1H), 7.92 (d, *J* = 5.2 Hz, 1H), 7.56–7.60 (m, 2H), 7.28–7.36 (m, 2H), 7.17–7.24 (m, 3H), 6.96 (dd, *J =* 7.6, 1.2 Hz, 1H), 6.88 (dd, *J* = 5.2, 3.6 Hz, 1H), 6.71 (s, 2H), 3.80–3.87 (m, 4H), 3.82 (s, 3H), 3.79 (s, 6H), 1.03–1.12 (m, 4H). ^13^C NMR (100 MHz, CDCl_3_) *δ*: 153.07, 143.44, 141.91, 141.87, 140.71, 138.45, 138.40, 137.94, 136.61, 134.50, 133.86, 130.70, 130.66, 128.83, 128.62, 128.05, 126.82, 126.48, 121.80, 121.64, 121.39, 121.15, 120.17, 120.15, 114.22, 113.88, 110.19, 109.84, 106.65, 60.95, 56.24, 43.68, 26.36, 26.27. HRMS calcd for C_39_H_35_N_4_O_3_S [M + H]^+^ 639.2424, found 639.2422. Anal. calcd for C_39_H_34_N_4_O_3_S: C, 73.33; H, 5.37; N, 8.77; S, 5.02; found C, 73.37; H, 5.59; N, 8.65; S, 4.81.

#### 1–(2-chlorophenyl)-9–(4-(1–(2-thienyl)-β-carboline-9-yl)butyl)-β-carboline (5r)

This compound was obtained as yellow crystals in 52% yield, m.p. 225.4–227.2 °C. ^1^H NMR (400 MHz, CDCl_3_) *δ*: 8.52 (s, 1H), 8.50 (s, 1H), 8.16–8.20 (m, 2H), 8.01 (d, *J* = 5.2 Hz, 1H), 7.97 (d, *J* = 5.2 Hz, 1H), 7.56–7.62 (m, 2H), 7.27–7.38 (m, 7H), 7.19–7.25 (m, 2H), 7.06 (dd, *J =* 3.6, 1.2 Hz, 1H), 7.03 (dd, *J =* 5.2, 3.2 Hz, 1H), 3.88 (t, *J* = 7.2 Hz, 2H), 3.53–3.71 (m, 2H), 1.01–1.13 (m, 4H). ^13^C NMR (100 MHz, CDCl_3_) *δ*: 141.97, 141.52, 140.78, 140.60, 138.51, 138.41, 138.11, 136.77, 134.53, 134.06, 133.87, 131.34, 130.86, 130.07, 130.05, 129.33, 128.62, 128.54, 128.25, 127.03, 126.66, 126.63, 121.75, 121.29, 121.18, 120.19, 119.96, 114.33, 114.22, 109.97, 109.68, 43.59, 43.29, 26.42, 26.40. HRMS calcd for C_36_H_28_ClN_4_S [M + H]^+^ 583.1718, found 583.1720. Anal. calcd for C_36_H_27_ClN_4_S: C, 74.15; H, 4.67; N, 9.61; S, 5.50; found C, 73.73; H, 4.53; N, 9.63; S, 5.19.

#### 7-methoxy-1-methyl-9–(4-(1-methyl-β-carboline-9-yl)butyl)-β-carboline (5s)

This compound was obtained as colorless crystals in 74% yield, m.p. 188.7–189.4 °C. ^1^H NMR (400 MHz, DMSO-*d_6_*) *δ*: 8.23 (s, 1H), 8.21 (s, 1H),8.16 (d, *J* = 5.2 Hz, 1H), 8.08 (d, *J =* 8.8 Hz, 1H), 7.99 (d, *J* = 5.2 Hz, 1H), 7.87 (d, *J* = 5.2 Hz, 1H), 7.70 (d, *J =* 8.8 Hz, 1H), 7.54–7.58 (m, 1H), 7.22–7.26 (m, 1H), 7.20 (d, *J =* 2.0 Hz, 1H), 6.86 (dd, *J =* 8.8, 2.0 Hz, 1H), 4.58–4.62 (m, 4H), 3.87 (s, 3H), 2.94 (s, 3H), 2.89 (s, 3H), 1.82–1.86 (m, 4H). ^13^C NMR (100 MHz, DMSO-*d_6_*) *δ*: 160.38, 142.59, 141.14, 140.96, 140.39, 137.69, 137.49, 134.37, 134.23, 128.33, 127.97,127.94, 122.27, 121.36, 120.36, 119.36, 114.09, 112.83, 112.12, 110.27, 108.91, 93.74, 55.48, 43.67, 43.57, 27.55, 27.37, 22.98, 22.86. HRMS calcd for C_29_H_29_N_4_O [M + H]^+^ 449.2336, found 449.2340. Anal. calcd for C_29_H_28_N_4_O: C, 77.65; H, 6.29; N, 12.49; found C 77.39, H 5.97, N 12.38.

#### 7-methoxy-1-methyl-9–(5-(1-methyl-β-carboline-9-yl)pentyl)-β-carboline (5t)

This compound was obtained as colorless crystals in 67% yield, m.p. 176.8–177.6 °C. ^1^H NMR (400 MHz, CDCl_3_) *δ*: 8.33 (d, *J* = 5.2 Hz, 1H), 8.29 (d, *J* = 5.2 Hz, 1H), 8.11 (d, *J* = 7.6 Hz, 1H), 7.97 (d, *J =* 8.4 Hz, 1H), 7.84 (d, *J* = 5.2 Hz, 1H), 7.74 (d, *J* = 5.2 Hz, 1H), 7.52–7.56 (m, 1H), 7.36 (d, *J =* 8.4 Hz, 1H), 7.25–7.29 (m, 1H), 6.89 (dd, *J =* 8.4, 2.0 Hz, 1H), 6.77 (d, *J* = 2.0 Hz, 1H), 4.49 (t, *J* = 7.2 Hz, 2H), 4.42 (t, *J* = 7.2 Hz, 2H), 3.89 (s, 3H), 2.99 (s, 3H), 2.97 (s, 3H), 1.81–1.91 (m, 4H), 1.41–1.50 (m, 2H). ^13^C NMR (100 MHz, CDCl_3_) *δ*: 160.92, 143.09, 141.45, 141.04, 140.26, 138.15, 138.09, 135.17, 135.01, 129.58, 129.14, 128.21, 122.48, 121.53, 121.29, 119.73, 115.21, 112.97, 112.31, 109.56, 108.60, 93.51, 55.72, 44.55, 30.83, 30.56, 24.42, 23.58, 23.33. HRMS calcd for C_30_H_31_N_4_O [M + H]^+^ 463.2492, found 463.2497. Anal. calcd for C_30_H_30_N_4_O: C, 77.89; H, 6.54; N, 12.11; found C, 77.42; H, 6.33; N, 12.07.

#### 7-methoxy-1-methyl-9–(4-(1–(3-pyridyl)-β-carboline-9-yl)butyl)-β-carboline (5u)

This compound was obtained as colorless crystals in 70% yield, m.p. 163.8–164.5 °C. ^1^H NMR (400 MHz, CDCl_3_) *δ*: 8.79 (d, *J* = 2.0 Hz, 1H), 8.65 (dd, *J =* 4.8, 1.6 Hz, 1H), 8.51 (d, *J* = 5.2 Hz, 1H), 8.29 (d, *J* = 5.2 Hz, 1H), 8.15–8.17 (m, 1H), 7.94–7.96 (m, 2H), 7.71 (d, *J* = 5.2 Hz, 1H), 7.65–7.68 (m, 1H), 7.56–7.60 (m, 1H), 7.30–7.34 (m, 2H), 7.23–7.25 (m, 1H), 6.89 (dd, *J =* 8.4, 2.0 Hz, 1H), 6.60 (d, *J* = 2.0 Hz, 1H), 4.16 (t, *J =* 7.2 Hz, 2H), 3.95 (t, *J* = 7.2 Hz, 2H), 3.90 (s, 3H), 2.77 (s, 3H), 1.48–1.35 (m, 2H), 1.35–1.27 (m, 2H). ^13^C NMR (100 MHz, CDCl_3_) *δ*: 160.85, 150.02, 149.66, 142.77, 142.20, 140.30, 140.13, 139.02, 138.36, 136.42, 135.80, 135.01, 134.09, 130.92, 129.43, 128.81, 123.15, 122.45, 121.79, 121.30, 120.33, 115.16, 114.17, 112.29, 109.94, 108.54, 93.55, 55.73, 44.12, 43.84, 27.37, 26.02, 23.30. HRMS calcd for C_33_H_30_N_5_O [M + H]^+^ 512.2445, found 512.2453. Anal. calcd for C_33_H_29_N_5_O: C, 77.47; H, 5.71; N, 13.69; found C, 76.67; H, 5.75; N, 13.05.

#### 7-methoxy-1-methyl-9–(5-(1–(3-pyridyl)-β-carboline-9-yl)pentyl)-β-carboline (5v)

This compound was obtained as colorless crystals in 62% yield, m.p. 183.6–184.9 °C. ^1^H NMR (400 MHz, CDCl_3_) *δ*: 8.82 (dd, *J* = 2.0, 0.8 Hz, 1H), 8.54 (d, *J =* 5.2 Hz, 1H), 8.53 (dd, *J =* 5.2, 1.6 Hz, 1H) 8.30 (d, *J* = 5.2 Hz, 1H), 8.18 (d, *J* = 7.6 Hz, 1H), 8.02 (d, *J* = 5.2 Hz, 1H), 7.98 (d, *J* = 8.4 Hz, 1H), 7.85–7.88 (m, 1H), 7.75 (d, *J* = 5.2 Hz, 1H), 7.56–7.60 (m, 1H), 7.37 (d, *J* = 8.4 Hz, 1H), 7.30–7.34 (m, 1H), 7.24–7.28 (m, 1H), 6.90 (dd, *J =* 8.4, 2.0 Hz, 1H), 6.72 (d, *J* = 2.0 Hz, 1H), 4.27 (t, *J =* 7.6 Hz, 2H), 3.94 (t, *J =* 7.6 Hz, 2H), 3.91 (s, 3H), 2.90 (s, 3H), 1.45–1.53 (m, 2H), 1.33–1.42 (m, 2H), 0.87–0.90 (m, 2H). ^13^C NMR (100 MHz, CDCl_3_) *δ*: 160.95, 150.08, 149.51, 143.06, 142.01, 140.36, 140.19, 138.81, 138.05, 136.60, 135.99, 135.11, 134.25, 130.83, 129.58, 128.81, 123.05, 122.48, 121.74, 121.26, 120.26, 115.15, 114.25, 112.34, 110.00, 108.66, 93.56, 55.76, 44.43, 44.25, 30.09, 28.61, 23.82, 23.22 . HRMS calcd for C_34_H_32_N_5_O [M + H]^+^ 526.2601, found 526.2609. Anal. calcd for C_34_H_31_N_5_O: C, 77.69; H, 5.94; N, 13.32; found C, 77.76; H, 5.95; N, 13.74.

#### 7-methoxy-1-methyl-9–(4-(1–(2-thienyl)-β-carboline-9-yl)butyl)-β-carboline (5w)

This compound was obtained as colorless crystals in 68% yield, m.p. 189.3–190.1 °C. ^1^H NMR (400 MHz, CDCl_3_) *δ*: 8.50 (d, *J* = 5.2 Hz, 1H), 8.28 (d, *J* = 5.2 Hz, 1H), 8.14–8.16 (m, 1H), 7.96 (d, *J =* 1.6 Hz, 1H), 7.95 (d, *J* = 2.0 Hz, 1H), 7.72 (d, *J* = 5.2 Hz, 1H), 7.55–7.60 (m, 1H), 7.34–7.38 (m, 2H), 7.29–7.33 (m, 1H), 7.13 (dd, *J =* 3.6, 1.2 Hz, 1H), 7.02 (dd, *J* = 5.2, 3.6 Hz, 1H), 6.88 (dd, *J =* 8.4, 2.0 Hz, 1H), 6.66 (d, *J* = 2.0 Hz, 1H), 4.21 (t, *J* = 7.2 Hz, 2H), 4.17–4.08 (t, *J* = 7.2 Hz, 2H), 3.90 (s, 3H), 2.86 (s, 3H), 1.42–1.54 (m, 4H). ^13^C NMR (100 MHz, CDCl_3_) *δ*: 160.86, 142.92, 142.05, 140.94, 140.18, 138.72, 138.18, 136.91, 135.10, 134.60, 130.91, 129.54, 128.69, 128.26, 127.12, 126.70, 122.49, 121.75, 121.35, 120.22, 115.24, 114.21, 112.33, 109.94, 108.45, 93.68, 55.75, 43.94, 29.69, 27.70, 26.51, 23.28. HRMS calcd for C_32_H_29_N_4_OS [M + H]^+^ 517.2057, found 517.2063. Anal. calcd for C_32_H_28_N_4_OS: C, 74.39; H, 5.46; N, 10.84; S, 6.21; found C, 74.21; H, 5.42; N, 10.65; S, 6.17.

#### 7-methoxy-1-methyl-9–(5-(1–(2-thienyl)-β-carboline-9-yl)pentyl)-β-carboline (5x)

This compound was obtained as yellow crystals in 71% yield, m.p. 196.1–197.4 °C. ^1^H NMR (400 MHz, CDCl_3_) *δ*: 8.50 (d, *J* = 5.2 Hz, 1H), 8.30 (d, *J* = 5.2 Hz, 1H), 8.13–8.16 (m, 1H), 7.94–7.98 (m, 2H), 7.74 (d, *J* = 5.2 Hz, 1H), 7.55–7.59 (m, 1H), 7.39 (d, *J* = 8.4 Hz, 1H), 7.30 (m, 1H), 7.23 (dd, *J =* 5.2, 1.2 Hz, 1H), 7.16 (dd, *J =* 3.6, 1.2 Hz, 1H), 6.92 (dd, *J* = 5.2, 3.6 Hz, 1H), 6.89 (dd, *J =* 8.4, 2.0 Hz, 1H), 6.72 (d, *J* = 2.0 Hz, 1H), 4.28 (t, *J =* 7.6 Hz, 2H), 4.10 (t, *J =* 7.6 Hz, 2H), 3.90 (s, 3H), 2.92 (s, 3H), 1.50–1.58 (m, 2H), 1.40–1.48 (m, 2H), 0.96–1.04 (m, 2H). ^13^C NMR (100 MHz, CDCl_3_) *δ*: 160.86, 143.01, 142.10, 141.02, 140.33, 138.56, 138.18, 136.98, 135.20, 134.67, 130.84, 129.48, 128.66, 128.33, 126.89, 126.56, 122.44, 121.67, 121.28, 120.12, 115.23, 114.18, 112.32, 110.04, 108.52, 93.62, 55.73, 44.54, 44.09, 29.72, 28.94, 23.97, 23.38. HRMS calcd for C_33_H_31_N_4_OS [M + H]^+^ 531.2213, found 531.2213. Anal. calcd for C_33_H_30_N_4_OS: C, 74.69; H, 5.70; N, 10.56; S, 6.04; found C, 74.06; H, 6.08; N, 10.49; S, 6.44.

## Biological evaluation

### *In vitro* cell growth inhibition assay

Target compounds were assayed by the MTT method for cytotoxic activity, as described previously^5^. The panel of cell lines included gastric carcinoma (BGC-823), liver carcinoma (HepG2), breast carcinoma (MCF-7), colon carcinoma (HT-29), esophageal carcinoma (Eca-109), and Lewis lung carcinoma (LLC). Growth inhibition rates were calculated with the following equitation: Inhibition ratio (%)= $\frac{{OD}_{\text{compd}}-{\text{OD}}_{\text{blank}}}{{OD}_{\text{DMSO}}-{\text{OD}}_{\text{blank}}}\times 100\%$. Half maximal inhibitory concentration (IC_50_) of each compound was calculated using software Graph-Pad Prism (version 6.0).

### Assay of acute toxicities

Specific pathogen-free KM mice (6–8 weeks old) weighing 19–22 g were housed in a mouse room at 24 ± 2 °C and 60–70% humidity with 12 h light/dark cycles. The mice were provided rodent laboratory chow pellets and tap water for a week to adapt to the environment of the mouse room. The experimental protocol was approved by the Institutional Animal Ethical Committee, and all of the animals were provided by Laboratory Animal Center of Xinjiang Uygur Autonomous Region. Prior to each experiment, mice were fastened overnight and allowed free access to water. Various doses of the asymmetric dimeric β-carboline derivatives, ranging from 5.0 to 500 mg kg^−1^ dissolved in 0.5% carboxymethyl cellulose sodium (CMC-Na) salt solution, were given intraperitoneally to different groups of healthy KM mice, and each group contained 10 mice (5 males and 5 females). After the administration of the compounds, the mice were observed continuously for the first 2 h for any gross behavioral changes and deaths, then intermittently for the next 24 h, and occasionally thereafter for 14 days, and for the onset of any delayed effects. All of the animals were killed on the 14th day after drug administration, and they were checked macroscopically for possible damage to the heart, liver, and kidneys. Mice that experienced immediate death following drug administration were also examined for any possible organ damage. LD_50_ values were calculated graphically as described^34^.

### *In vivo* antitumor activity

Sarcoma180 and Lewis lung cancer cell lines were provided by Shanghai Institute of Pharmaceutical Industry. Mice were inoculated with Sarcoma180 and Lewis lung cancer tumour cells. After 7 days, the tumours were removed and the cells were harvested. Mice received subcutaneous injections of viable tumour cells (2 × 10^6^ cells/mouse) in the armpit. Each compound was administered via i.p. injection to different groups of mice (each group contained 10 female mice) 24 h after the inoculation at a dosage about one-fifth of the LD_50_ value once a day for seven consecutive days. This dose was the maximum tolerated dose for most of the compounds based on our preliminary studies. CTX at 30 mg kg^−1^ was used as the positive control and the vehicle as the negative control. The weight of the animals was recorded every three days. All of the animals were killed on the 21st day after tumour inoculation, and the tumours were excised and weighed. The inhibition rate was calculated as follows:

(C−T)/C×100

where *T* is the average tumor weight of the treated group and *C* is the average tumor weight of the negative control group.

### CAM assay in vivo

Anti-angiogenic activity of the selected compounds **5b** and **5w** were investigated *in vivo* using a CAM assay. Five-day-old fertilized eggs were obtained from a local hatchery. We injected 5 ml of albumin, and the eggs were incubated horizontally to allow the CAM to detach from the shell to produce a sham chamber. Compounds **5b** and **5w** were prepared in gelatin sponge discs (5 × 5 × 5 mm^3^) at the concentration of 0.5, 5.0, and 50 µM/disc, respectively. CA4P was used as the positive control drug. Discs containing the vehicle only (DMSO) were used as the negative control. A small window opening was made in the shell, and the discs were directly applied onto the CAM. The square opening was covered with sterilized surgical tape and the embryos were incubated for 48 h at 38.5 °C. The CAMs were photographed under a dissecting microscope and blood vessels in each CAM were counted. The results are presented as a mean percentage of inhibition to the control ± SD, (*n* = 3).

## Results and discussion

### Chemistry

**Scheme 1.**
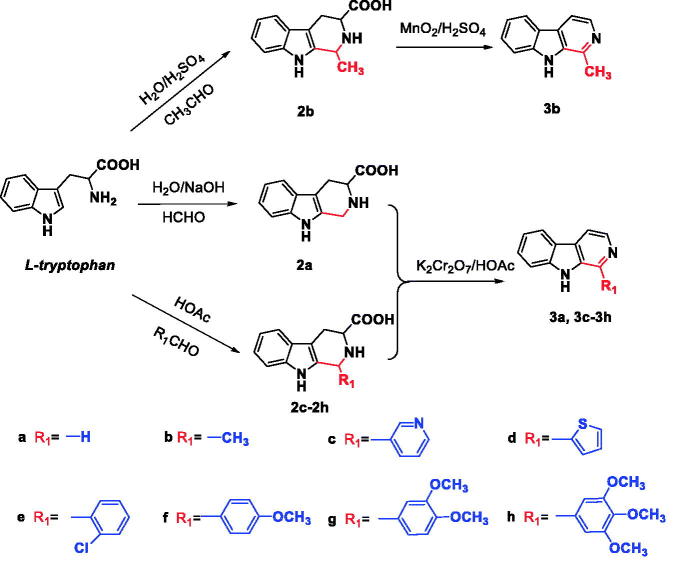
Synthetic route to compounds **3a-h**.

**Scheme 2.**
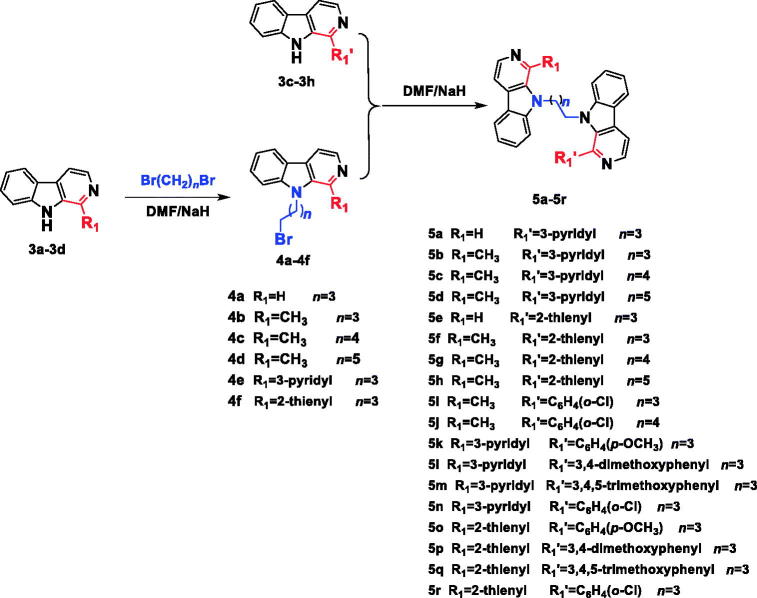
Synthesis of the *N*^9^-heterobivalent β-carbolines **5a-r**.

The synthesis of the desired *N*^9^-heterobivalent β-carbolines (**5a-r**) were performed in four steps starting from L-tryptophan as outlined in Scheme 1–2. The tetrahydro-β-carbolines **2a-h** was prepared by condensation of L-tryptophan with appropriate aldehyde via the Pictet-Spengler condensation, and followed by oxidation and decarboxylation to afford the intermediate 1-substituted-β-carbolines **3a-h**^21^^,^^29^. Then the *N^9^* of **3a-d** was alkylated by the action of sodium hydride (NaH) in *N*,*N*-dimethylformamide (DMF) followed by the addition of the appropriate dibromo alkane to obtain intermediates **4a-f**. Finally, **4a-f** with appropriate 1-substituted-β-carbolines **3c-h** in the presence of sodium hydride in DMF at room temperature to afford the target compounds **5a-r** in 47–74% yield.

**Scheme 3.**
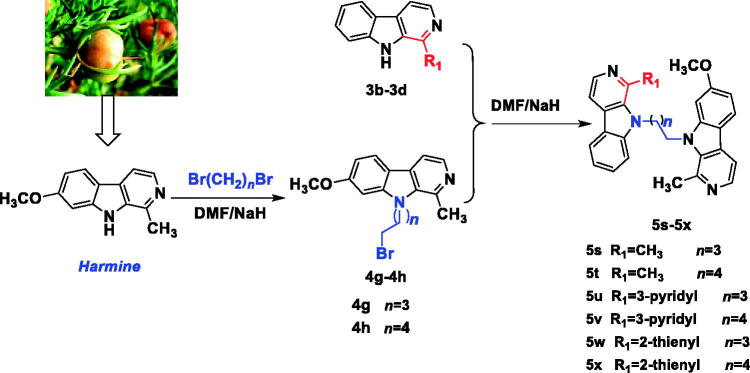
Synthesis of the *N*^9^-heterobivalent β-carbolines **5s-x**.

In **Scheme 3**, the intermediates **4g**-**f** were synthesized from harmine and 1,4-dibromobutane or 1,5-dibromopentane using the same method of compound **4a**, and finally compounds **5s-x** were obtained from the similar method of **5a**.

The chemical structures of all the novel *N*^9^-heterobivalent β-carbolines were characterized by ^1^H NMR, ^13^C NMR, HRMS and the elemental analysis.

### *In vitro* cytotoxicity and structure–activity relationships

All the 24 novel synthesized *N*^9^-heterobivalent β-carbolines (**5a-x**) were screened for their *in vitro* cytotoxic activities against six different cancer cell lines, namely BGC-823 (gastric carcinoma), HepG2 (liver carcinoma), MCF-7 (breast carcinoma), HT-29 (colon carcinoma), Eca-109 (esophageal carcinoma) and LLC (Lewis lung carcinoma). Cisplatin and B-9–3 were used as the reference control and the results were expressed as IC_50_ values and summarized in Table 1. The IC_50_ values were the average of at least three independent experiments.

**Table 1.** Cytotoxic activity of *N*^9^-heterobivalent β-carbolines *in vitro*


Compd.	R_1_	R_1_’	R_7_’	*N*	IC_50_ (μM) ±SD^a^					
					BGC^b^	HepG2	MCF7	HT-29	Eca-109	LLC
5a		H	H	3	>100	39.1 ± 5.4	>100	>100	>100	>100
5b		CH_3_	H	3	12.1 ± 2.3	15.9 ± 1.4	8.4 ± 1.7	12.6 ± 1.2	10.5 ± 1.3	12.4 ± 2.1
5c		CH_3_	H	4	22.6 ± 1.9	10.5 ± 2.1	15.3 ± 2.6	13.3 ± 2.4	25.3 ± 3.2	5.7 ± 1.2
5d		CH_3_	H	5	>100	>100	>100	>100	18.9 ± 3.1	14.5 ± 0.9
5e		H	H	3	>100	>100	>100	18.1 ± 3.6	>100	32.3 ± 4.5
5f		CH_3_	H	3	38.8 ± 2.9	>100	46.6 ± 4.8	56.1 ± 4.2	64.1 ± 7.6	6.1 ± 0.3
5g		CH_3_	H	4	97.6 ± 12.4	18.8 ± 2.3	75.5 ± 10.7	32.3 ± 5.3	30.8 ± 3.2	22.7 ± 1.4
5h		CH_3_	H	5	>100	>100	75.9 ± 9.4	47.8 ± 6.5	30.2 ± 3.4	30.2 ± 1.5
5i		CH_3_	H	3	37.9 ± 4.2	23.9 ± 3.7	95.5 ± 14.8	28.3 ± 1.6	23.4 ± 4.1	44.7 ± 3.6
5j		CH_3_	H	4	>100	>100	46.8 ± 3.7	24.1 ± 2.2	18.6 ± 2.1	16.6 ± 3.1
5k			H	3	>100	>100	>100	>100	>100	>100
	5l			H	3	>100	79.5 ± 12.3	>100	91.1 ± 10.6	93.2 ± 7.1	46.8 ± 6.4
	5m			H	3	>100	85.1 ± 11.6	>100	>100	70.9 ± 8.4	>100
	5n			H	3	>100	>100	86.9 ± 15.3	72.2 ± 5.2	>100	>100
	5o			H	3	>100	>100	>100	>100	>100	96.8 ± 9.8
	5p			H	3	>100	>100	>100	>100	>100	>100
	5q			H	3	>100	>100	>100	>100	>100	>100
	5r			H	3	>100	>100	60.7 ± 4.8	96.2 ± 5.1	96.7 ± 11.9	>100
	5s	CH_3_	CH_3_	OCH_3_	3	17.9 ± 1.1	37.6 ± 5.8	36.4 ± 2.1	11.1 ± 0.8	11.3 ± 2.7	6.2 ± 0.6
	5t	CH_3_	CH_3_	OCH_3_	4	42.7 ± 2.9	31.6 ± 2.2	17.6 ± 3.4	4.5 ± 0.6	51.9 ± 7.4	3.1 ± 0.4
	5u		CH_3_	OCH_3_	3	39.1 ± 2.4	26.8 ± 2.8	23.9 ± 0.7	15.1 ± 3.1	25.2 ± 5.5	12.2 ± 2.3
	5v		CH_3_	OCH_3_	4	23.2 ± 1.5	31.1 ± 3.4	18.7 ± 0.5	13.5 ± 2.2	13.2 ± 3.6	11.8 ± 1.8
	5w		CH_3_	OCH_3_	3	16.5 ± 1.9	15.4 ± 2.7	14.1 ± 1.4	13.1 ± 3.5	15.4 ± 1.9	13.1 ± 2.4
	5x		CH_3_	OCH_3_	4	>100	>100	41.6 ± 5.3	>100	46.7 ± 6.9	12.2 ± 3.7
B-9-3	CH_3_	CH_3_	H	3	22.3 ± 2.9	>100	13.2 ± 1.5	40.6 ± 5.8	14.5 ± 2.2	6.1 ± 0.9	
Cisplatin					11.6 ± 0.7	14.8 ± 0.4	12.4 ± 0.7	26.8 ± 1.4	8.9 ± 0.6	7.6 ± 0.4	

a Cytotoxicity as IC_50_ for each cell line, is the concentration of compound which reduced by 50% the optical density of treated cells with respect to untreated cells using the MTT assay. The data represent the mean values ± SD of at least three independent determinations. Values >100 μM indicate less than 50% growth inhibition at >100 μM.

b Cell lines include gastric carcinoma (BGC), liver carcinoma (HepG2), breast carcinoma (MCF-7), colon carcinoma (HT-29), esophageal carcinoma (Eca-109), and Lewis lung carcinoma (LLC).

As illustrated in Table 1, compounds **5b** and **5w** displayed a broad spectrum of cytotoxic activities with IC_50_ value of lower than 20 μM against the tested six tumor cell lines, while compounds **5c**, **5s**, **5t** and **5v** only exhibited strong cytotoxic effects with IC_50_ value of lower than 20 μM against three or four tumour cell lines. Interestingly, compounds **5d** and **5j** were selectively active against Eca-109 and LLC cell lines with IC_50_ value of lower than 20 μM but fail to show cytotoxic effects in other cell lines. Similarly, compound **5u** displayed selective activities against HT-29 and LLC cell lines. Moreover, compound **5f** only exhibited strong cytotoxic effects with IC_50_ value of lower than 20 μM against LLC cell lines. compounds **5g** and **5i** showed weak cytotoxic activities with IC_50_ values in the range of 18.8–97.6 μM. Unfortunately, compounds **5k**–**5r** were weak or inactive against all tumour cell lines tested.

When *N*^9^-heterobivalent β-carbolines had the same linker, we examined the influence of the substituents in position-1 of the β-carboline core on the cytotoxic activities. In order to enhance the range of substituents, we designed compounds have methyl and different pattern of substitution with an aryl ring substituted by electron withdrawing (Cl) and donating (OCH_3_) groups in position-1 of β-carboline. For example, compound **5a**, **5b**, and **5k**–**5n,** all have a 3-pyridyl group in R_1_ of one β-carboline ring, while in another β-carboline core, unlike compound **5a**, the substituted group of the position-1’ were methyl (**5b**), 4-methoxyphenyl (**5k**), 3,4-dimethoxyphenyl (**5l**), 3,4,5-trimethoxyphenyl (**5m**), 2-chlorophenyl (**5n**), respectively. Of these six dimeric β-carbolines, the compound **5b** displayed higher cytotoxic activities against BGC-823, HepG2, MCF-7, HT-29, Eca-109 and LLC with IC_50_ values of 12.1, 15.9, 8.4, 12.6, 10.5, and 12.4 μM, respectively. Additionally, among these six *N*^9^-heterobivalent β-carbolines **5e**, **5f**, and **5o**–**5r** bearing a 2-thienyl group in R_1_ of one β-carboline core, compound **5f** (R_1_’ = methyl) showed the highest cytotoxic activities against the test cell lines except HepG2. These results suggested that 3-pyridyl or 2-thienyl group substituent into position-1 of the β-carboline core, and in another β-carboline ring, the methyl substituent into R_1_’ facilitated cytotoxic potency, and the aryl substituent into R_1_’ might be detrimental to cytotoxic effects.

Next, we examined the influence of the spacer length of *N*^9^-heterobivalent β-carbolines on cytotoxic activities. According the previous investigation, the synthesized *N*^9^-heterobivalent β-carbolines were connected at the indolo-N by an alkyl chain, which ranged from 4 to 6 carbon atoms. Comparing the structure of **5b**–**5d**, which bearing different carbon atoms linker and 3-pyridyl group in position-1 of the β-carboline core, compound **5b** bearing 4-carbon atoms showed a broad spectrum of cytotoxic activities with IC_50_ value of lower than 20 μM against six tumor cell lines, and all the three compounds show selective cytotoxicities with IC_50_ value of lower than 20 μM against LLC. Similarly, further introduction of 2-thienyl group in position-1 of the β-carboline ring resulted in dimeric derivatives **5f**–**5h**. Among these derivatives, the IC_50_ values of compound **5f** against BGC, MCF7, and LLC cells is 38.8, 46.6, and 6.1 μM, respectively, compound **5 g** displayed IC_50_ values for HepG2 and HT-29 of 18.8 and 32.3 μM, respectively, and compound **5h** only exhibited higher cytotoxic effects with IC_50_ value of 30.2 μM against Eca-109 than other two compounds. Comparing the structure of **5i** with **5j**, compound **5i** showed weak or moderate cytotoxic activities against all tumor cell lines tested, and compound **5j** was selectively active against Eca-109 and LLC cell lines with IC_50_ value of lower than 20 μM but fail to show cytotoxic effects in BGC and HepG2 at the concentration of 100 μM. Comparing the structure of **5u** with **5v**, there is an extra methoxyl group attached to position-7 of the β-carboline, compound **5v** demonstrated the higher cytotoxic activities against the tested tumor cell lines than **5u** (except for HepG-2 cell line). Similarly, comparing the structure **5w** with **5x**, compound **5w** displayed the higher cytotoxic activities against the tested tumor cell lines than **5x** (except for LLC cell line). These results suggested that the length of the spacer had no obvious relationship with the cytotoxic activities against the tumor cell lines.

An overview of the cytotoxic activities data of all new synthesized *N*^9^-heterobivalent β-carbolines. 11 compounds were found to exhibit selective activity against LLC cell lines with IC_50_ value of lower than 20 μM. Moreover, Compound **5b**, **5s** displayed higher cytotoxic activities against at least four tumour cell lines than the prototype B-9–3, and compound **5c**, **5t**, **5v**, **5w** exhibited higher cytotoxic effects against three tumour cell lines than B-9–3.

In summary, a total analysis of the cytotoxic activities of *N*^9^-heterobivalent β-carbolines *in vitro* clearly suggest that: (1) *C*^1^-methylation and *C*^7^-methoxylation were favorable for increased activities; (2) 3-Pyridyl or 2-thienyl group substituent into position-1 of the β-carboline core, and the aryl (electron withdrawing and donating groups) substituent into position-1’ of another β-carboline ring might be detrimental to cytotoxic effects of this class of compounds.

### Assessment of acute toxicity

The LD_50_ values of the selected *N*^9^-heterobivalent β-carbolines in mice after intraperitoneal (i.p.) administration are shown in Table 2. All of the tested dimeric β-carbolines resulted in acute toxic manifestation but they did not cause any obvious neurotoxic effects, including tremors, twitch, jumping, and supination. The animals showed a decrease in locomotive activity after the administration of various bivalent β-carbolines. Death occurred mostly in the high dosage group within 4–8 h after injection. All of the surviving animals returned to normal within the next day. Autopsies of the animals that died during the course of experiment and the necropsy findings in the surviving animals at the end of the experimental period (14 days) revealed no obvious changes in any of the organs.

**Table 2.** Acute toxic effects of *N*^9^-heterobivalent β-carbolines in mice and antitumor activities of these compounds against mice bearing Sarcoma 180 and Lewis lung cancer

Comp.	Acute toxicity		Dosage (mg kg^−1^)	Tumor inhibition rate (%)^a^	
	LD_50_(mg kg^−1^)	Neurotoxic effect		Sarcoma 180	Lewis lung cancer
5b	150	–^b^	30	43.6 ± 8.9	41.9 ± 5.3
5c	175	–	35	33.6 ± 9.2	ND^c^
5t	35	+	7	37.2 ± 4.4	ND
5w	50	+	10	47.1 ± 6.2	42.3 ± 5.9
B-9-3 ^20^	200	–	40	56.2	40.4
CTX			30	82.5 ± 3.4	80.7 ± 2.1

a Data are expressed as mean ± SD.

b Acute neurotoxic manifestation were denoted by “+” and “–”. “+” represents toxic responses including tremble, twitch, jumping and supination, while “–” means no such reaction.

c ND = not determined.

Of all of the investigated asymmetric dimeric β-carbolines, compounds **5b** and **5c**, which had no substituent in position-7, and bearing different carbon atoms linker and 3-pyridyl group in position-1 of the β-carboline core, demonstrated weaker acute toxicities with the LD_50_ values of 150 mg kg^−1^ and 175 mg kg^−1^, respectively. Compounds **5t** and **5w**, which have a harmine molecular linked with another 1-substituted β-carboline ring, displayed remarkable acute toxicity with the LD_50_ values of 35 mg kg^−1^ and 50 mg kg^−1^, respectively. These results suggested that the methoxy substituent in position-7 of β-carboline nucleus played a vital role in determining the remarkable neurotoxic effects.

### Evaluation of antitumor activity of N^9^-heterobivalent β-carbolines *in vivo*

Based on the *in vitro* assay results, we further tested the antitumor activity of four *N*^9^-heterobivalent β-carbolines *in vivo* against mice bearing Sarcoma 180 and Lewis lung cancer, respectively, and the reference drugs Cyclophosphamide (CTX). Our previous investigation demonstrated that mice bearing Lewis lung cancer were more susceptible to β-carbolines than other animal models; therefore, these animal models were selected and evaluated in the present investigation.^5^ The tumor inhibition rates of all of the investigated asymmetric dimeric β-carbolines were summarized in Table 2.

As shown in Table 2, all the tested *N*^9^-heterobivalent β-carbolines displayed moderate to strong antitumor activities in animal model. Interestingly, compounds **5b** and **5c**, which having the same substituent and different carbon atoms linker. Compound **5b**, which bearing 4-carbon atoms, exhibited the potent antitumor agent with the tumor inhibition rate of 43.6 and 41.9% against Sarcoma 180-bearing mice and Lewis lung cancer-bearing mice, respectively, and compound **5c** displayed moderate antitumor activity with the tumor inhibition rate of 33.6% against Sarcoma 180-bearing mice at doses of 35 mg kg^−1^. Compound **5t**, the harmine and harman linked with five methylene units, showed moderate antitumor activity against mice with Sarcoma 180 with the tumour inhibition rate of 37.2% at doses of 7 mg kg^−1^. Particularly, compound **5w** was found to be the remarkable antitumor agent with the tumor inhibition rate of 47.1 and 42.3% against mice bearing Sarcoma 180 and Lewis lung cancer, respectively. These results implied that the harmine or harman linked with another of β-carboline nucleus (3-pyridyl or 2-thienyl substituent into position-1) enhanced their antitumor activities.

### *In vivo* anti-angiogenic effect of compounds 5b and 5w

**Figure 2.**
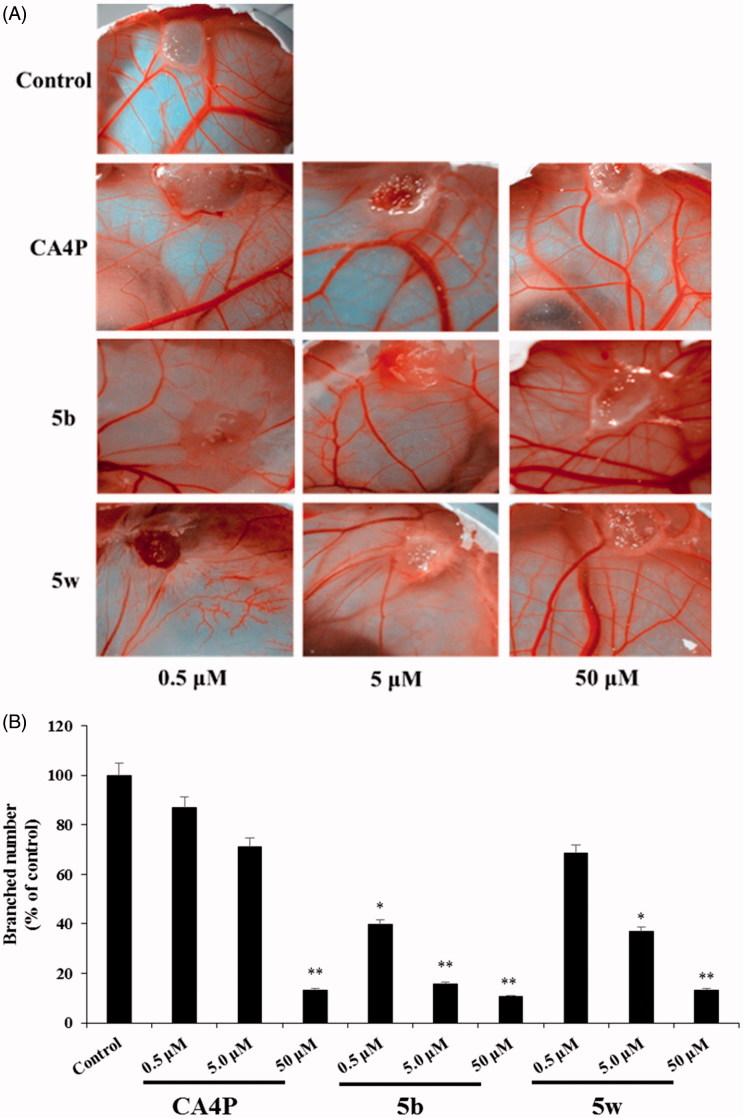
Compounds **5b** and **5w** inhibited angiogenesis. (A) *In vivo* anti-angiogenic effect of compounds **5b** and **5w** in chick chorioallantoic membrane (CAM) assay. positive control (CA4P, 0.5–50 mg/mL) and vehicle control (0.1% DMSO). (B) Quantification graphs of the inhibitory effects of compounds **5b** and **5w** on angiogenesis and migration. *p < 0.05, **p < 0.01.

The most potent compounds, **5b** and **5w**, were selected to evaluate anti-angiogenic activity by chicken chorioallantoic membrane (CAM) assay. The inhibitory effects of compounds **5b** and **5w** on the angiogenesis of CAM are shown in Figure 2(A). The anti-angiogenetic activity of compounds **5b** and **5w** were semi-quantitatively analyzed using Graph Pad Prism 5.0 (shown in Figure 2(B)). The results showed that compound **5b** (*p* < 0.05) could inhibit the angiogenesis of CAM. The anti-angiogenetic activity of compound **5b** was comparable to the CA4P *in vivo* CAM assay at the same dose (50 μM).

## Conclusions

In this study, we synthesized 24 new, *N*^9^-heterodimeric β-carboline derivatives and focused on compounds with 4 − 6 carbon linkers between the indole nitrogen. All of the compounds were screened for their *in vitro* cytotoxic activity against BGC-823, HepG2, MCF-7, HT-29, Eca-109 and LLC cancer cell lines. The results showed that compounds **5b**, and **5w** exhibited strong cytotoxic activities with IC_50_ value of lower than 20 μM against the six tested tumor cell lines. In addition, four asymmetric dimeric β-carbolines were selected for evaluation in vivo against mice bearing Sarcoma 180 and Lewis lung cancer, compounds **5b** and **5w** exhibited potent antitumor efficacies with tumour inhibition rate of over 40% in the tested animal models. Moreover, the pharmacological mechanisms showed that compound **5b** could retard in the CAM assay, and anti-angiogenetic potency was more potent than the reference drug CA4P. Preliminary structure-activity analysis indicated that: (1) *C*^1^-methylation and *C*^7^-methoxylation were favorable for increased activities; (2) 3-Pyridyl or 2-thienyl group substituent into position-1 of the β-carboline core, and the aryl (electron withdrawing and donating groups) substituent into position-1’ of another β-carboline ring might be detrimental to cytotoxic effects of this class of compounds. Although most *N*^9^-heterodimeric β-carbolines presented here showed modest cytotoxic activities, the investigations of these structural modifications and preliminary SARs would be helpful to further design and develop more potent compounds.

## Supplementary Material

9_9_-asymmetric_bivalent_carbolines_supporting_information.pdf
