# Supplementary material for: Synthesis and biological evaluation of novel N9-heterobivalent β-carbolines as angiogenesis inhibitors
Source: J Enzyme Inhib Med Chem. 2019 Jan 2;34(1):375–87. doi: 10.1080/14756366.2018.1497619 (PMC6327987; doi:10.1080/14756366.2018.1497619)
Supplement: 9_9_-asymmetric_bivalent_carbolines_supporting_information.pdf [file IENZ_A_1497619_SM6049.pdf]

Supplementary Information

Synthesis and biological evaluation of novel N^9-heterobivalent β-carbolines as angiogenesis inhibitors

Liang Guo[a], Qin Ma[b], Wei Chen[b], Wenxi Fan[b], Jie Zhang[a], Bin Dai[a,*]

[a]*School of Chemistry and Chemical Engineering, Key Laboratory for Green Processing of Chemical Engineering of Xinjiang Bingtuan, Shihezi University, Shihezi 832003, P R China*

[bc]*Xinjiang Huashidan Pharmaceutical Research Co. Ltd., 175 He Nan East Road, Urumqi 830011, P R China*

Contents

1. **Figure S1** the ^{1}H NMR and ^{13}C NMR spectrum of compound **4a** ············S3
2. **Figure S2** the ^{1}H NMR and ^{13}C NMR spectrum of compound **4b** ············S4
3. **Figure S3** the ^{1}H NMR and ^{13}C NMR spectrum of compound **4c** ············S5
4. **Figure S4** the ^{1}H NMR and ^{13}C NMR spectrum of compound **4d** ············S6
5. **Figure S5** the ^{1}H NMR and ^{13}C NMR spectrum of compound **4e** ············S7
6. **Figure S6** the ^{1}H NMR and ^{13}C NMR spectrum of compound **4f** ············S8
7. **Figure S7** the ^{1}H NMR and ^{13}C NMR spectrum of compound **5a** ············S9
8. **Figure S8** the ^{1}H NMR and ^{13}C NMR spectrum of compound **5b** ··········S10
9. **Figure S9** the ^{1}H NMR and ^{13}C NMR spectrum of compound **5c** ··········S11
10. **Figure S10** the ^{1}H NMR and ^{13}C NMR spectrum of compound **5d** ········S12
11. **Figure S11** the ^{1}H NMR and ^{13}C NMR spectrum of compound **5e** ········S13
12. **Figure S12** the ^{1}H NMR and ^{13}C NMR spectrum of compound **5f** ·········S14
13. **Figure S13** the ^{1}H NMR and ^{13}C NMR spectrum of compound **5g** ·········S15
14. **Figure S14** the ^{1}H NMR and ^{13}C NMR spectrum of compound **5h** ·········S16
15. **Figure S15** the ^{1}H NMR and ^{13}C NMR spectrum of compound **5i** ··········S17
16. **Figure S16** the ^{1}H NMR and ^{13}C NMR spectrum of compound **5j** ··········S18

17. **Figure S17** the ^{1}H NMR and ^{13}C NMR spectrum of compound **5k** ·······S19

18. **Figure S18** the ^{1}H NMR and ^{13}C NMR spectrum of compound **5l** ········S20

19. **Figure S19** the ^{1}H NMR and ^{13}C NMR spectrum of compound **5m** ······S21

20. **Figure S20** the ^{1}H NMR and ^{13}C NMR spectrum of compound **5n** ·······S22

21. **Figure S21** the ^{1}H NMR and ^{13}C NMR spectrum of compound **5o** ·······S23

22. **Figure S22** the ^{1}H NMR and ^{13}C NMR spectrum of compound **5p** ········S24

23. **Figure S23** the ^{1}H NMR and ^{13}C NMR spectrum of compound **5q** ········S25

24. **Figure S24** the ^{1}H NMR and ^{13}C NMR spectrum of compound **5r** ·········S26

25. **Figure S25** the ^{1}H NMR and ^{13}C NMR spectrum of compound **5s** ·········S27

26. **Figure S26** the ^{1}H NMR and ^{13}C NMR spectrum of compound **5t** ·········S28

27. **Figure S27** the ^{1}H NMR and ^{13}C NMR spectrum of compound **5u** ········S29

28. **Figure S28** the ^{1}H NMR and ^{13}C NMR spectrum of compound **5v** ········S30

29. **Figure S29** the ^{1}H NMR and ^{13}C NMR spectrum of compound **5w** ·······S31

30. **Figure S30** the ^{1}H NMR and ^{13}C NMR spectrum of compound **5x** ········S32

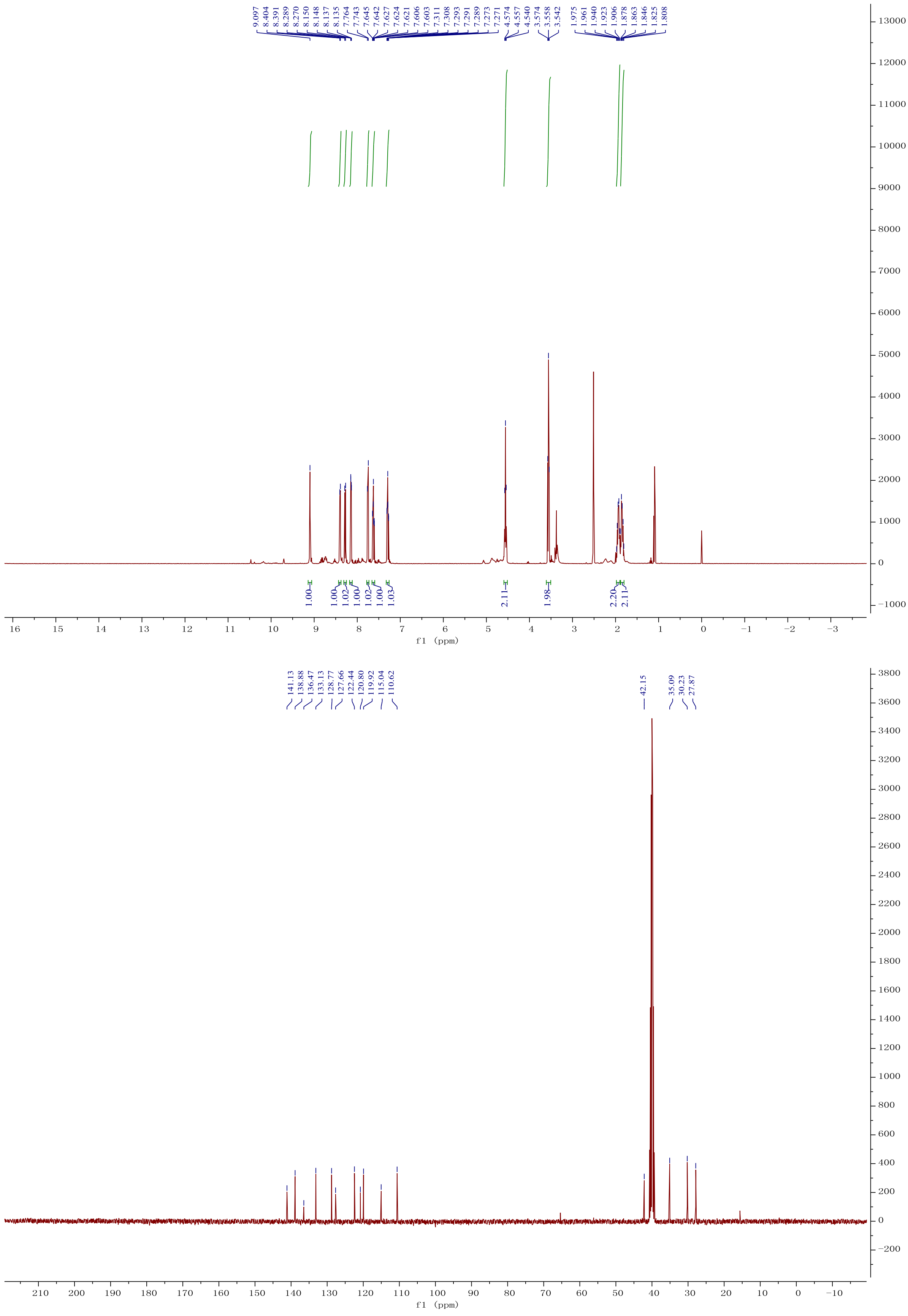

Figure S1 the ^{1}H NMR spectrum and ^{13}C NMR spectrum of compound **4a**

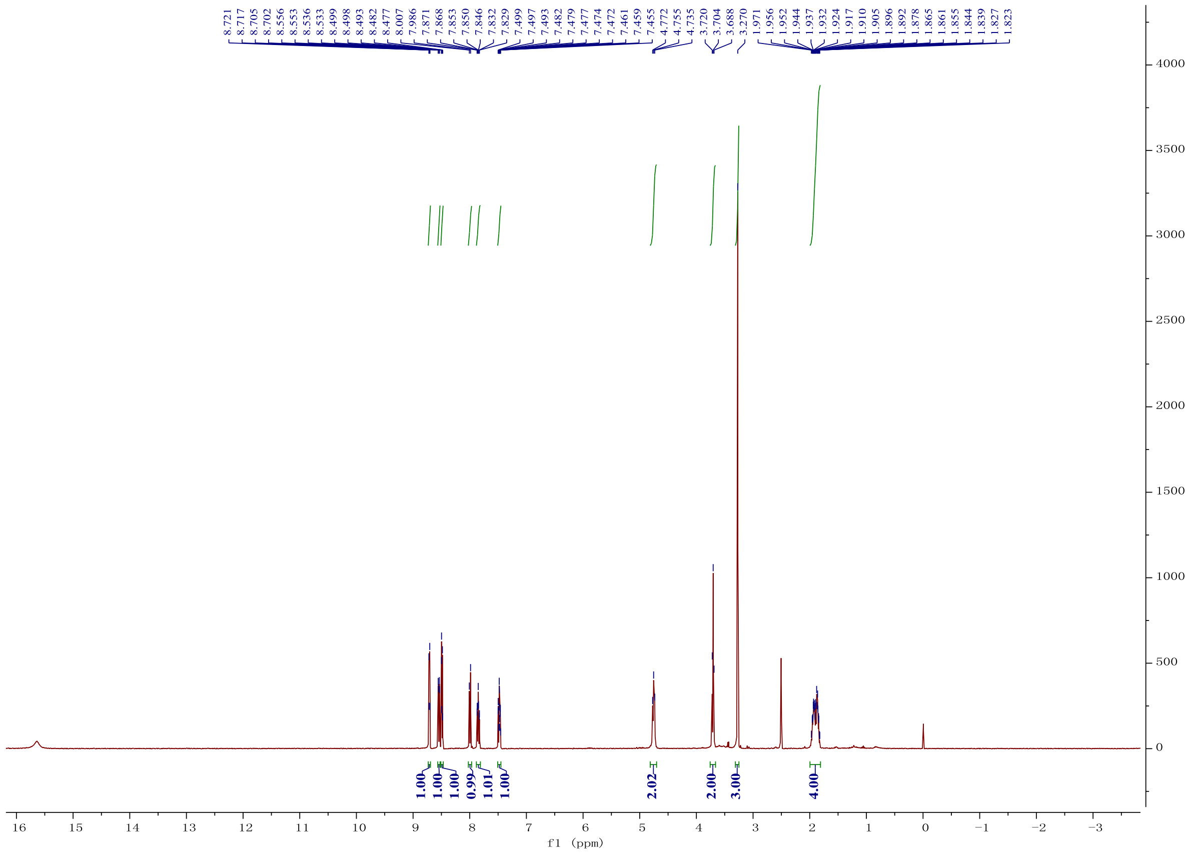

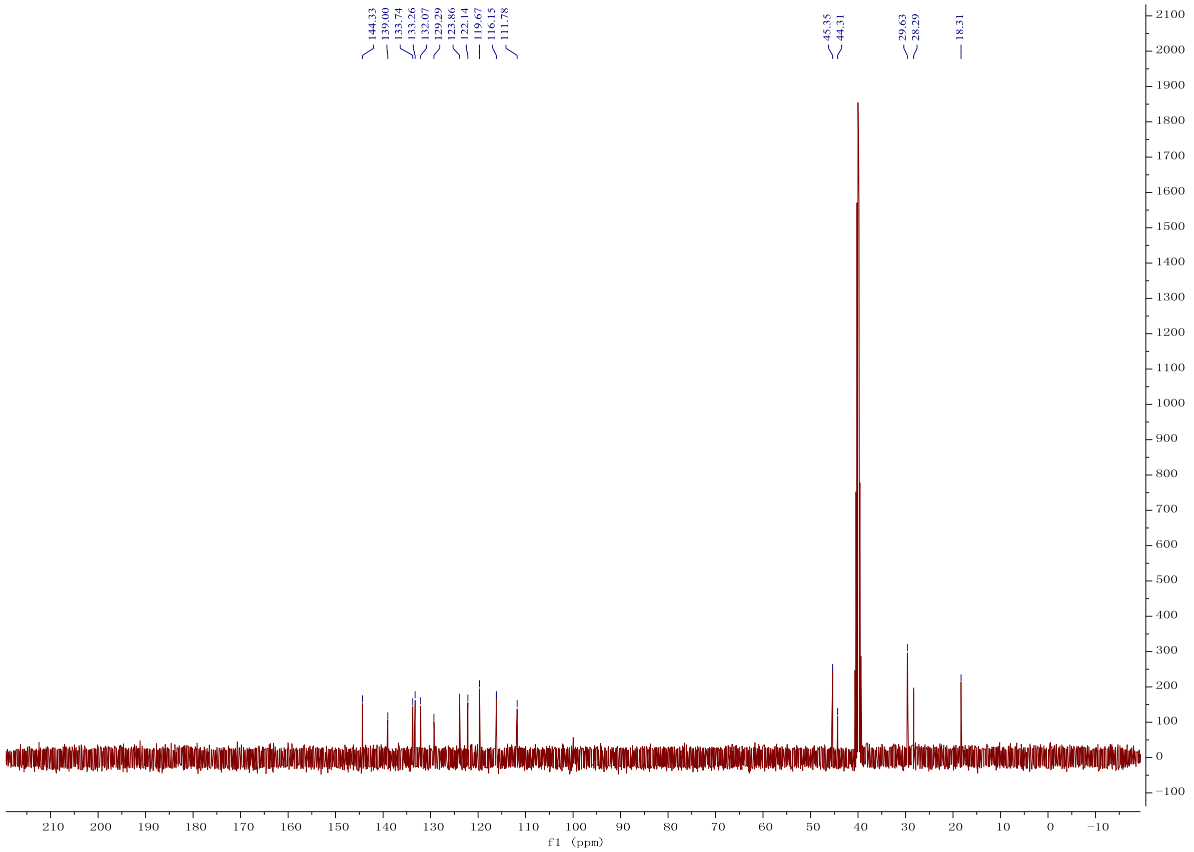

Figure S2 the ^{1}H NMR spectrum and ^{13}C NMR spectrum of compound **4b**

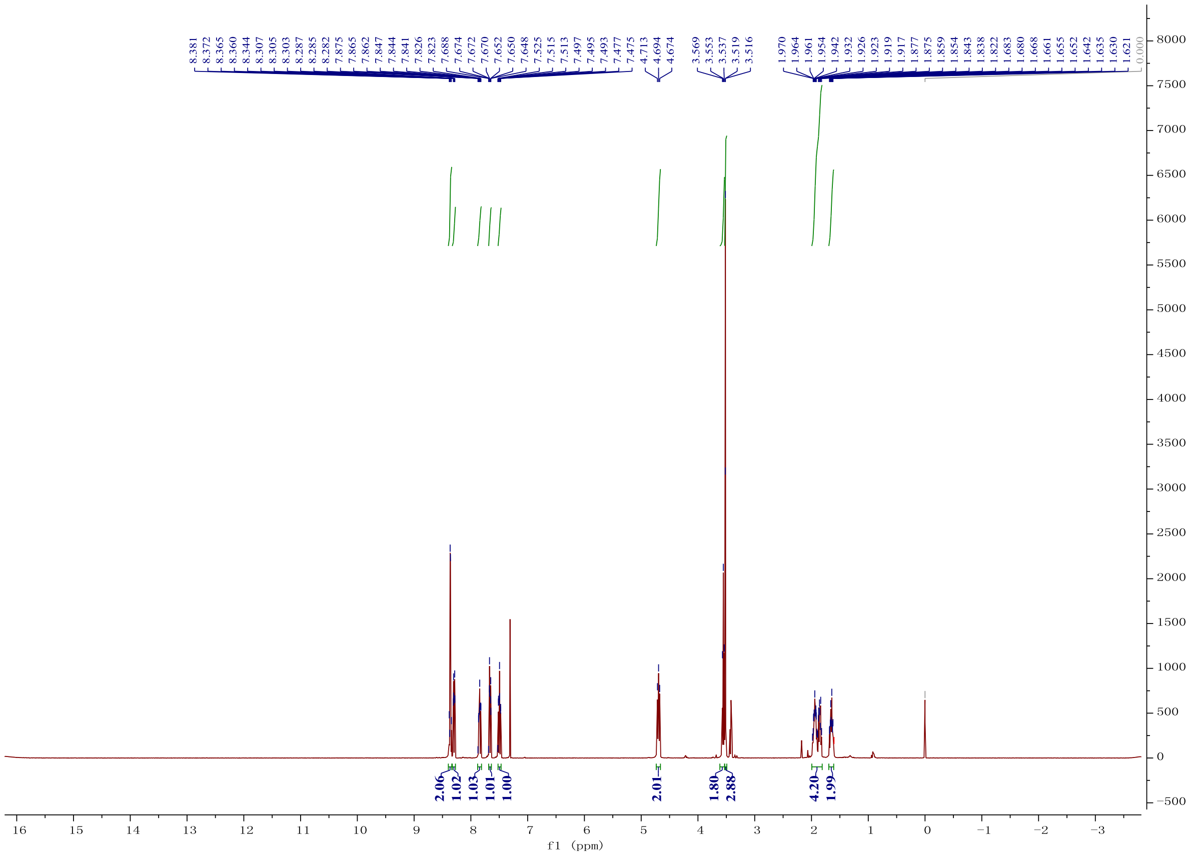

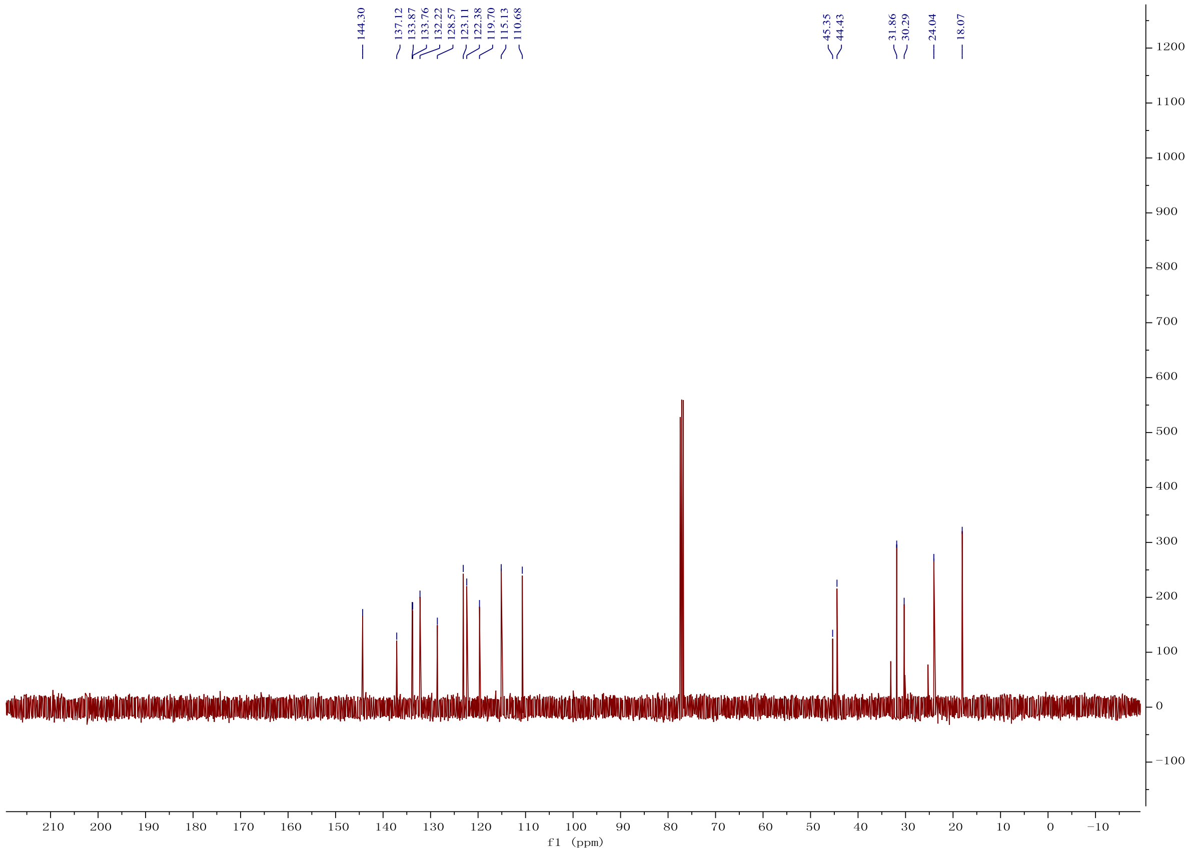

Figure S3 the ^{1}H NMR spectrum and ^{13}C NMR spectrum of compound **4c**

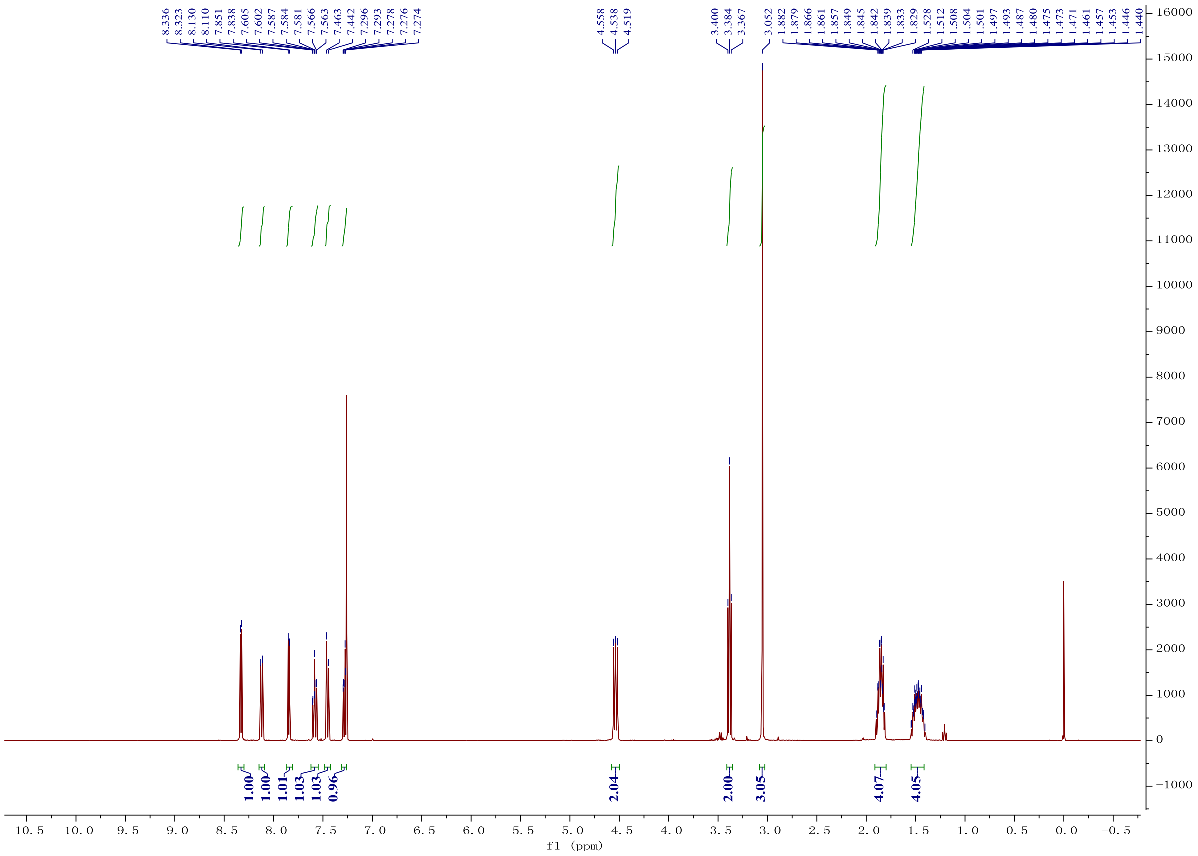

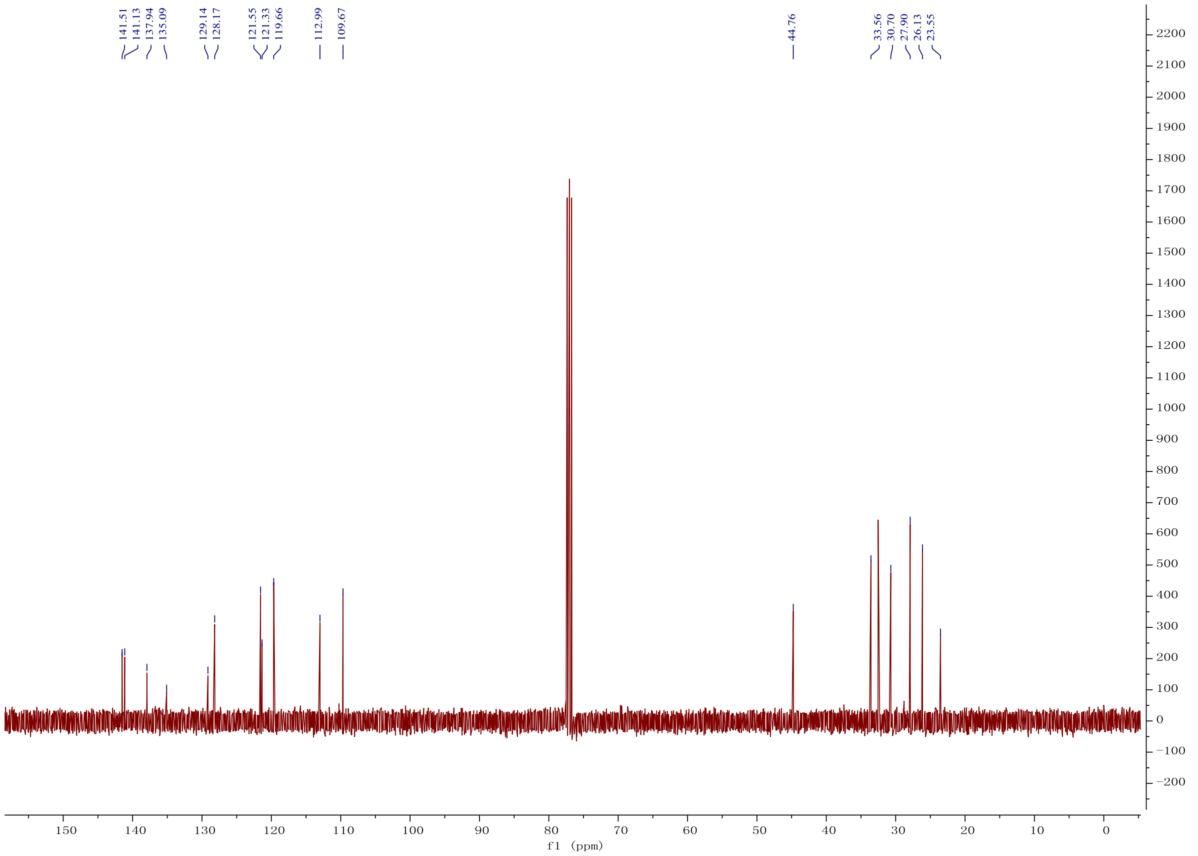

Figure S4 the ^{1}H NMR spectrum and ^{13}C NMR spectrum of compound **4d**

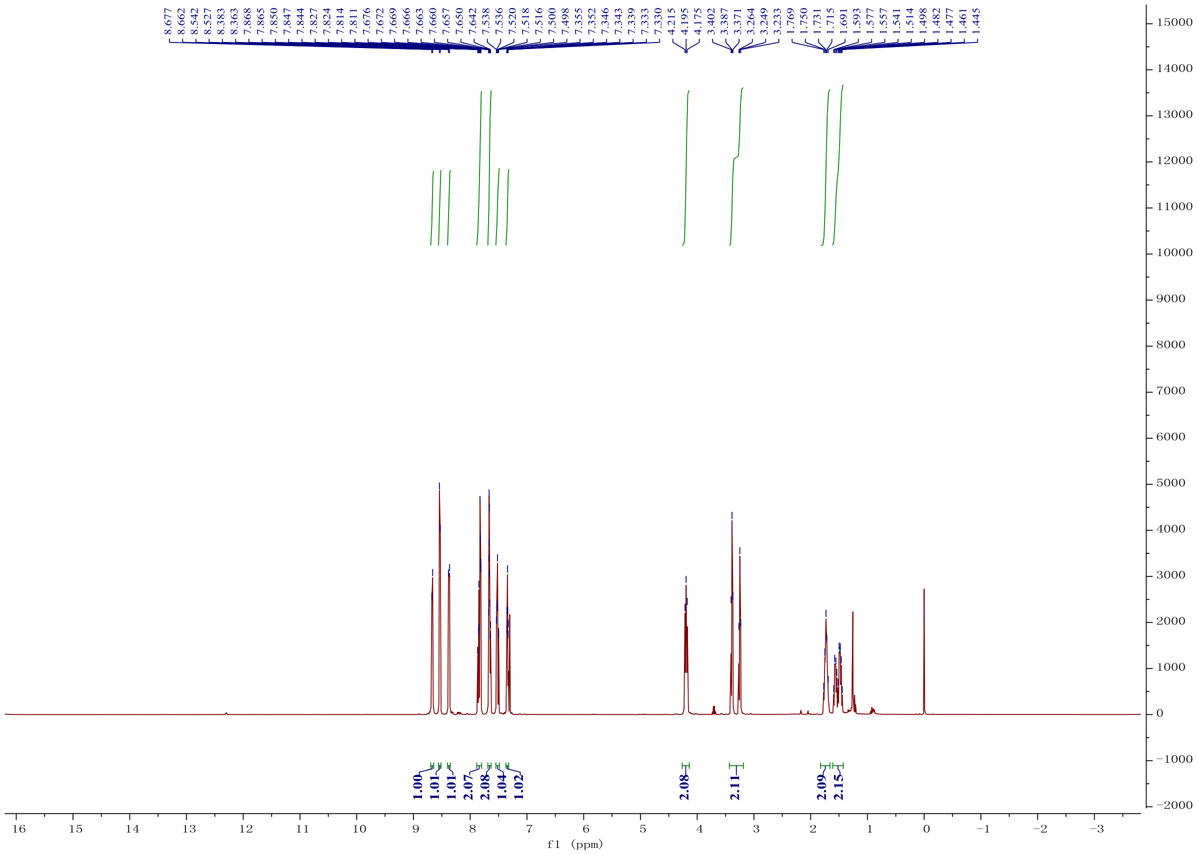

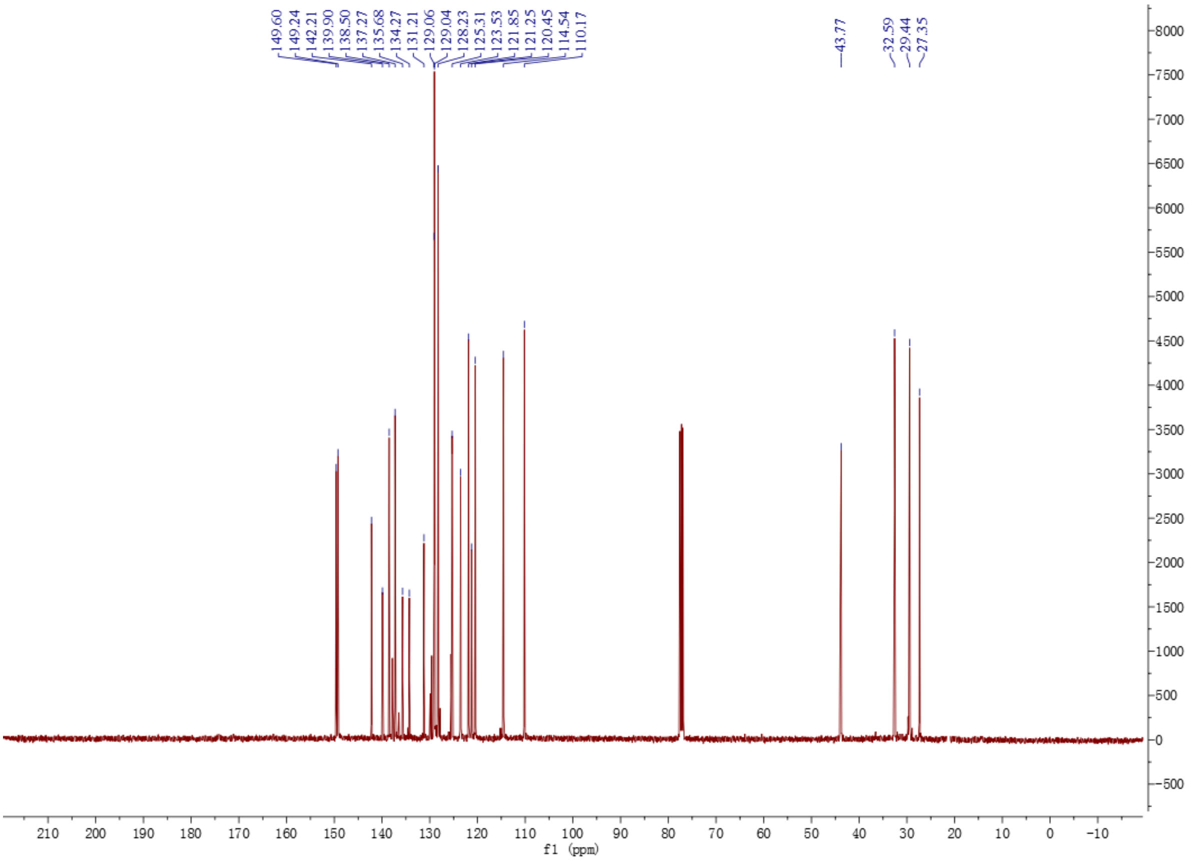

Figure S5 the ^{1}H NMR spectrum and ^{13}C NMR spectrum of compound **4e**

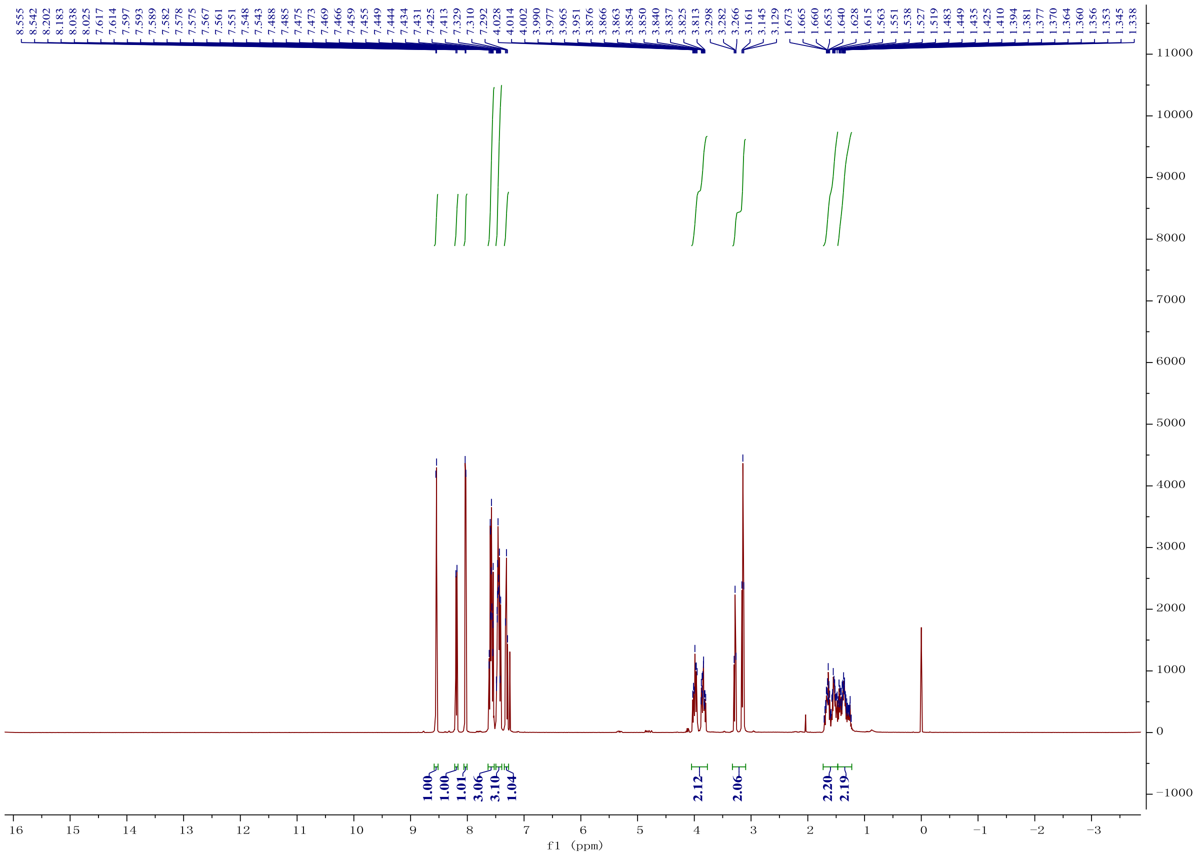

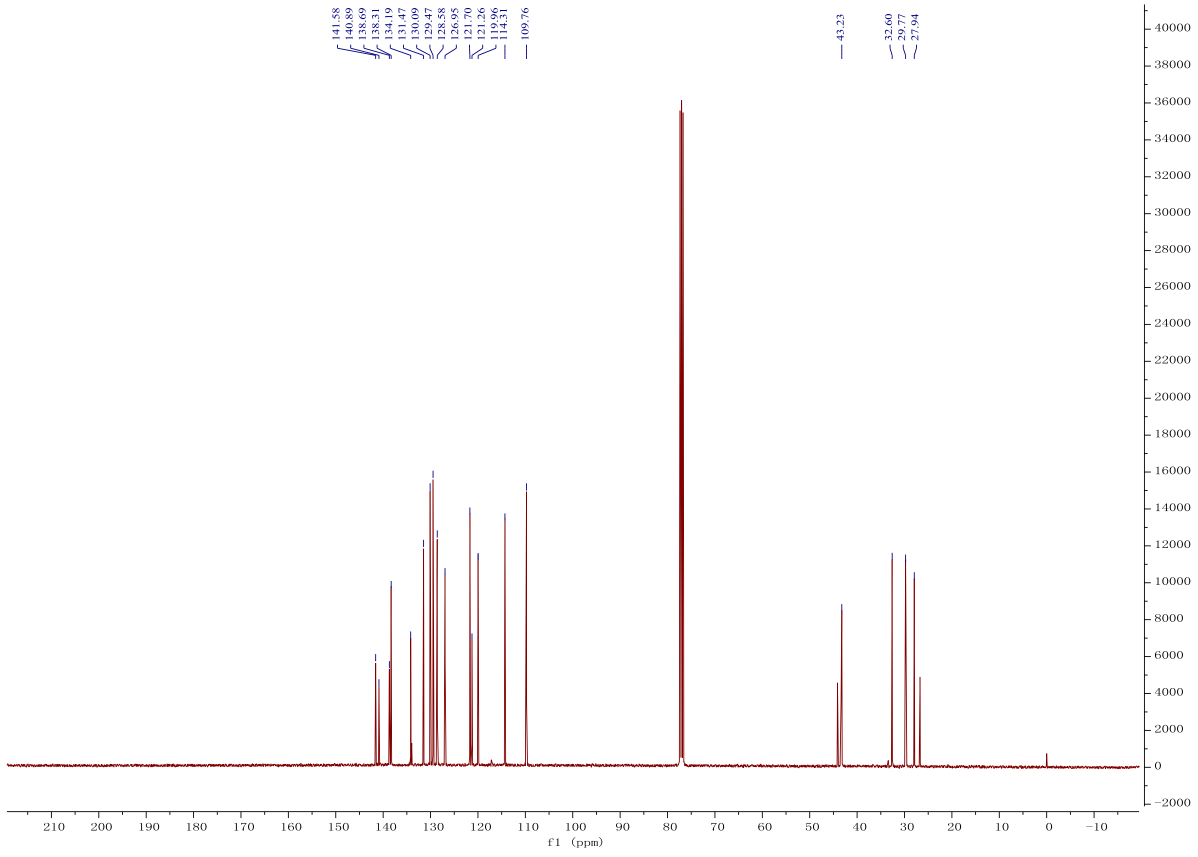

Figure S6 the ^{1}H NMR spectrum and ^{13}C NMR spectrum of compound **4f**

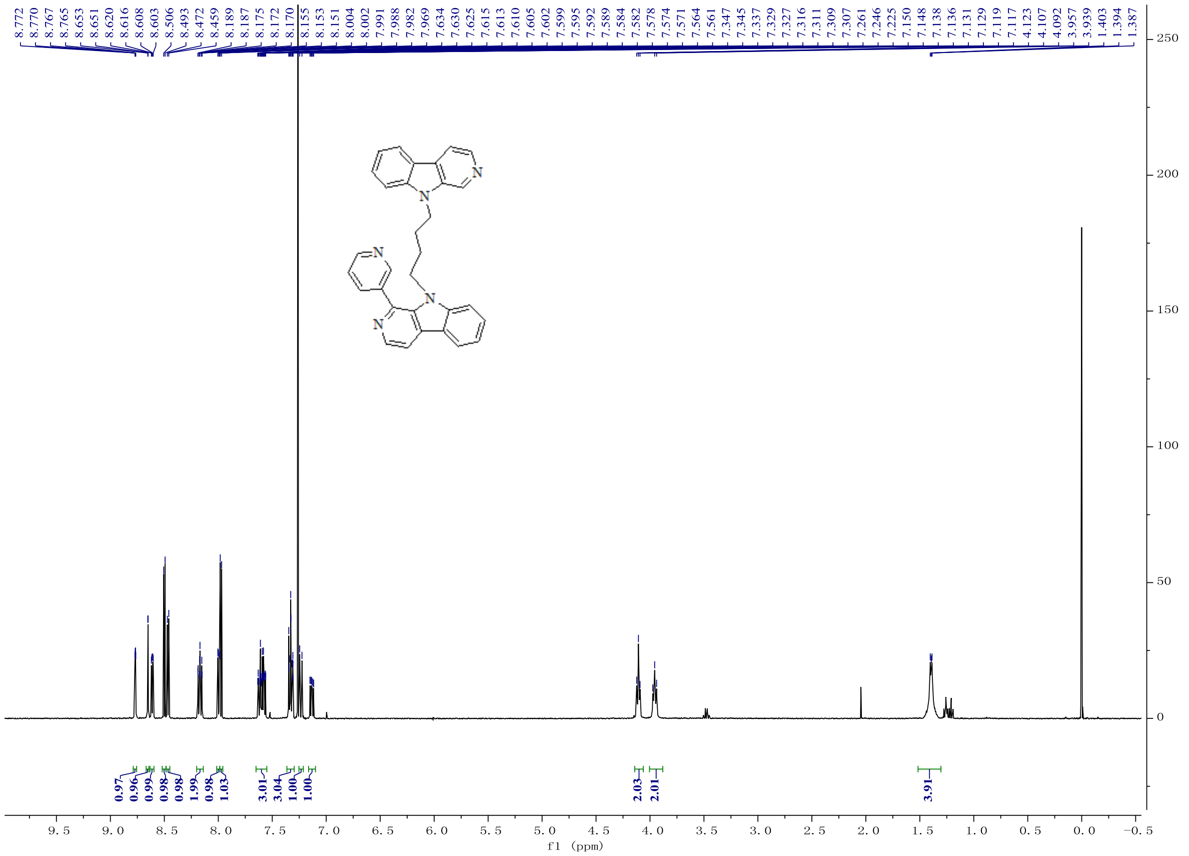

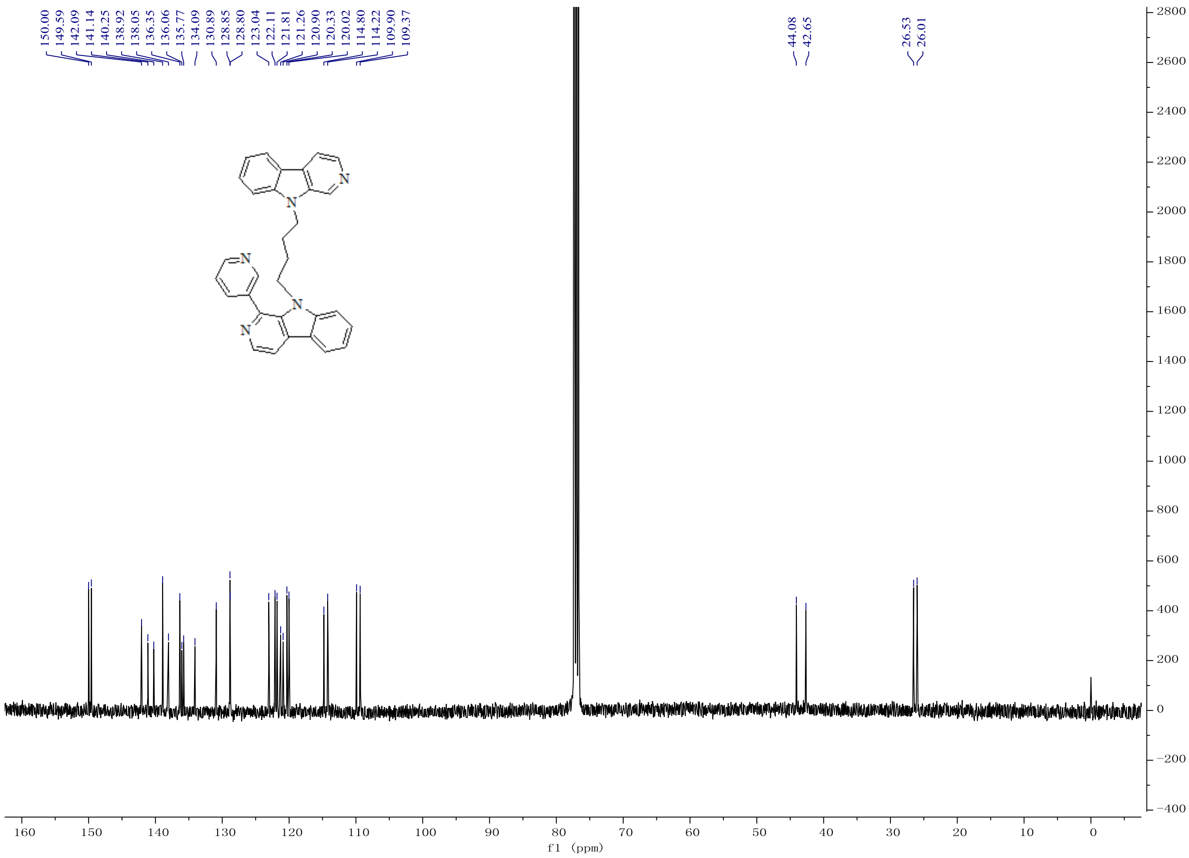

Figure S7 the ^{1}H NMR spectrum and ^{13}C NMR spectrum of compound **5a**

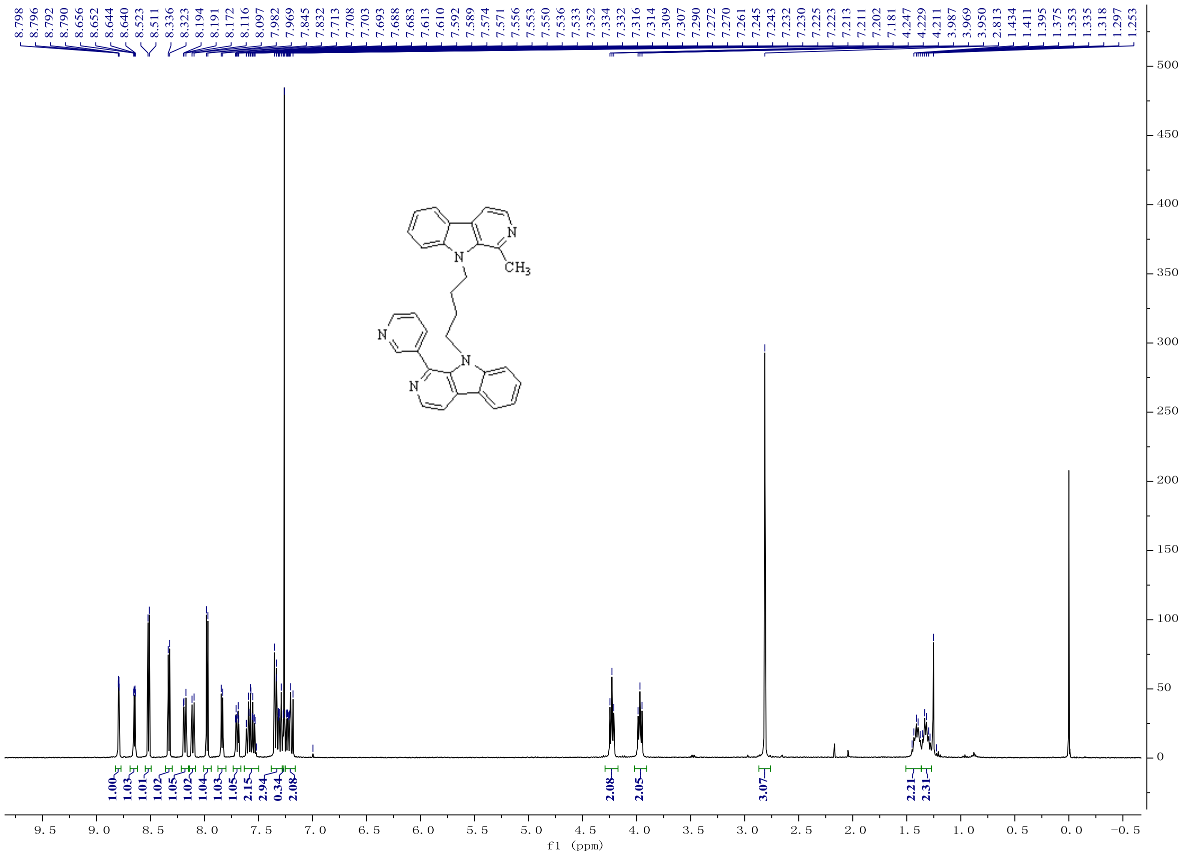

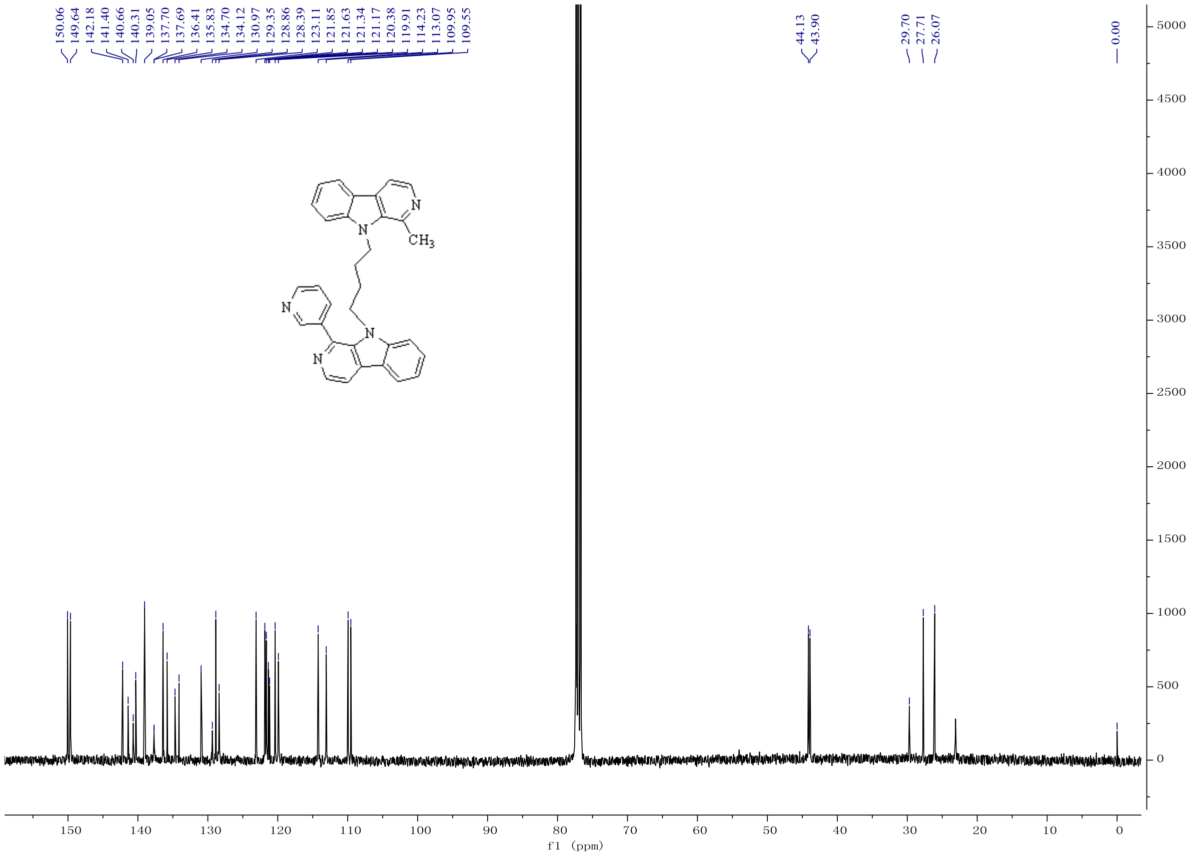

Figure S8 the ^{1}H NMR spectrum and ^{13}C NMR spectrum of compound **5b**

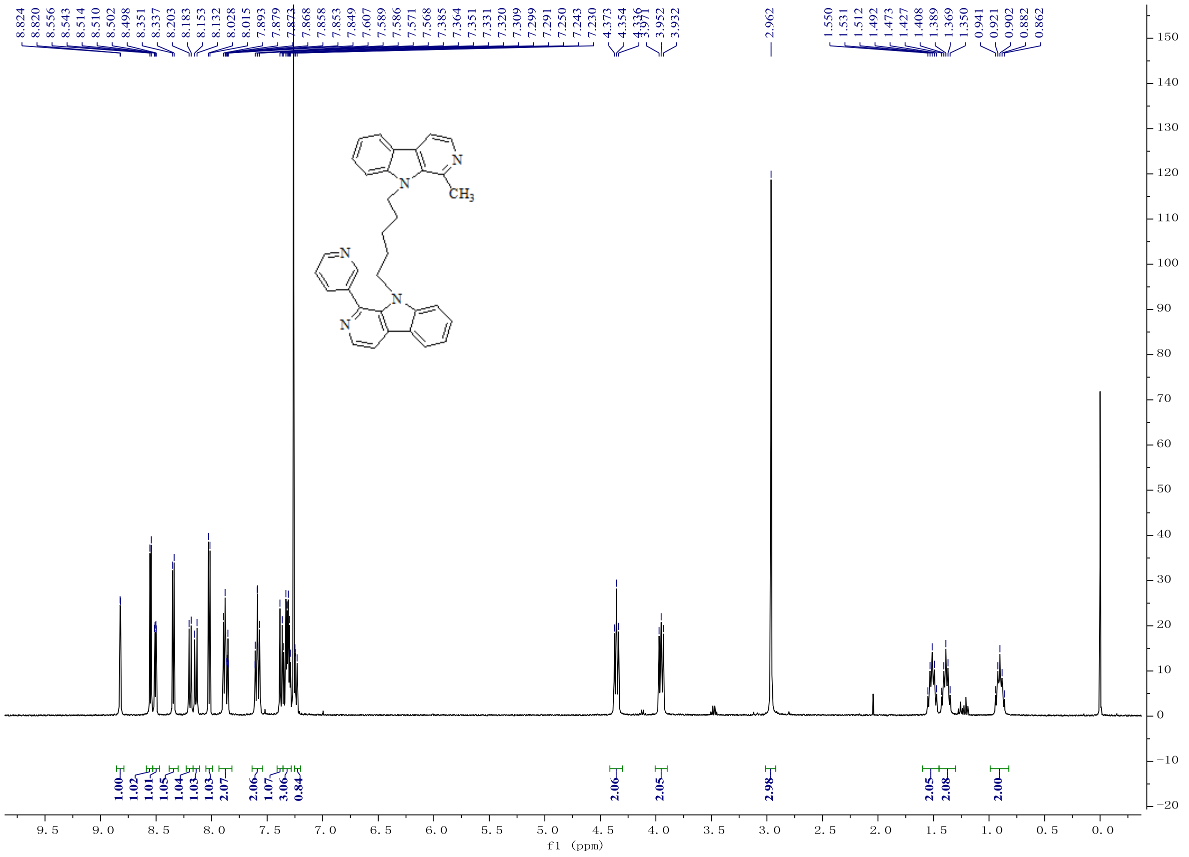

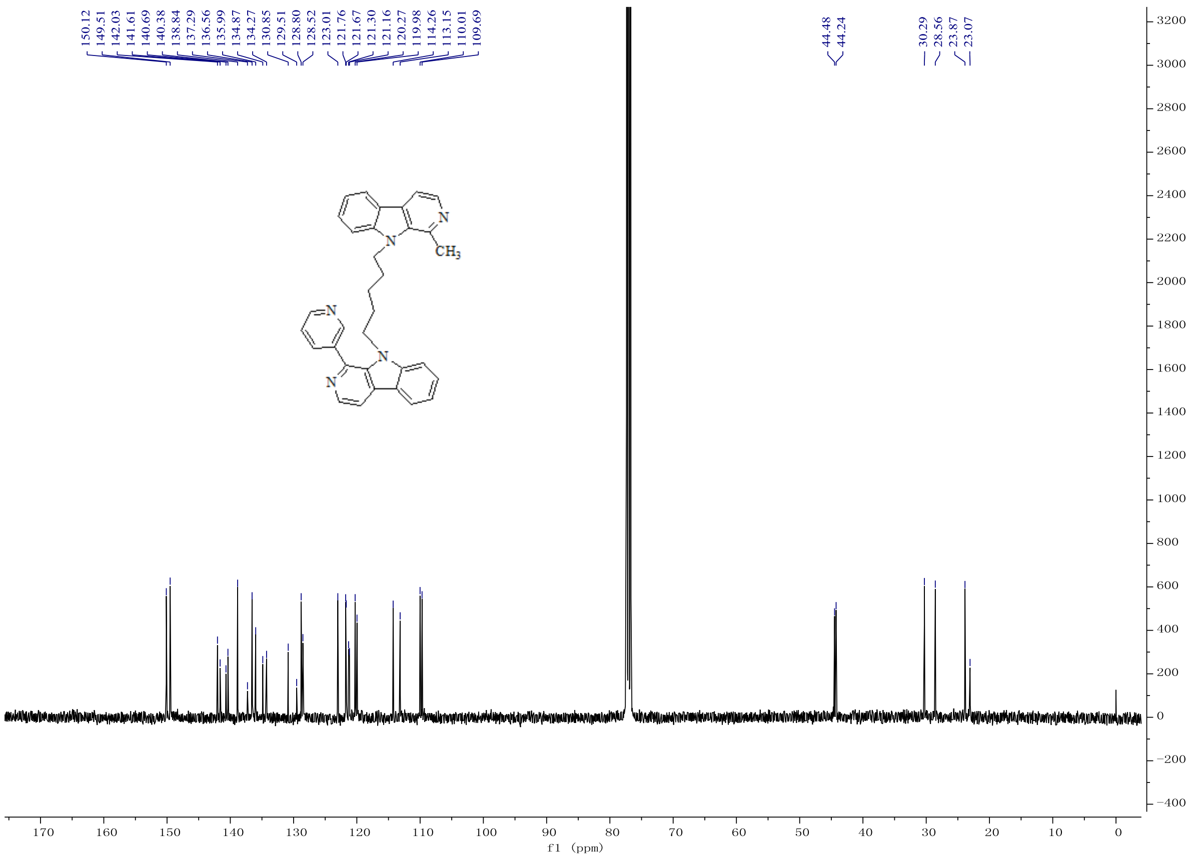

Figure S9 the ^{1}H NMR spectrum and ^{13}C NMR spectrum of compound **5c**

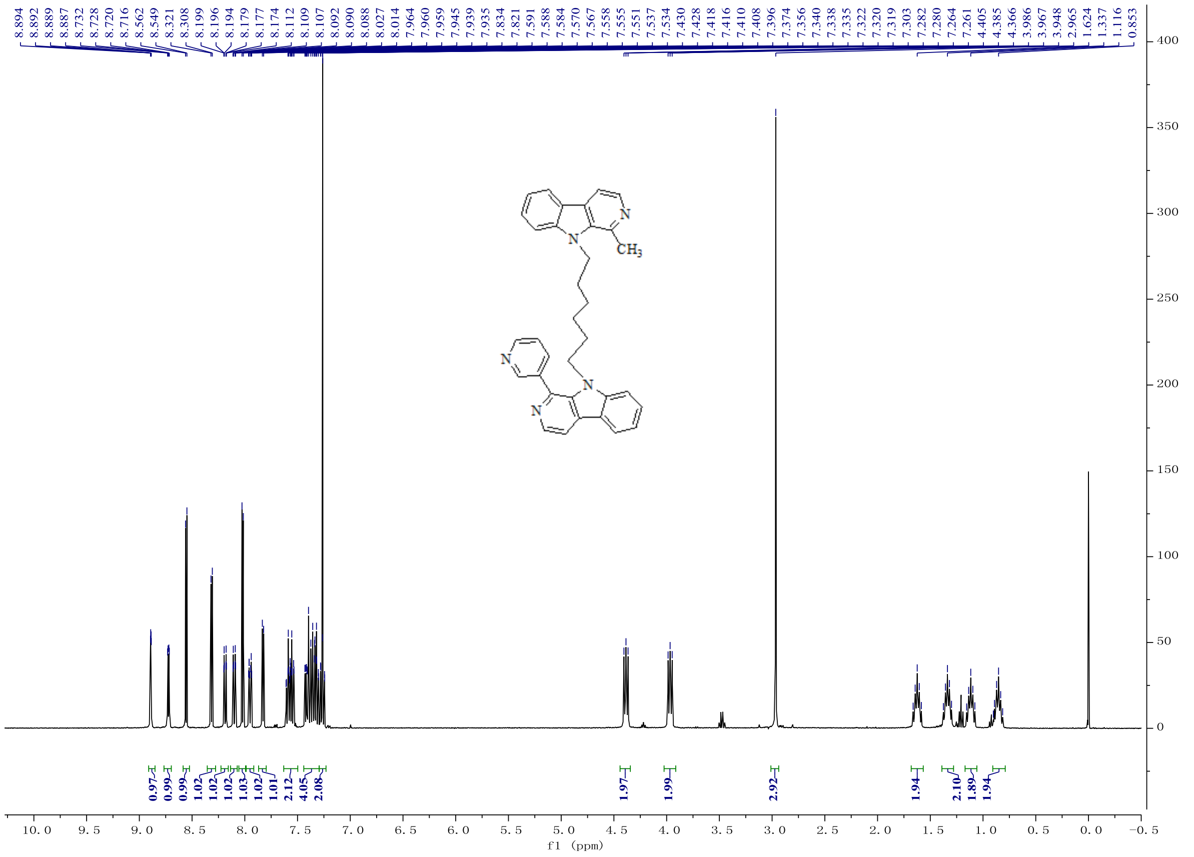

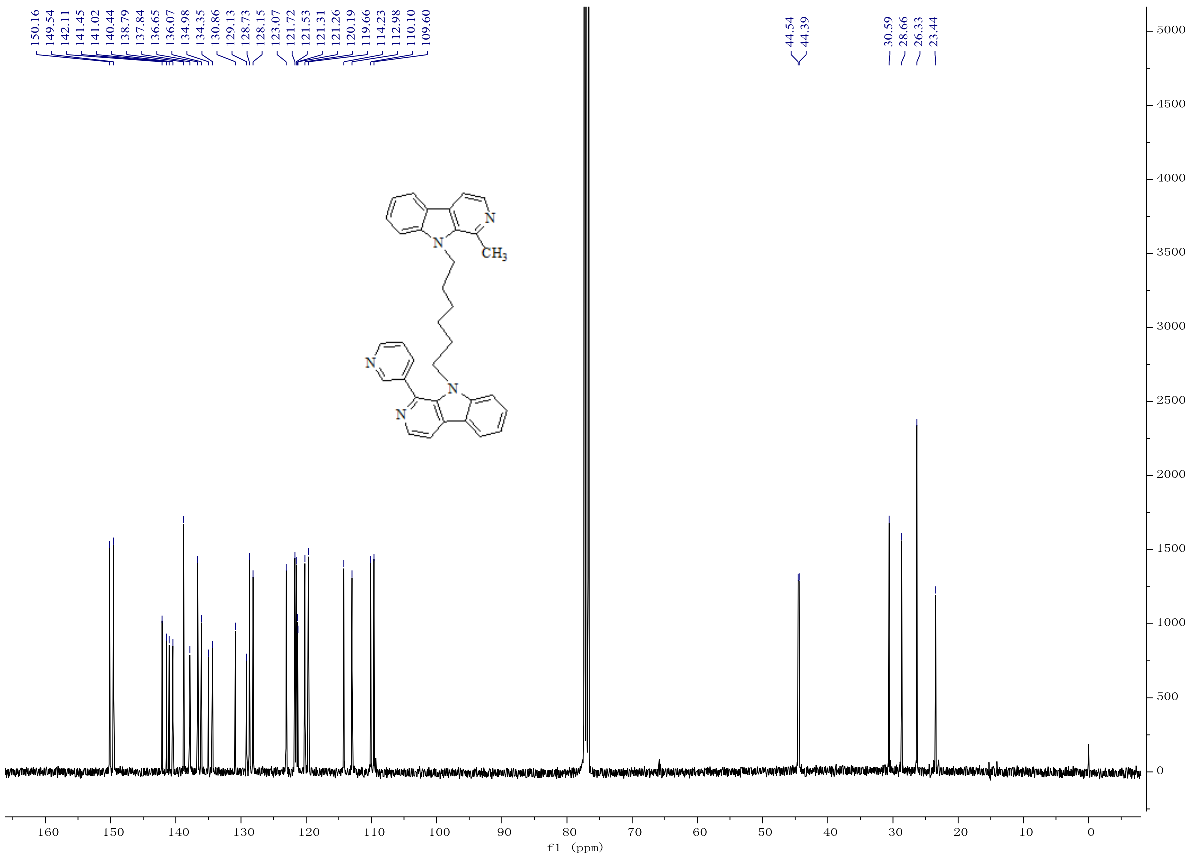

Figure S10 the ^{1}H NMR spectrum and ^{13}C NMR spectrum of compound **5d**

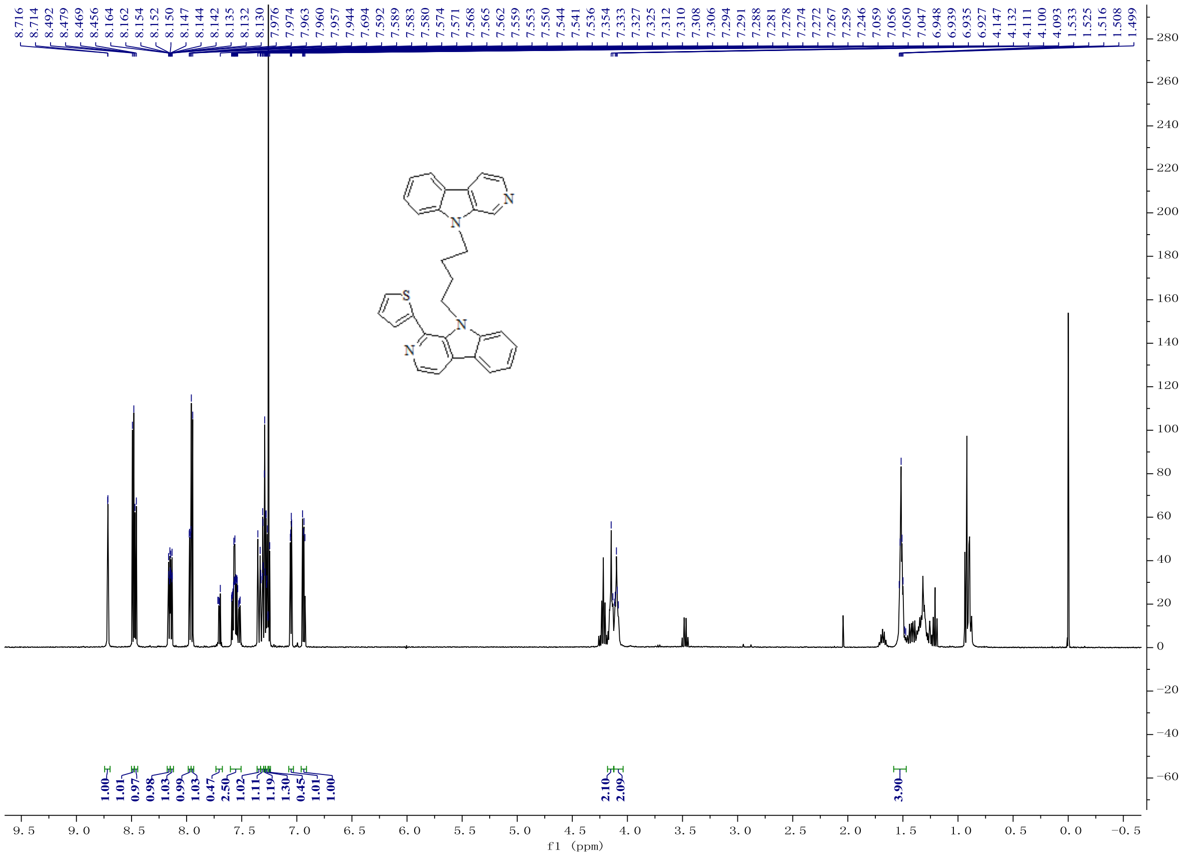

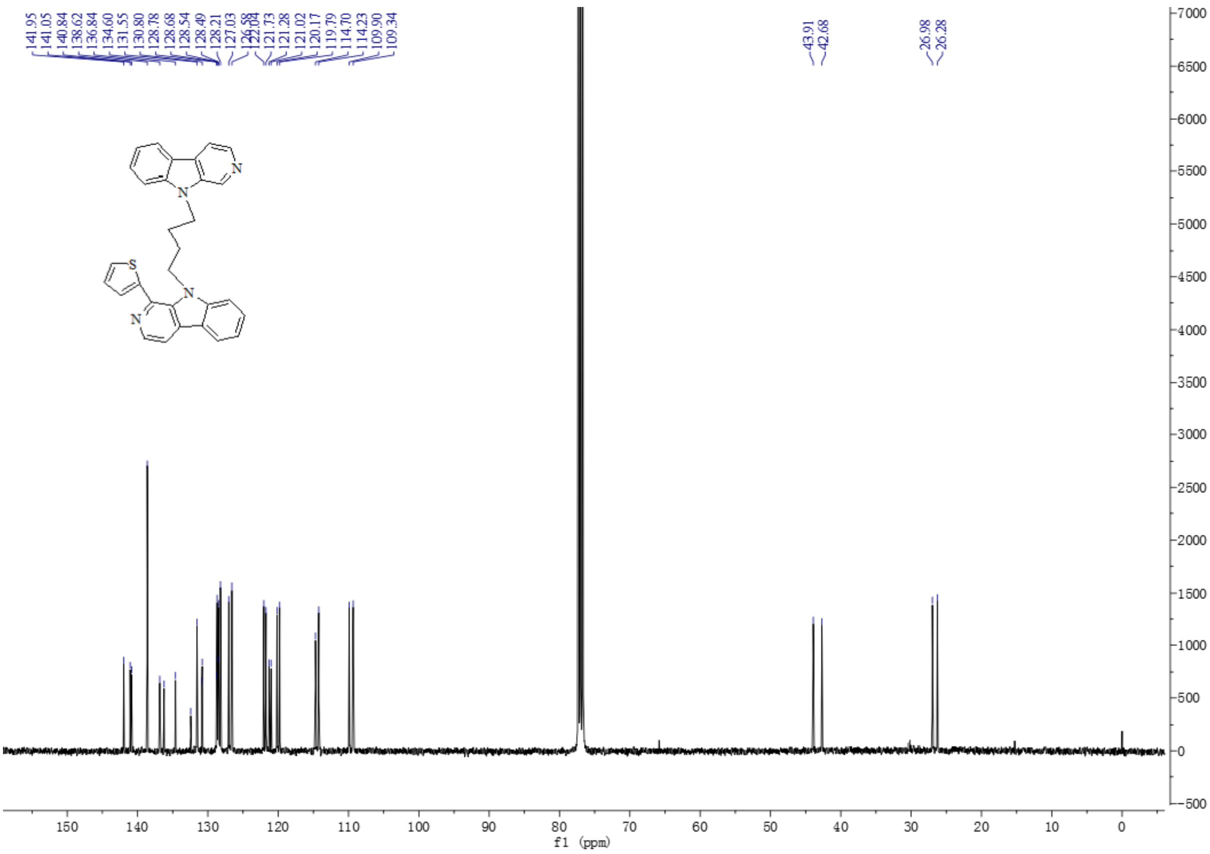

Figure S11 the ^{1}H NMR spectrum and ^{13}C NMR spectrum of compound **5e**

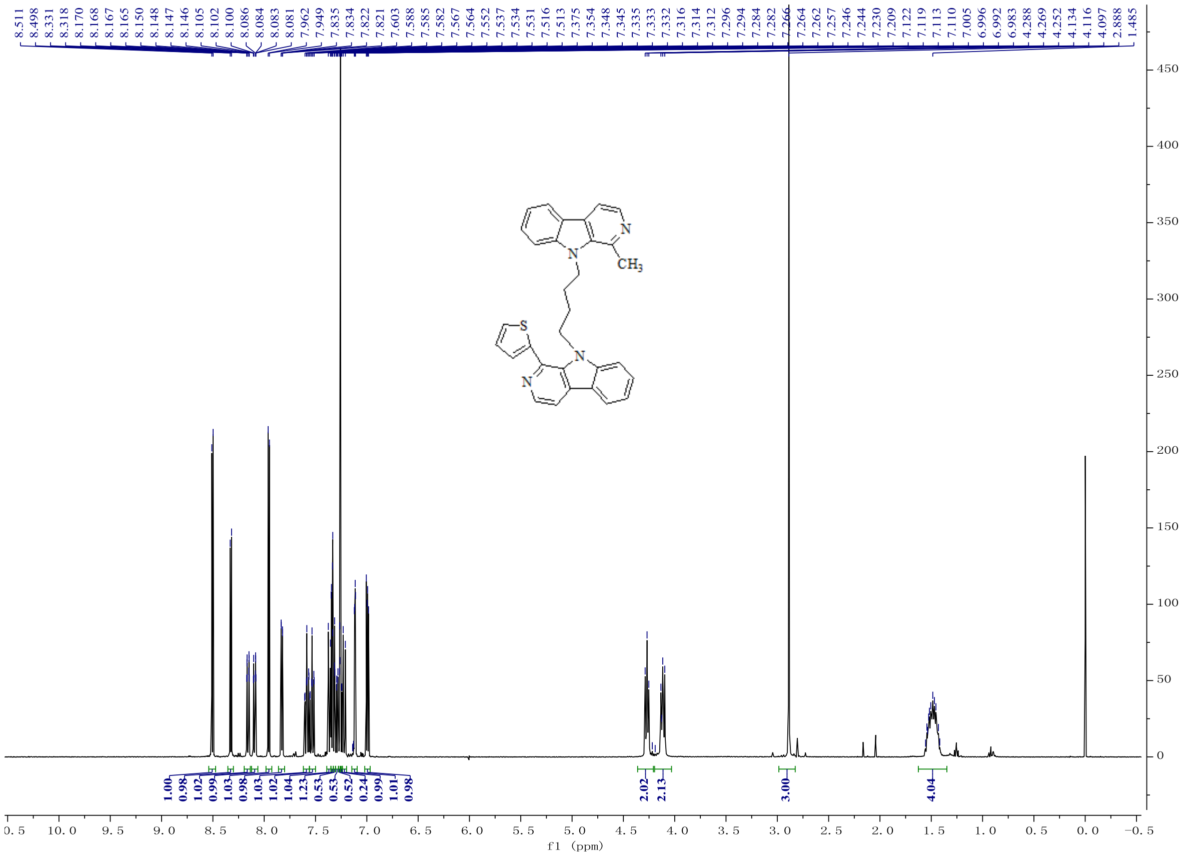

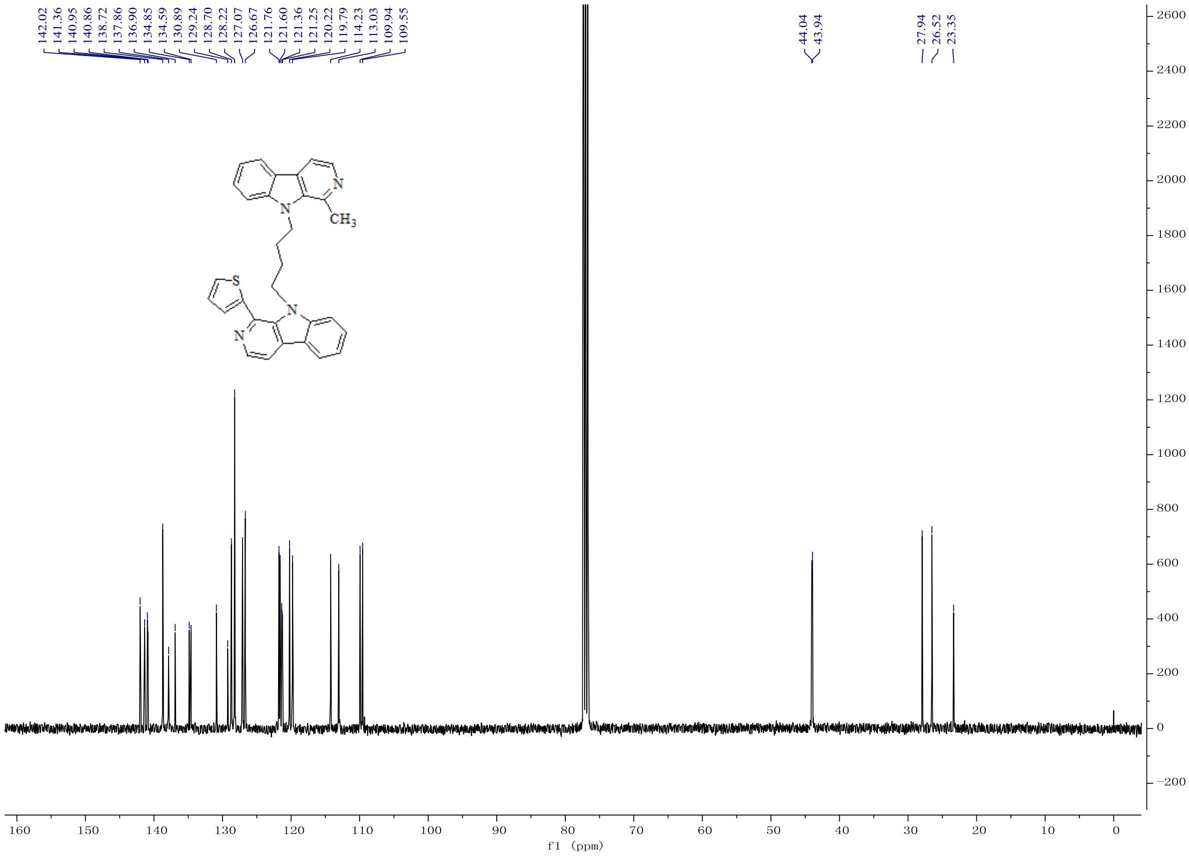

Figure S12 the ^{1}H NMR spectrum and ^{13}C NMR spectrum of compound **5f**

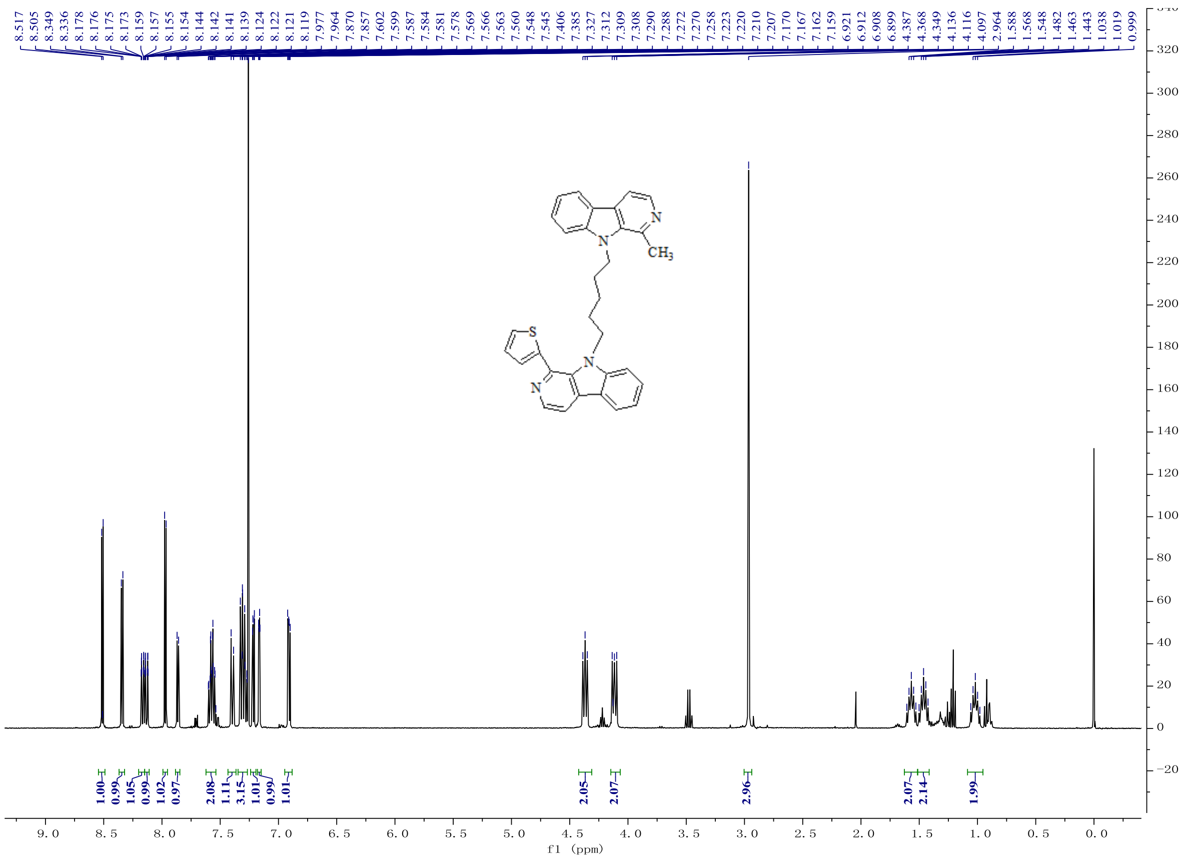

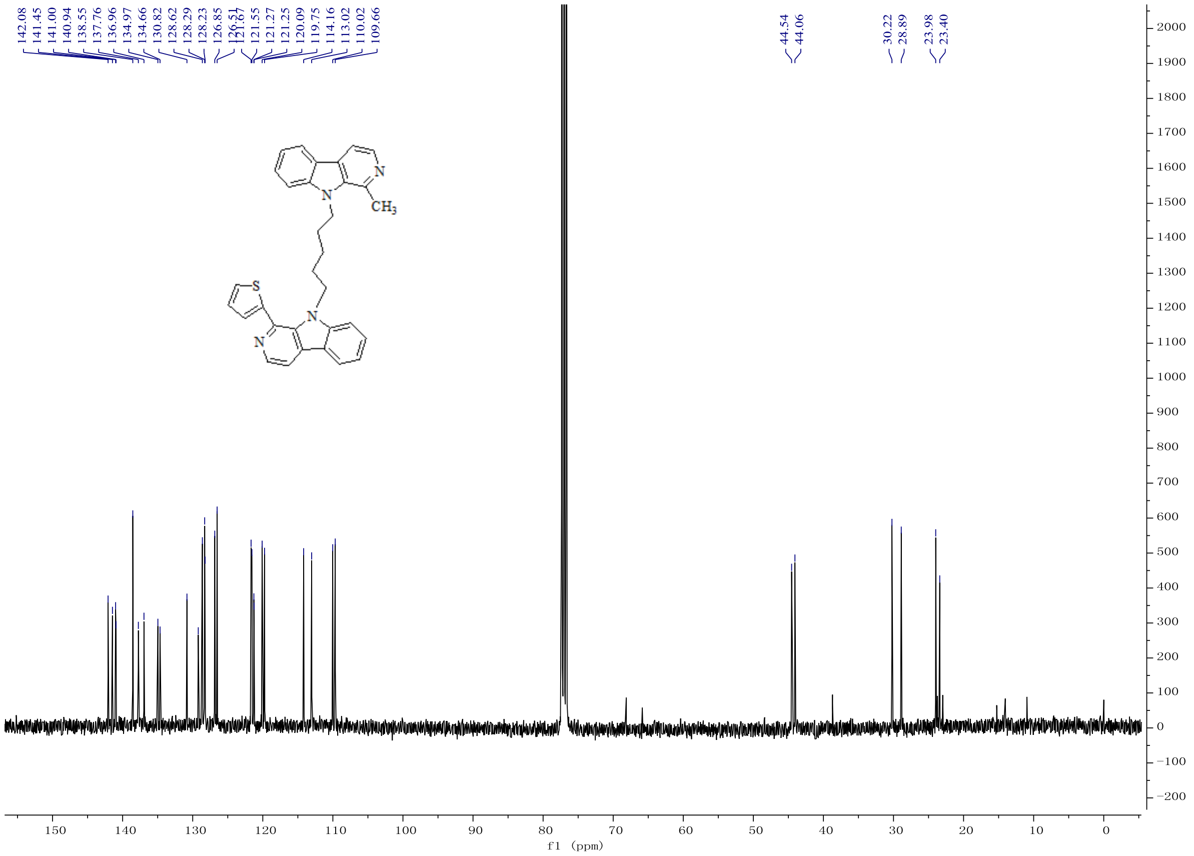

Figure S13 the ^{1}H NMR spectrum and ^{13}C NMR spectrum of compound **5g**

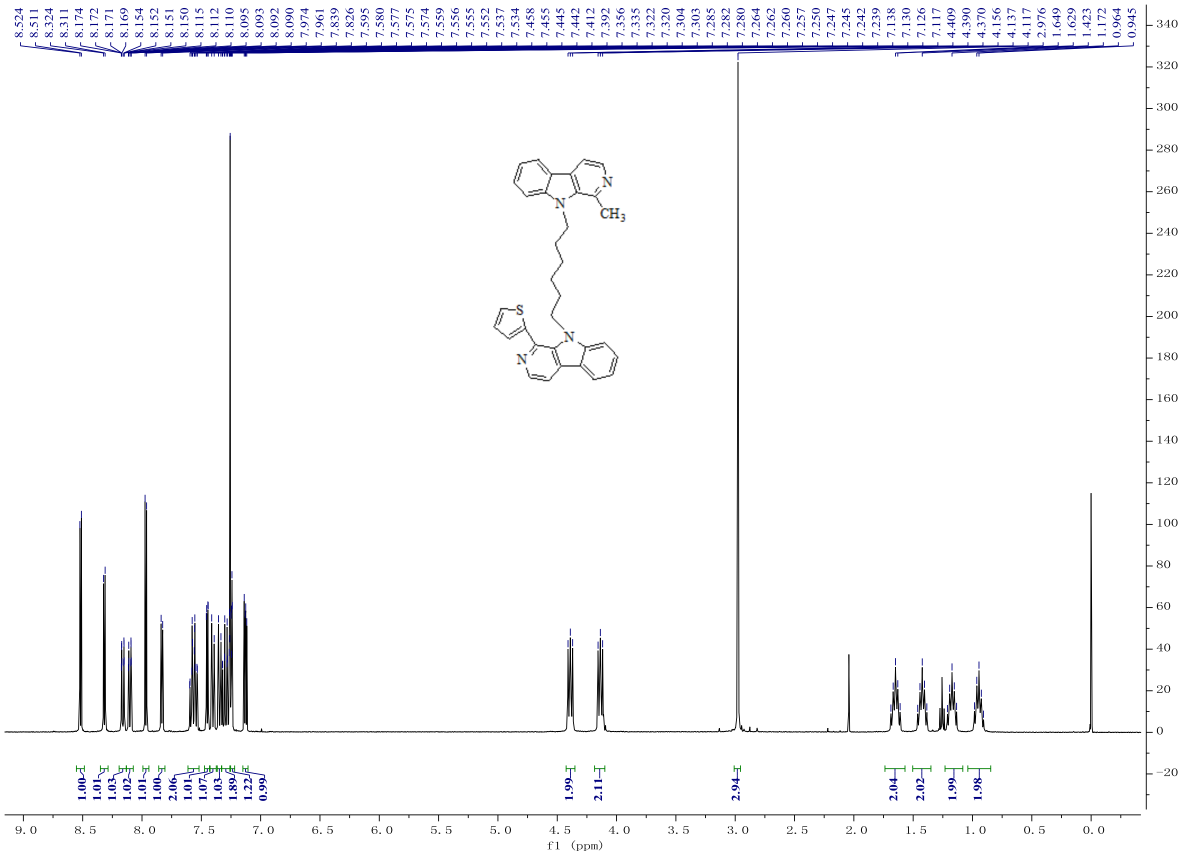

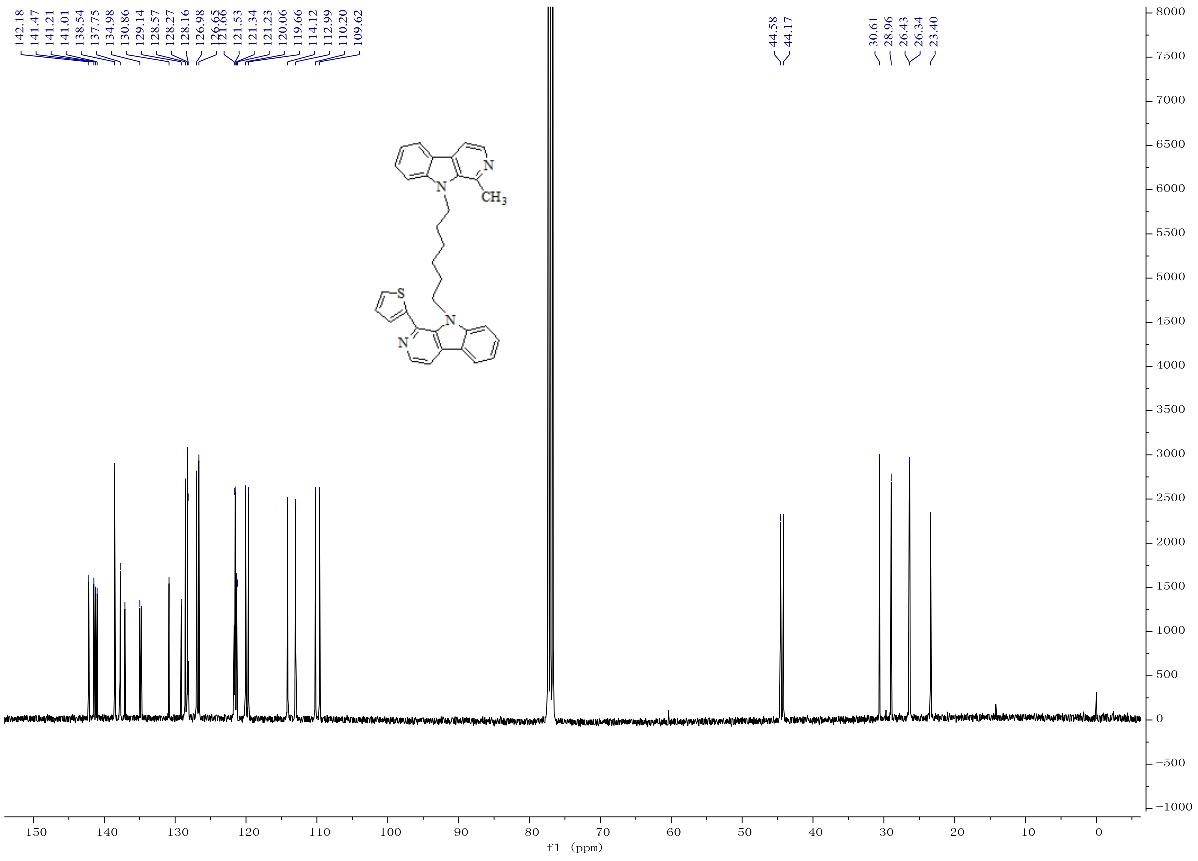

Figure S14 the ^{1}H NMR spectrum and ^{13}C NMR spectrum of compound **5h**

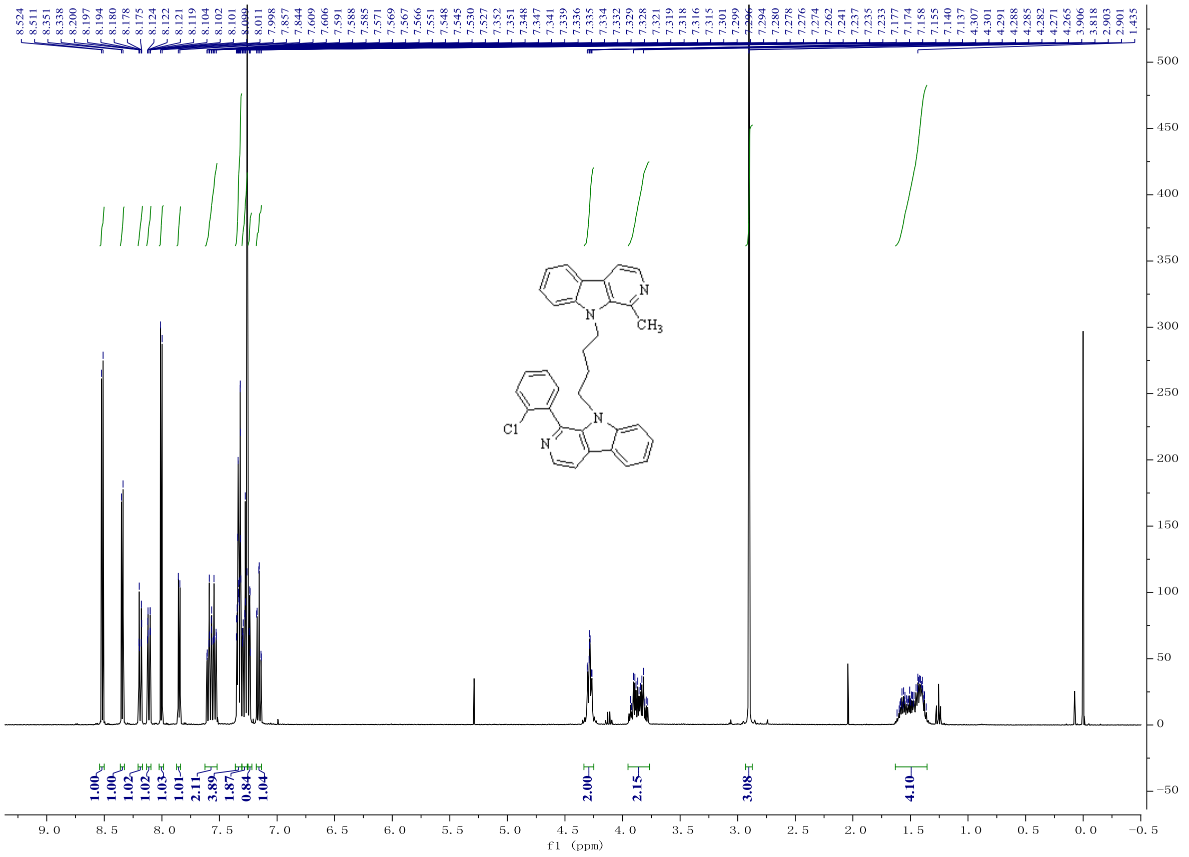

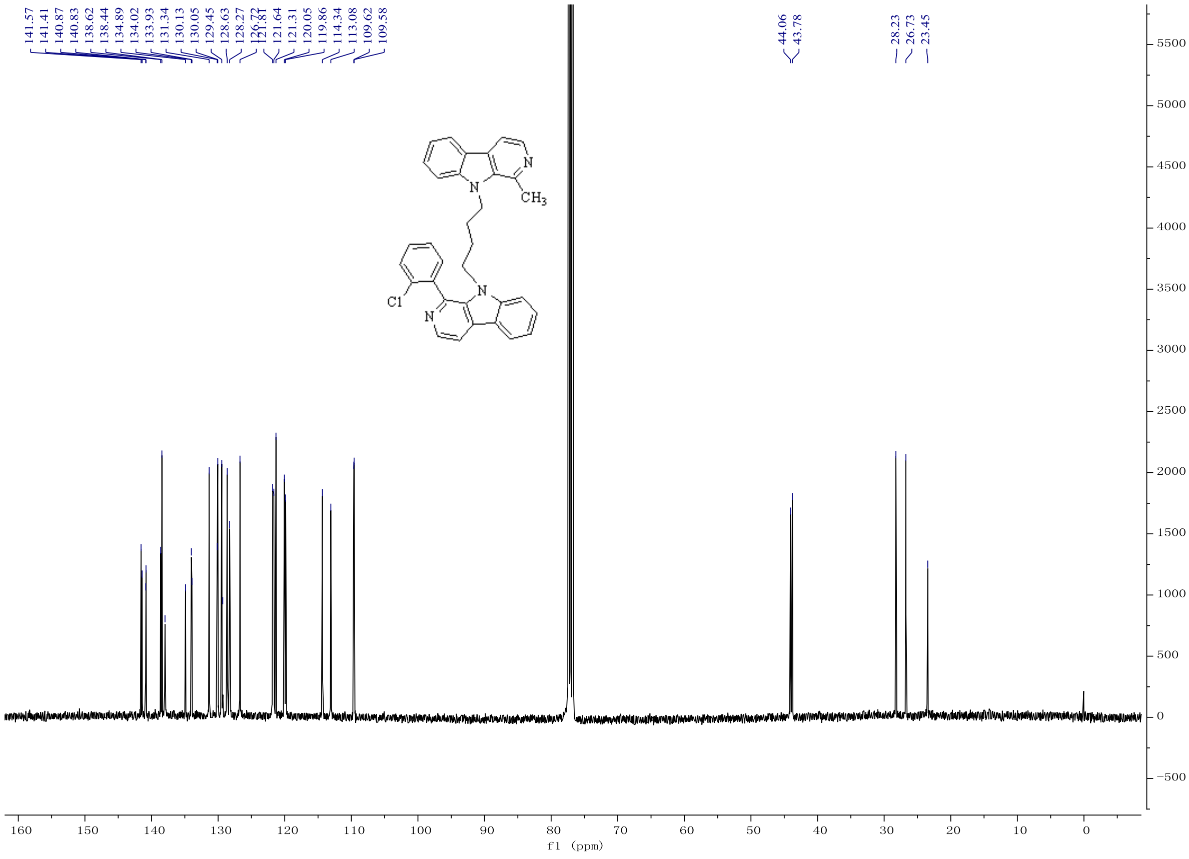

Figure S15 the ^{1}H NMR spectrum and ^{13}C NMR spectrum of compound **5i**

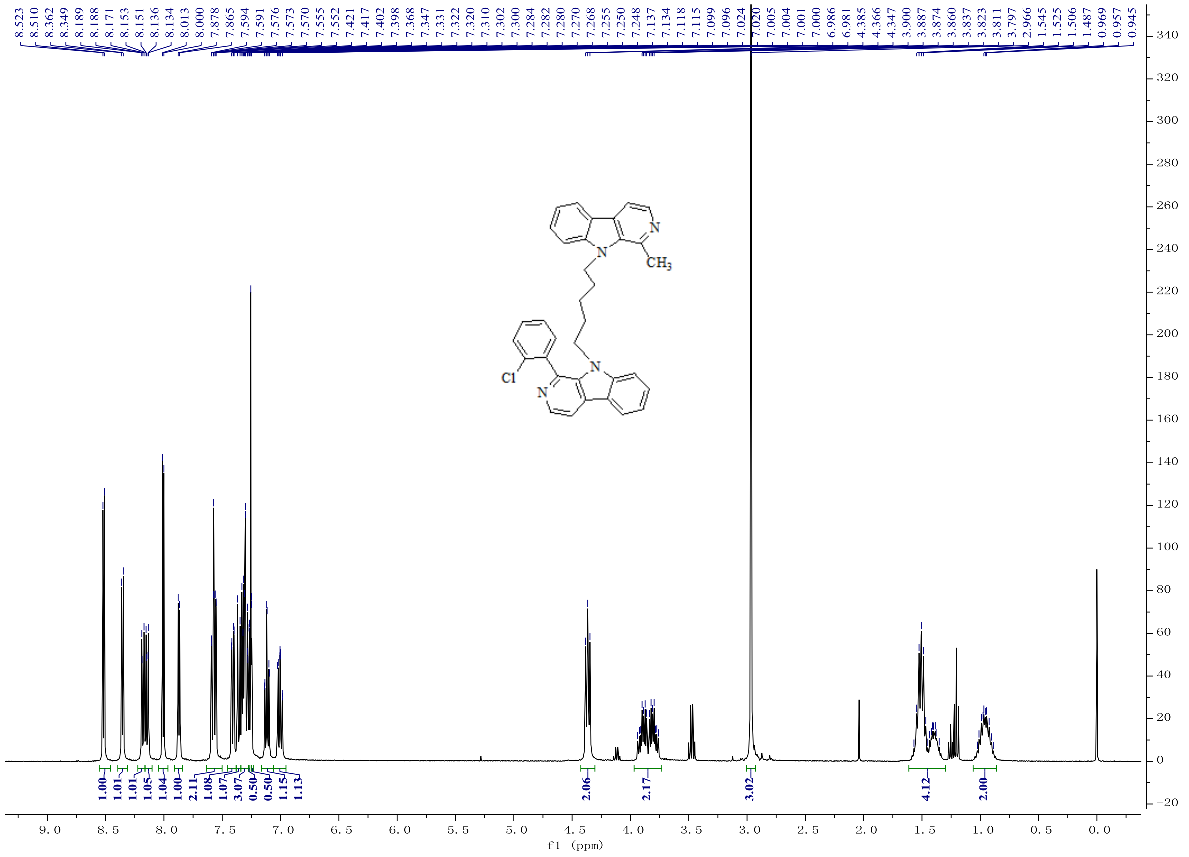

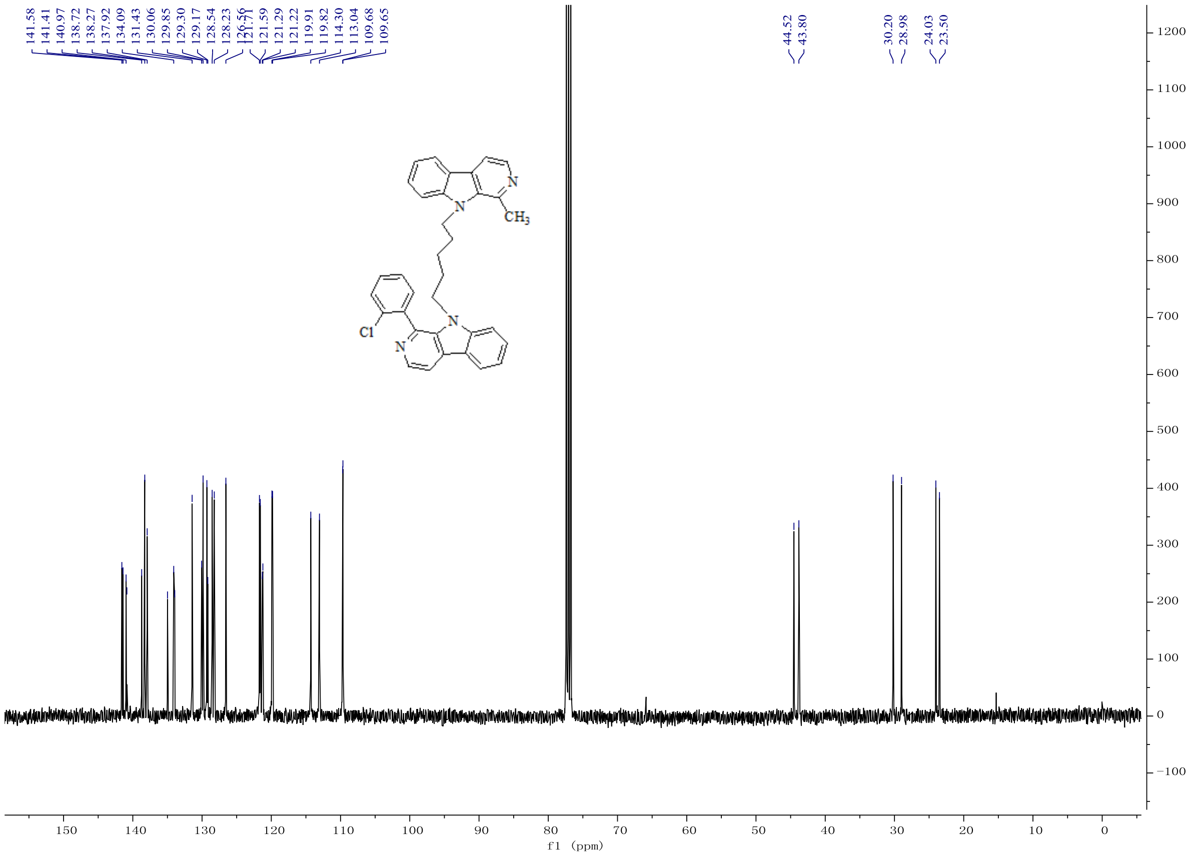

Figure S16 the ^{1}H NMR spectrum and ^{13}C NMR spectrum of compound **5j**

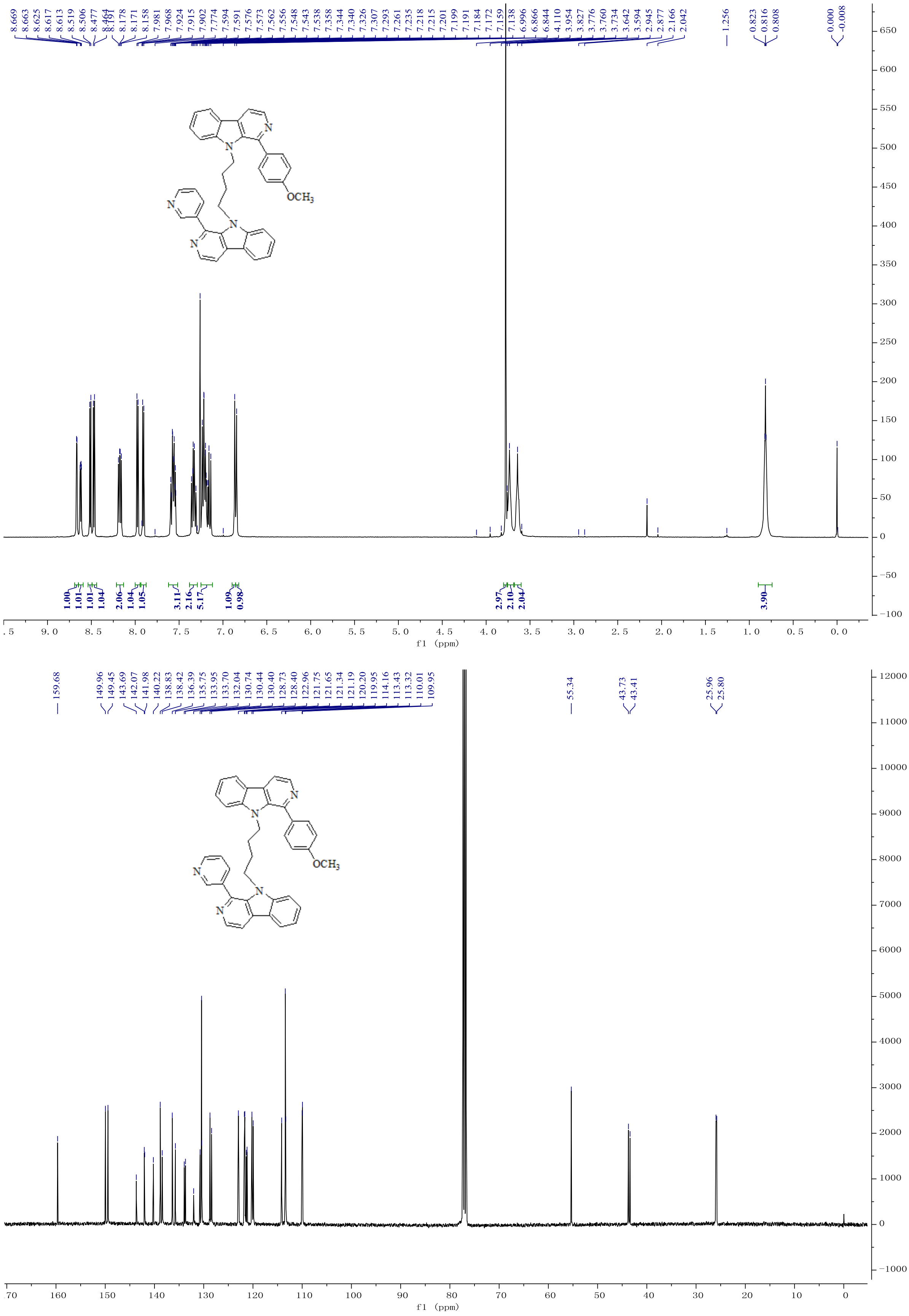

Figure S17 the ^{1}H NMR spectrum and ^{13}C NMR spectrum of compound **5k**

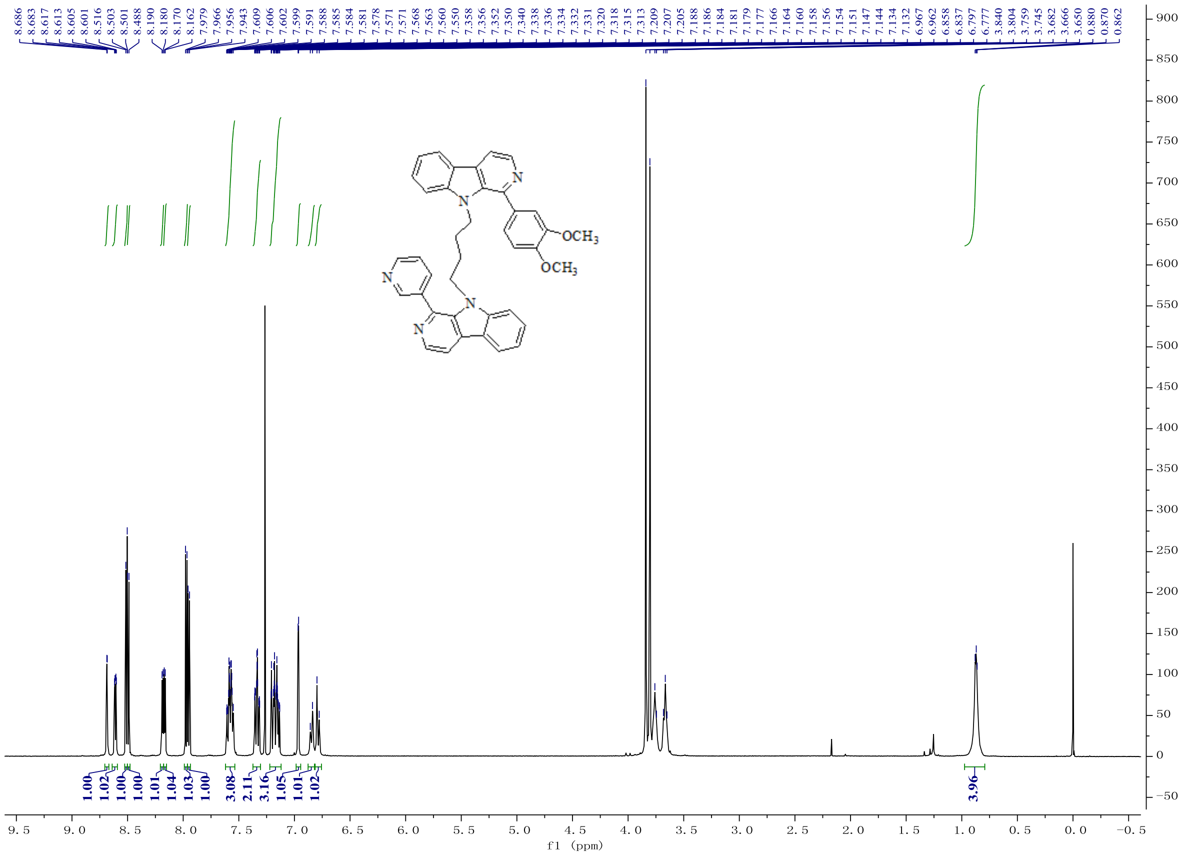

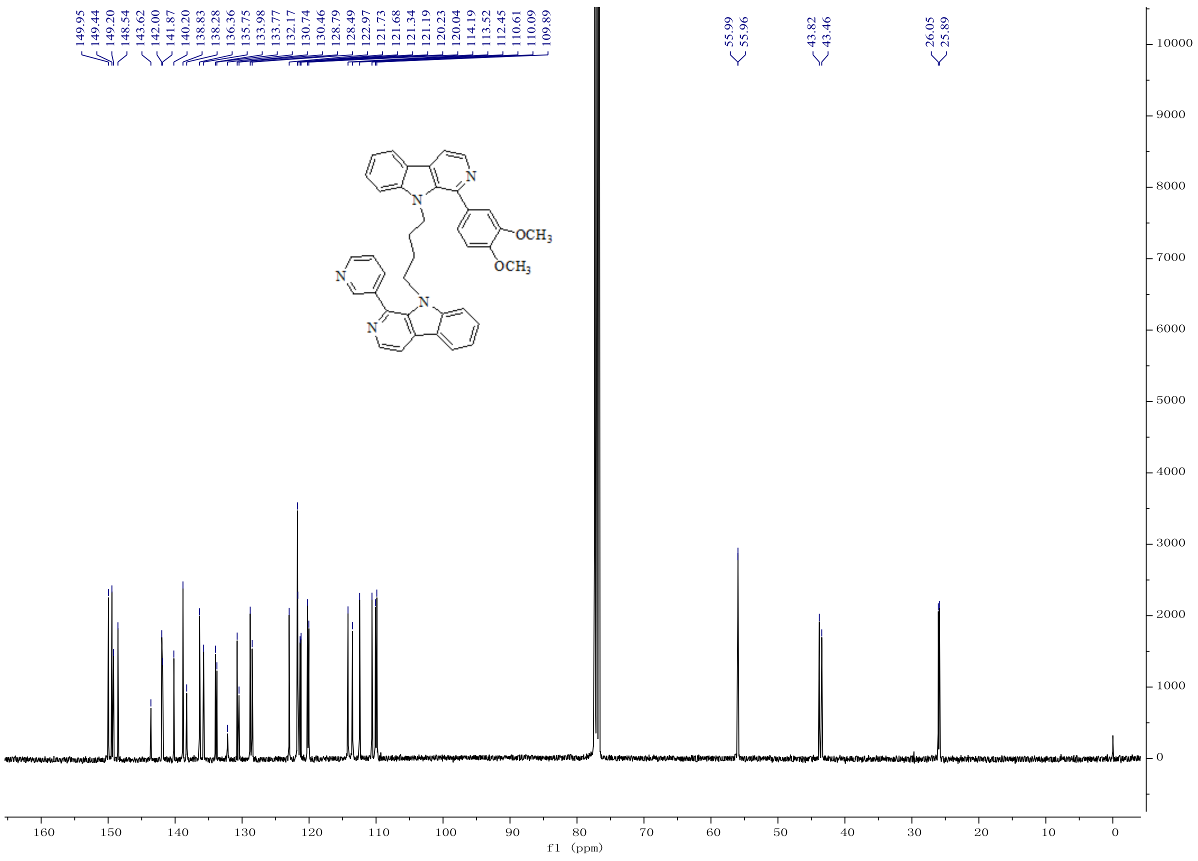

Figure S18 the ^{1}H NMR spectrum and ^{13}C NMR spectrum of compound **5l**

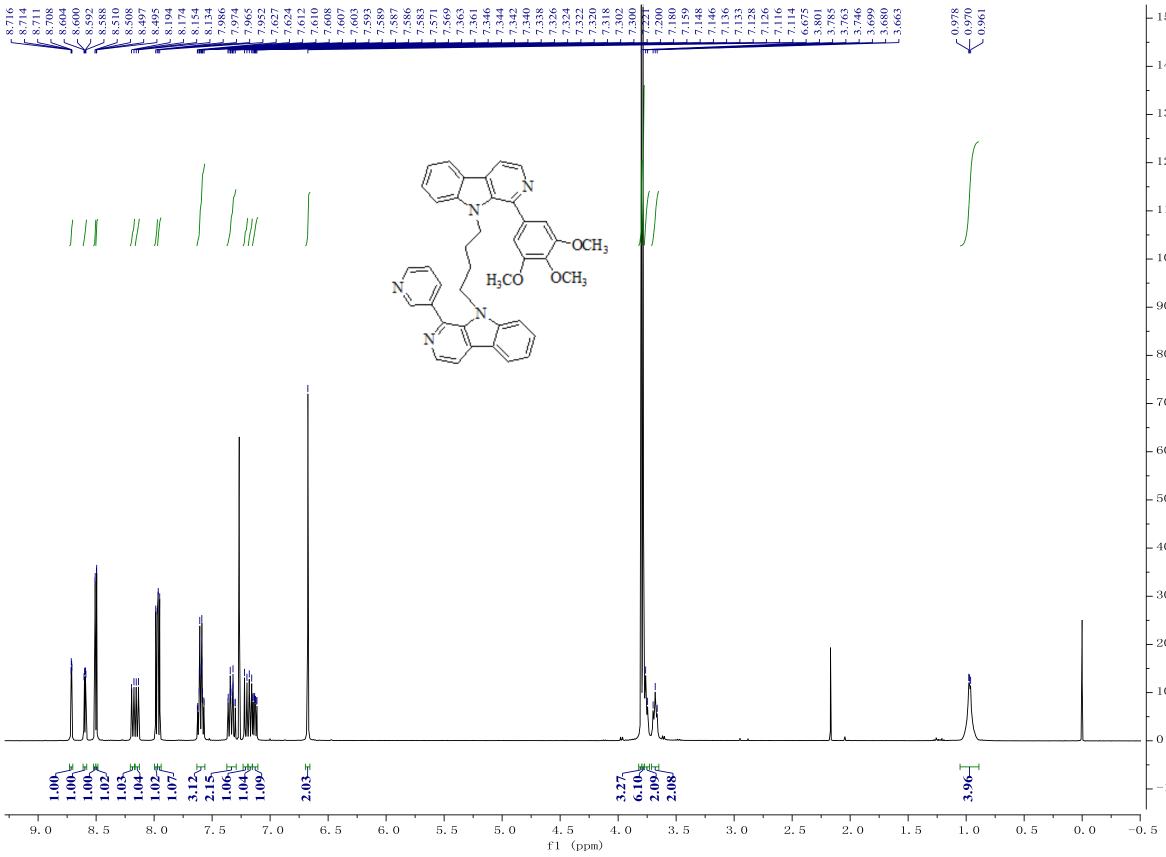

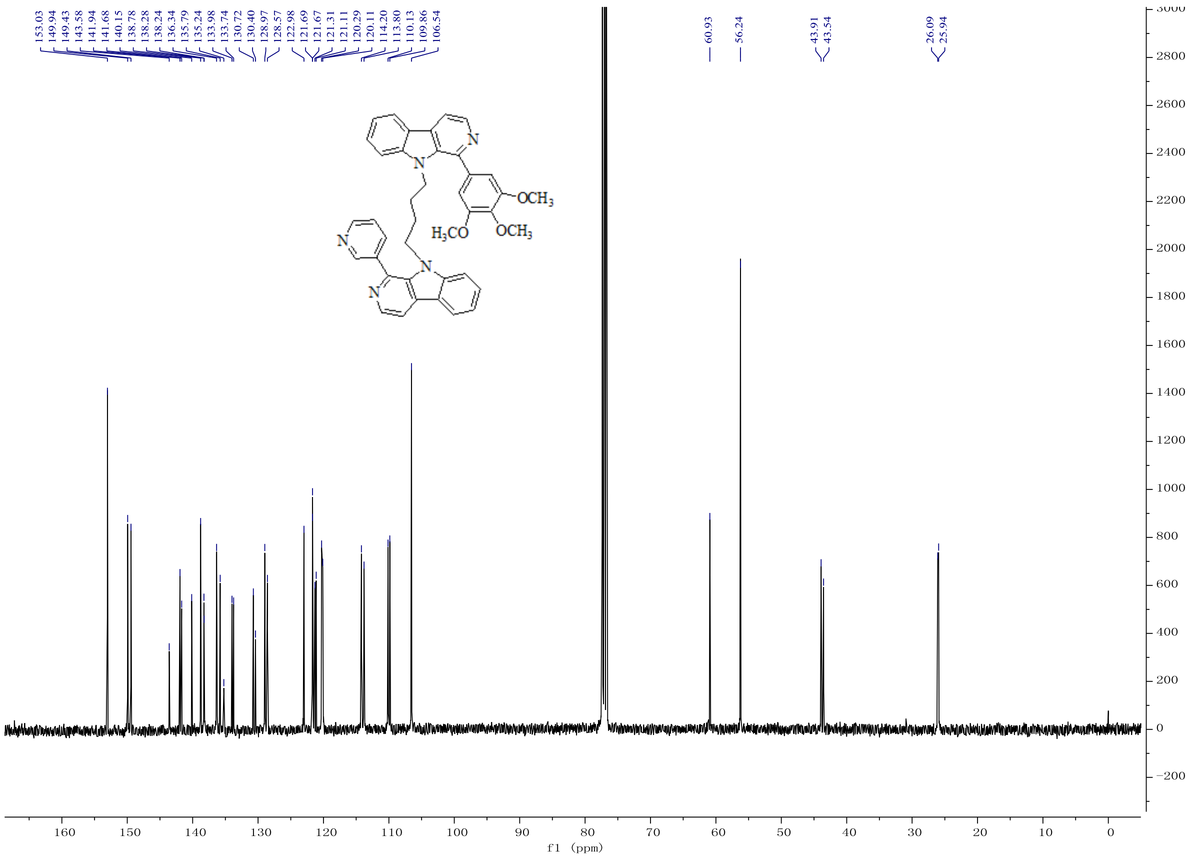

Figure S19 the ^{1}H NMR spectrum and ^{13}C NMR spectrum of compound **5m**

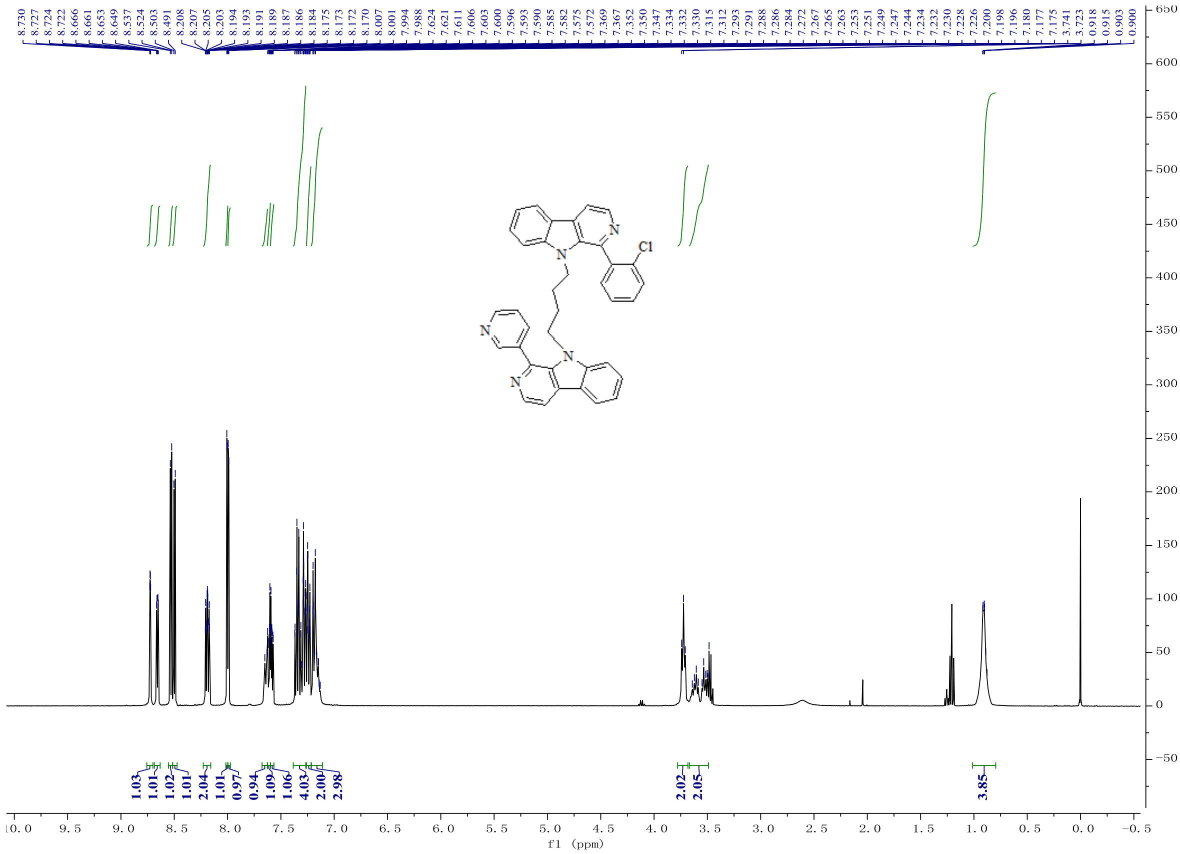

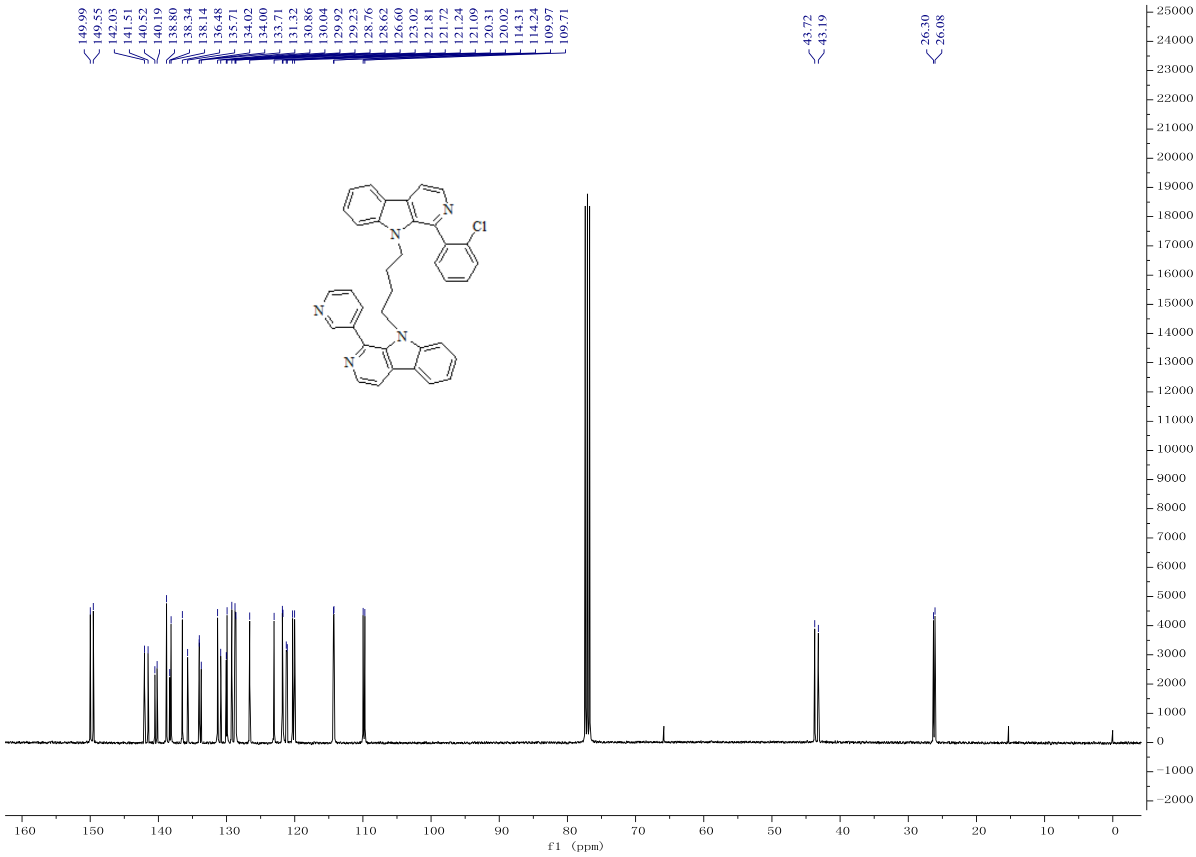

Figure S20 the ^{1}H NMR spectrum and ^{13}C NMR spectrum of compound **5n**

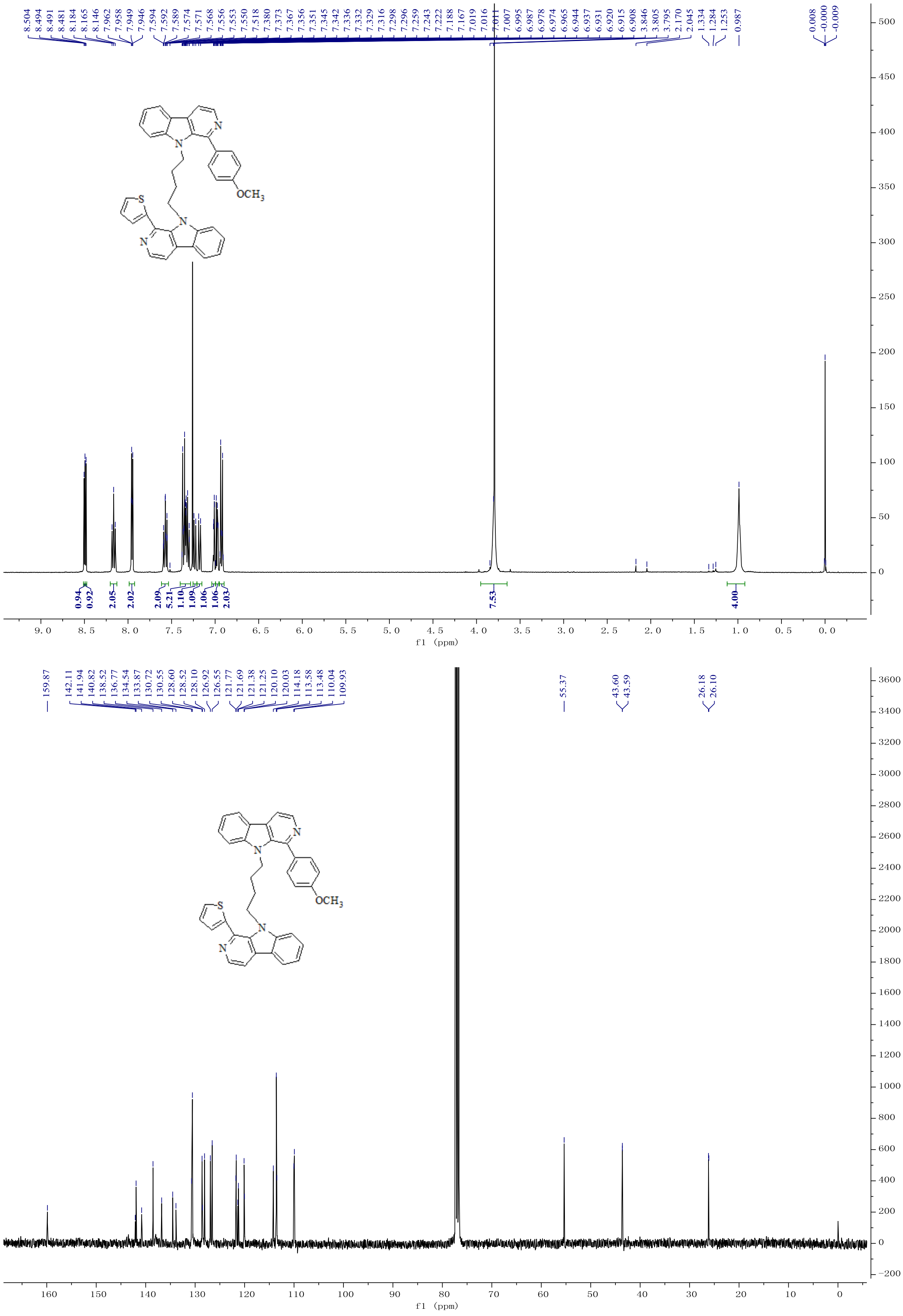

Figure S21 the ^{1}H NMR spectrum and ^{13}C NMR spectrum of compound **5o**

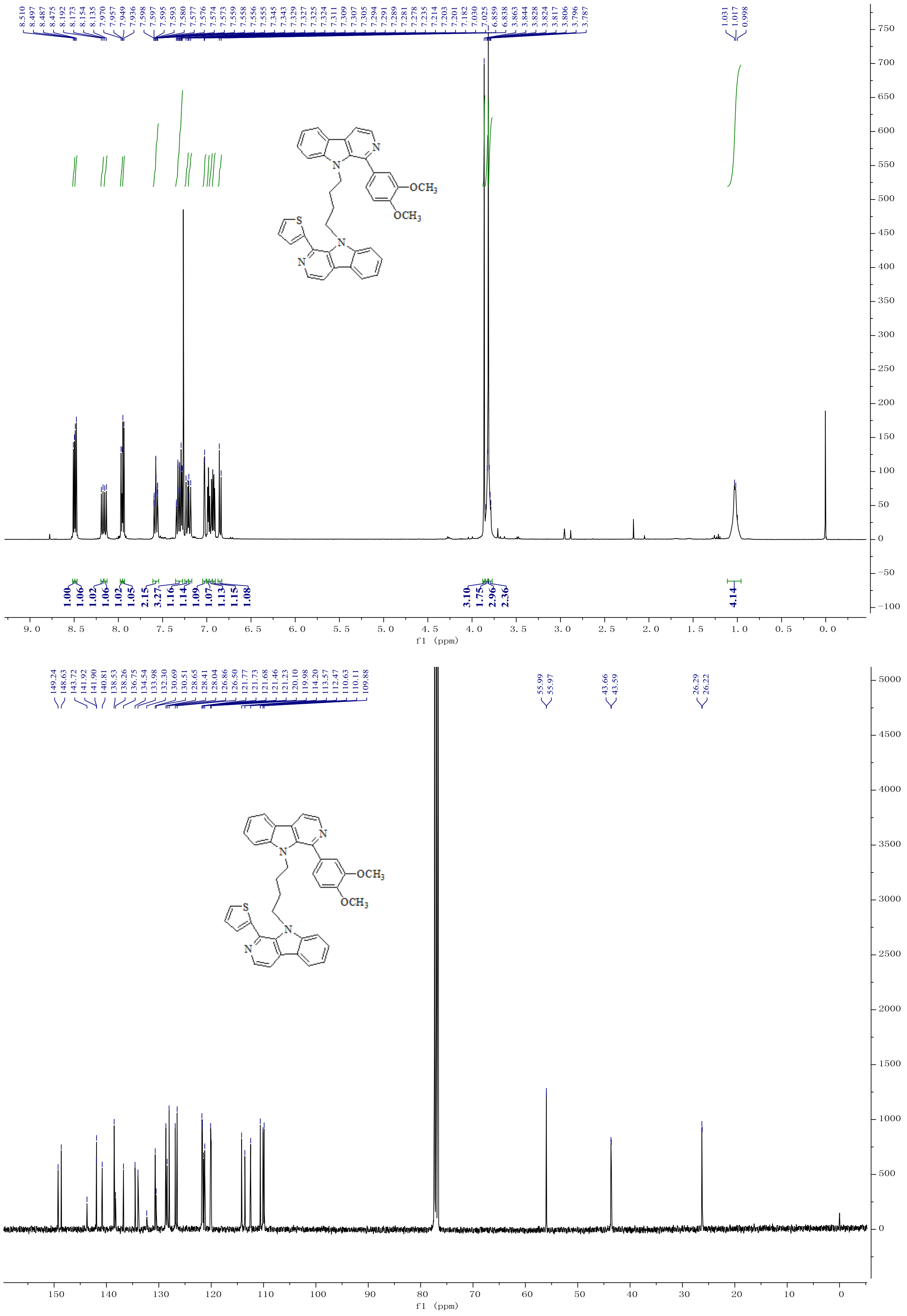

Figure S22 the ^{1}H NMR spectrum and ^{13}C NMR spectrum of compound **5p**

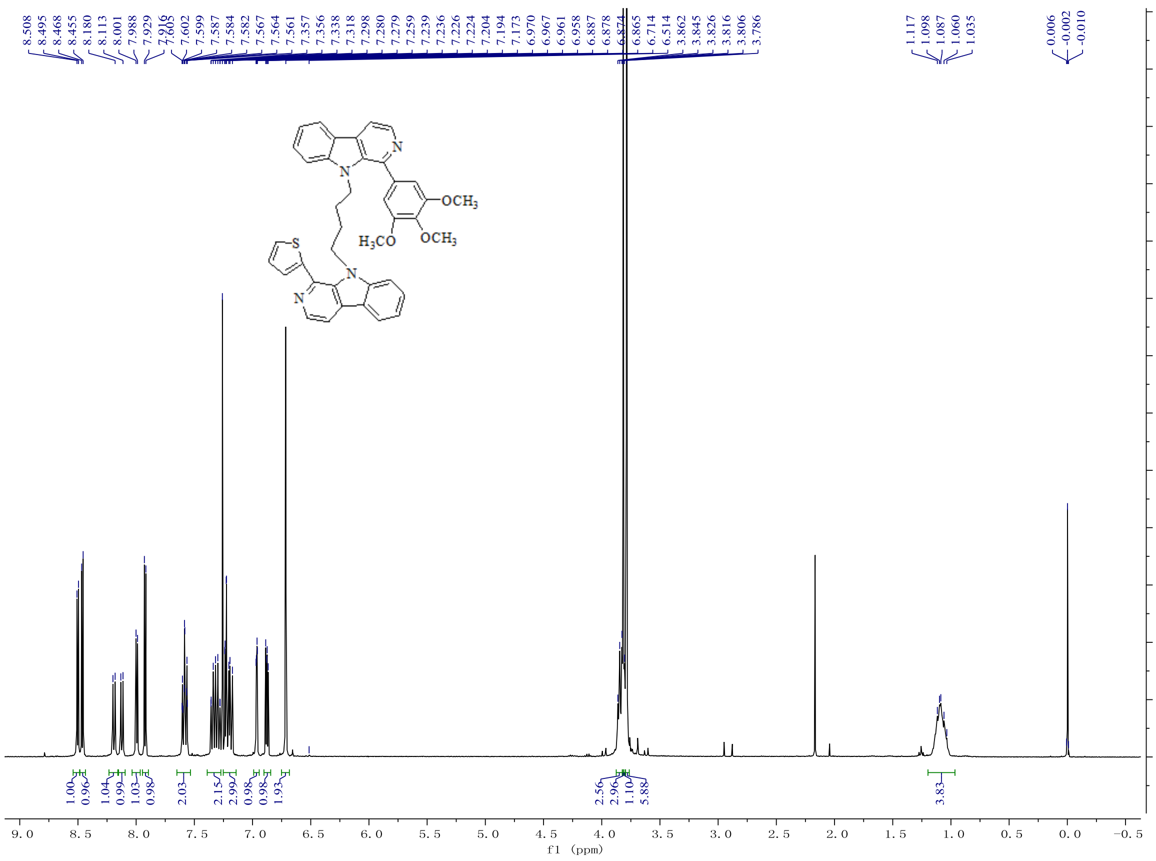

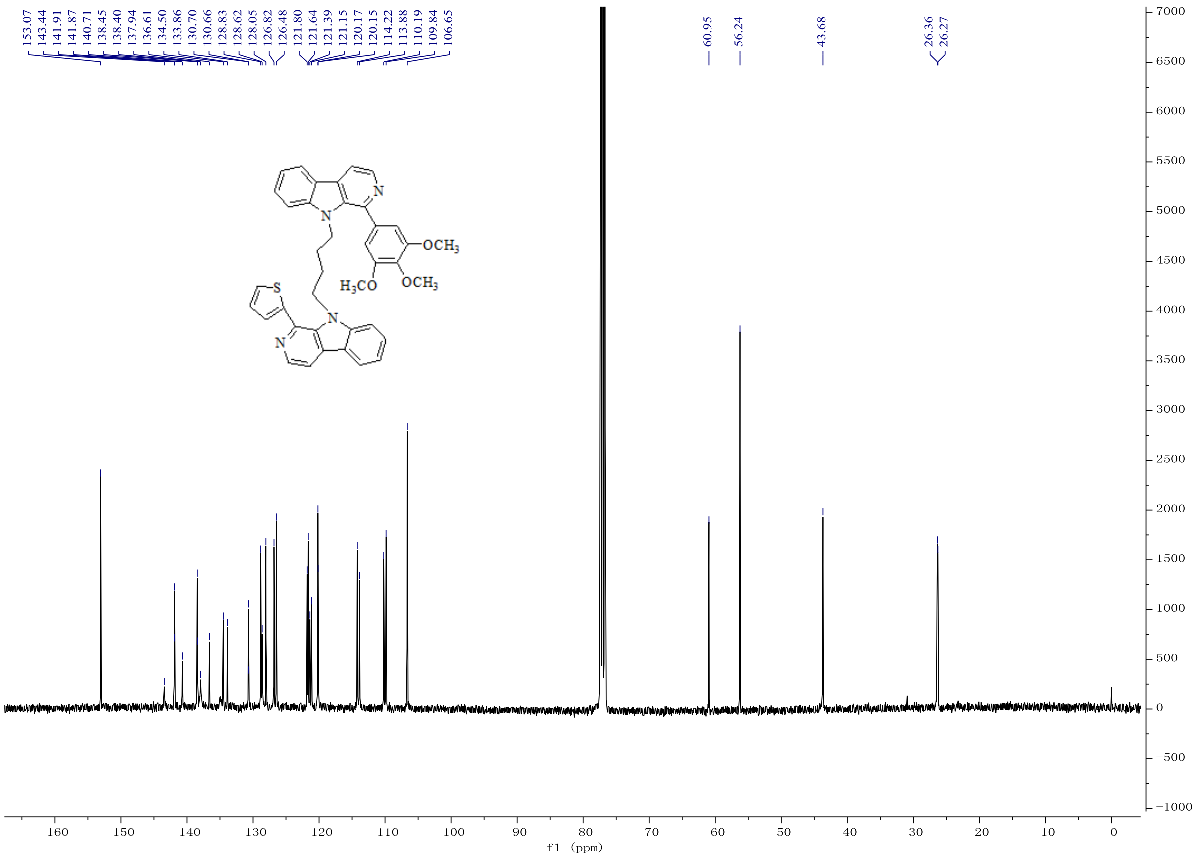

Figure S23 the ^{1}H NMR spectrum and ^{13}C NMR spectrum of compound **5q**

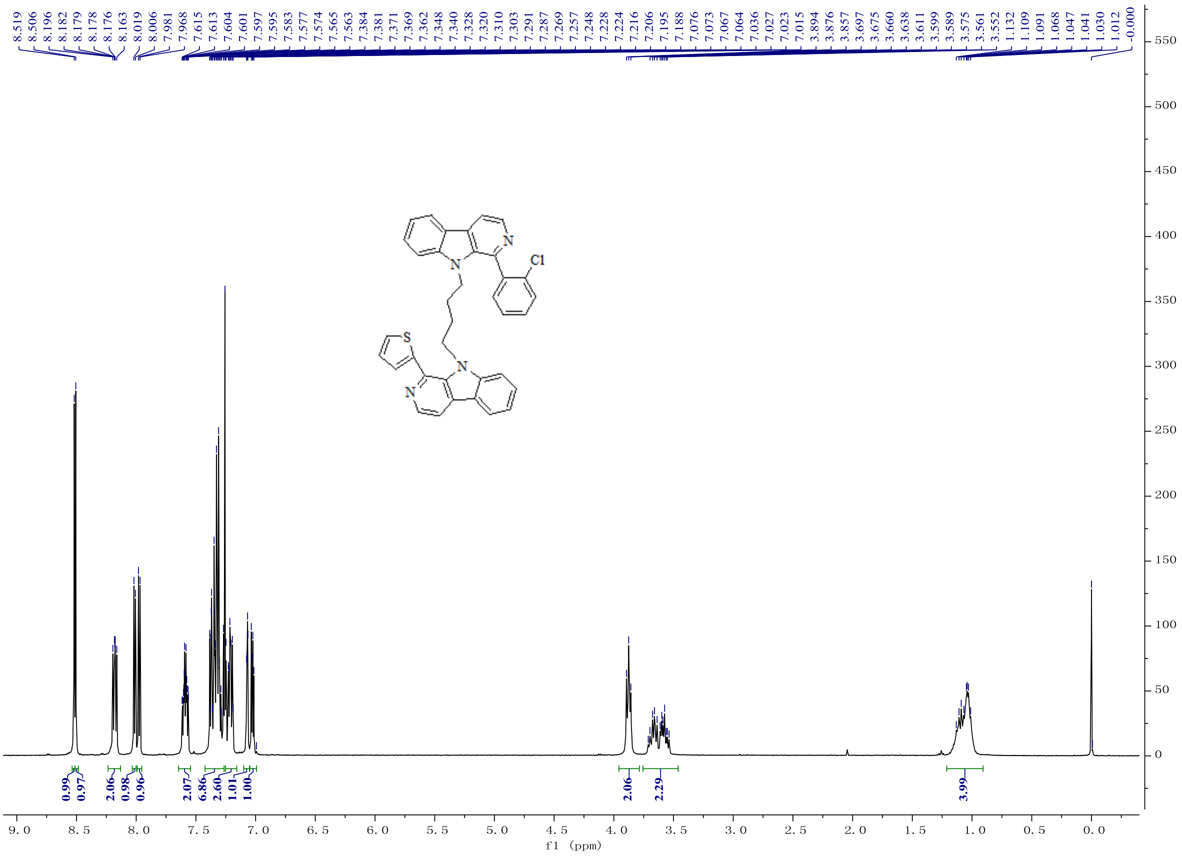

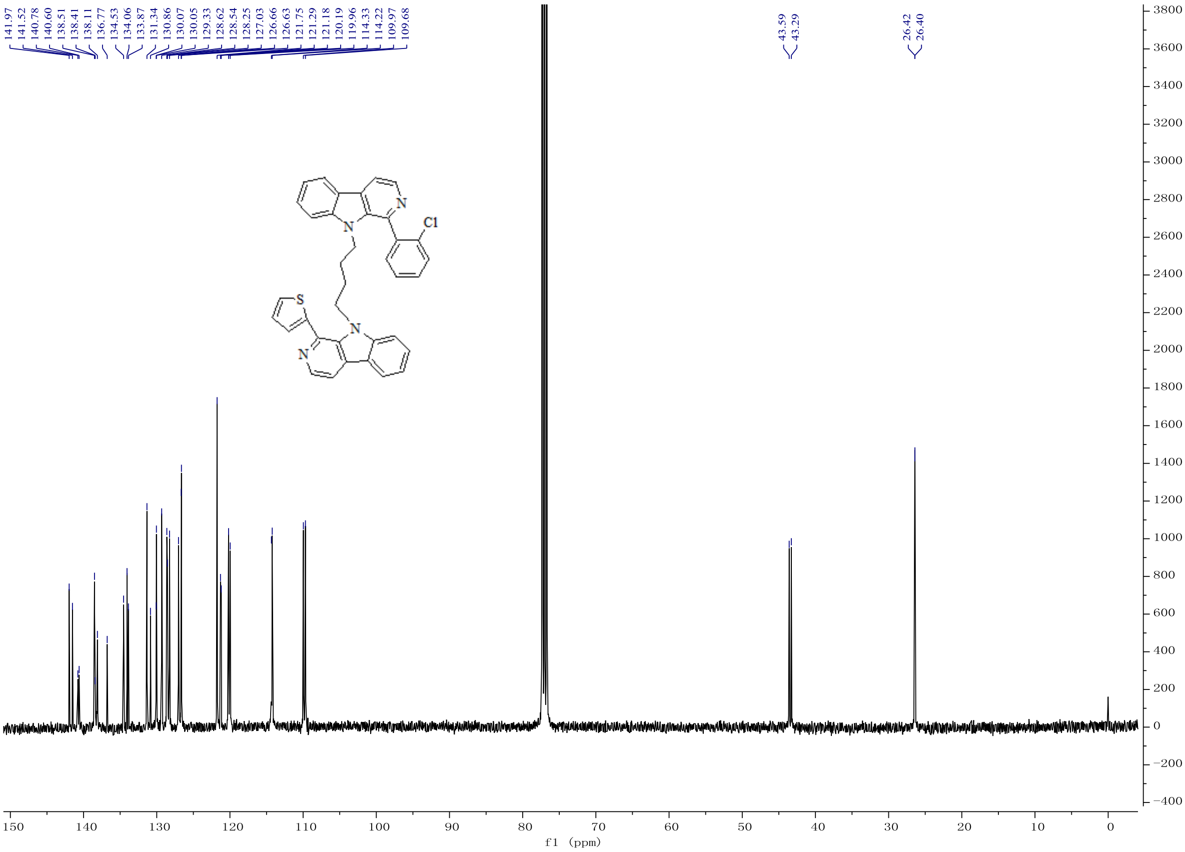

Figure S24 the ^{1}H NMR spectrum and ^{13}C NMR spectrum of compound **5r**

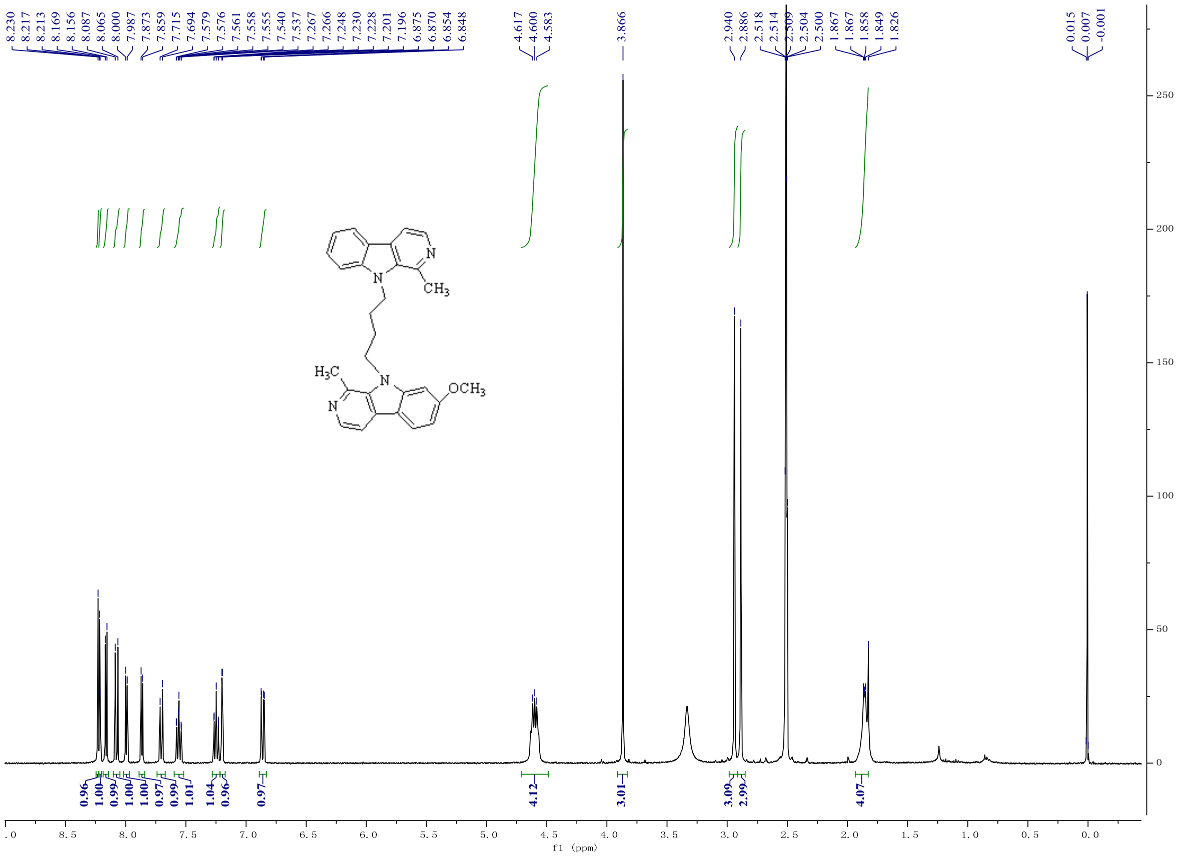

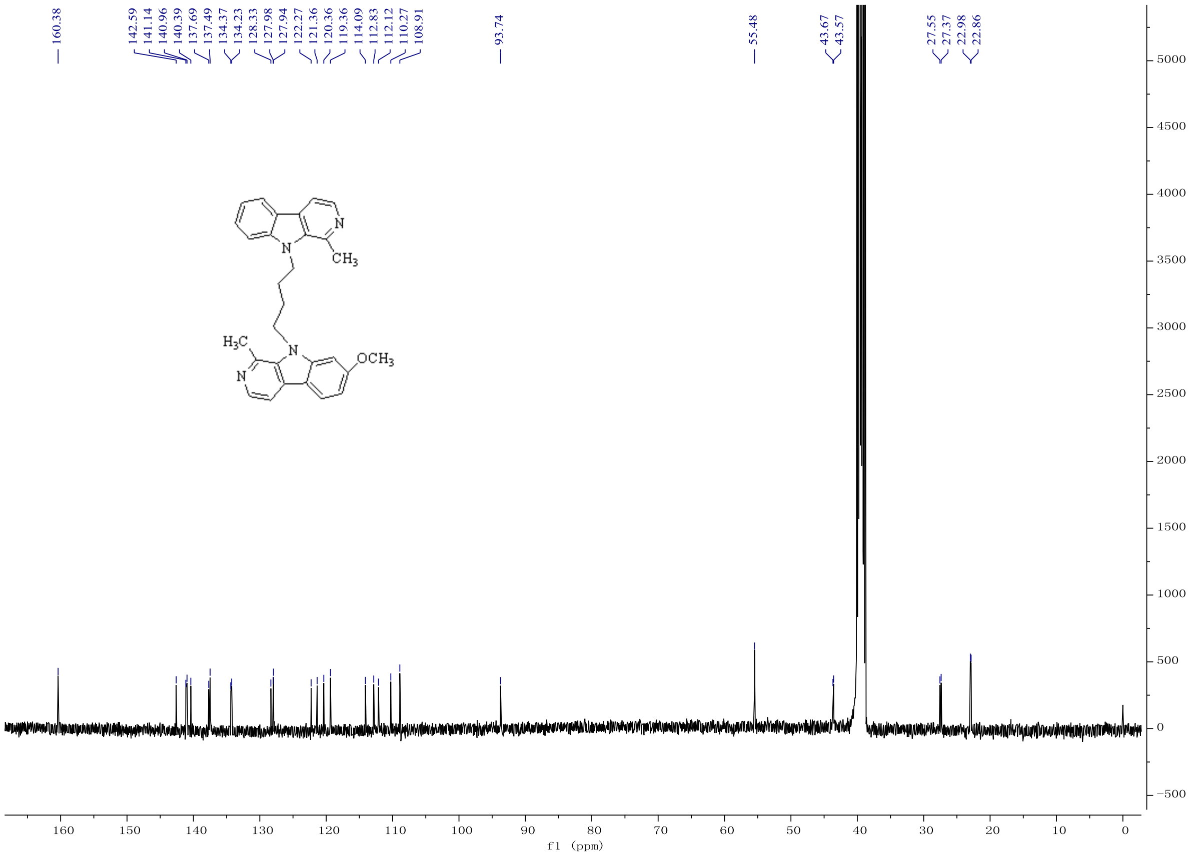

Figure S25 the ^{1}H NMR spectrum and ^{13}C NMR spectrum of compound **5s**

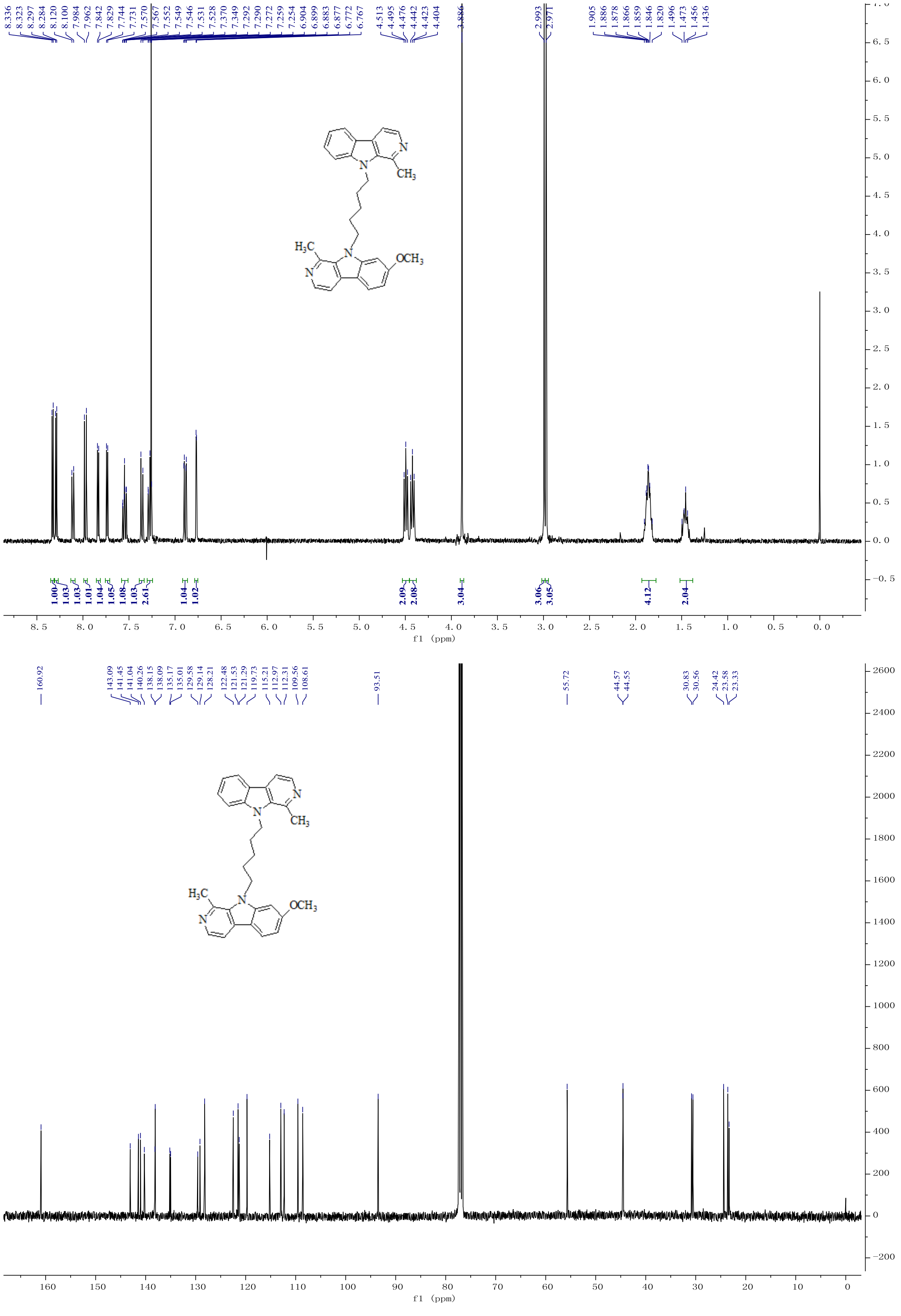

Figure S26 the ^{1}H NMR spectrum and ^{13}C NMR spectrum of compound **5t**

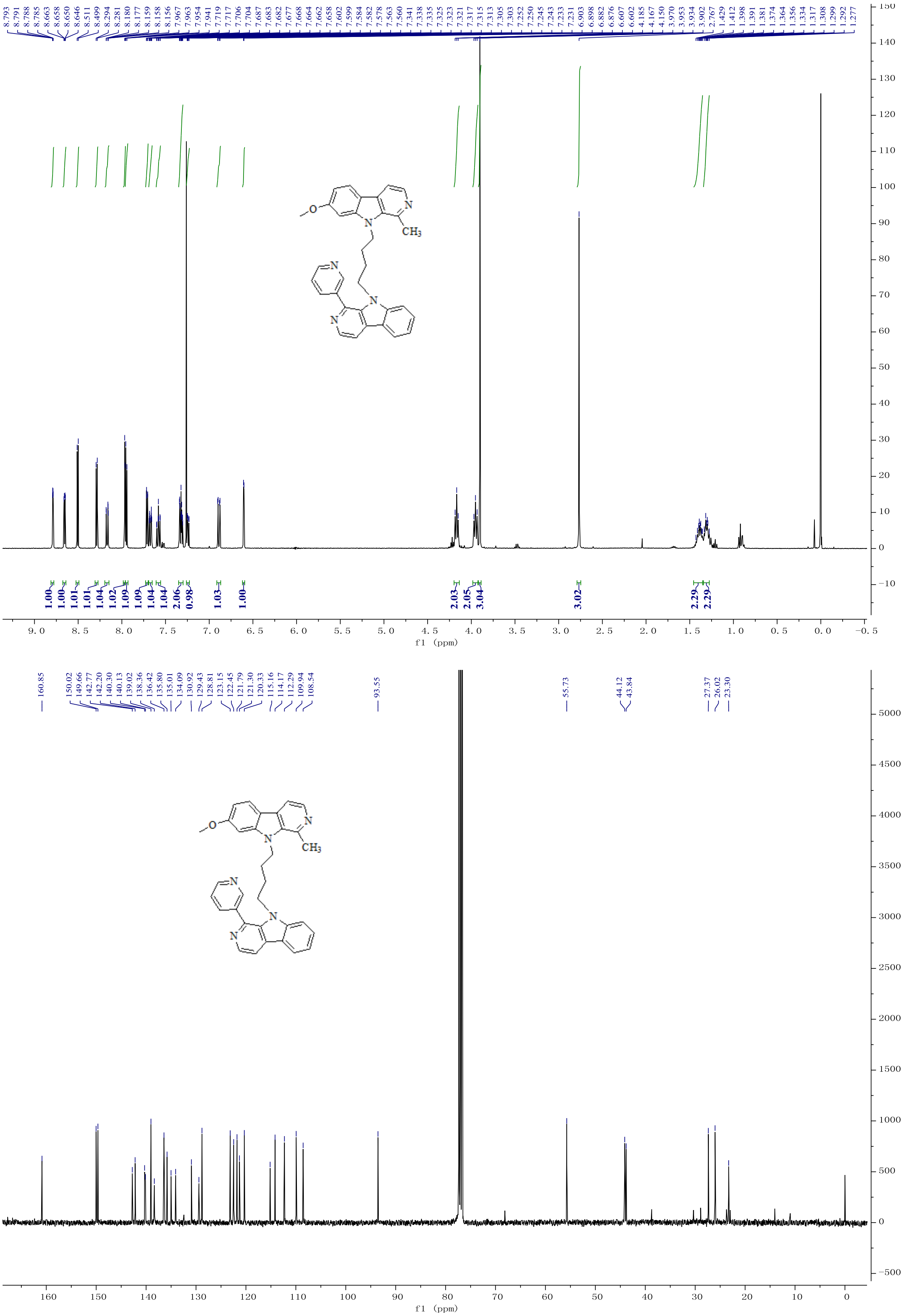

Figure S27 the ^{1}H NMR spectrum and ^{13}C NMR spectrum of compound **5u**

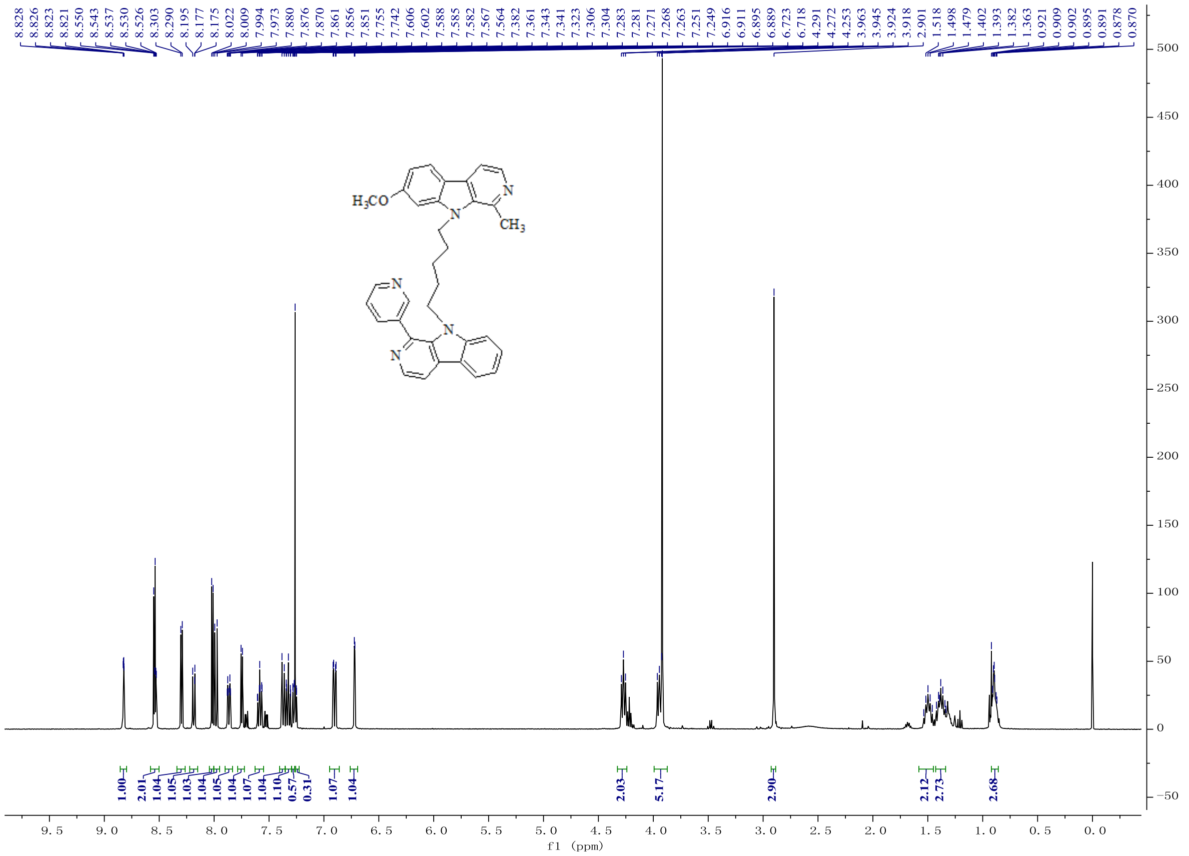

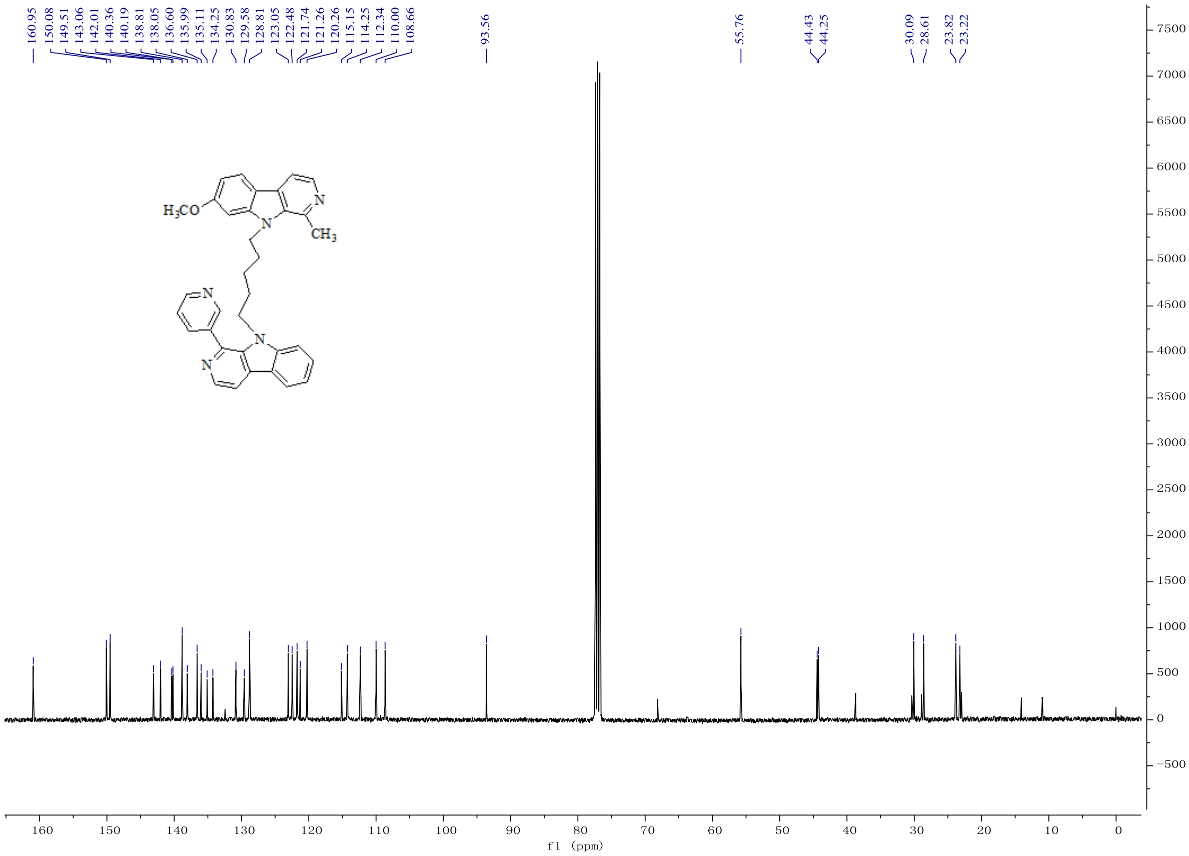

Figure S28 the ^{1}H NMR spectrum and ^{13}C NMR spectrum of compound **5v**

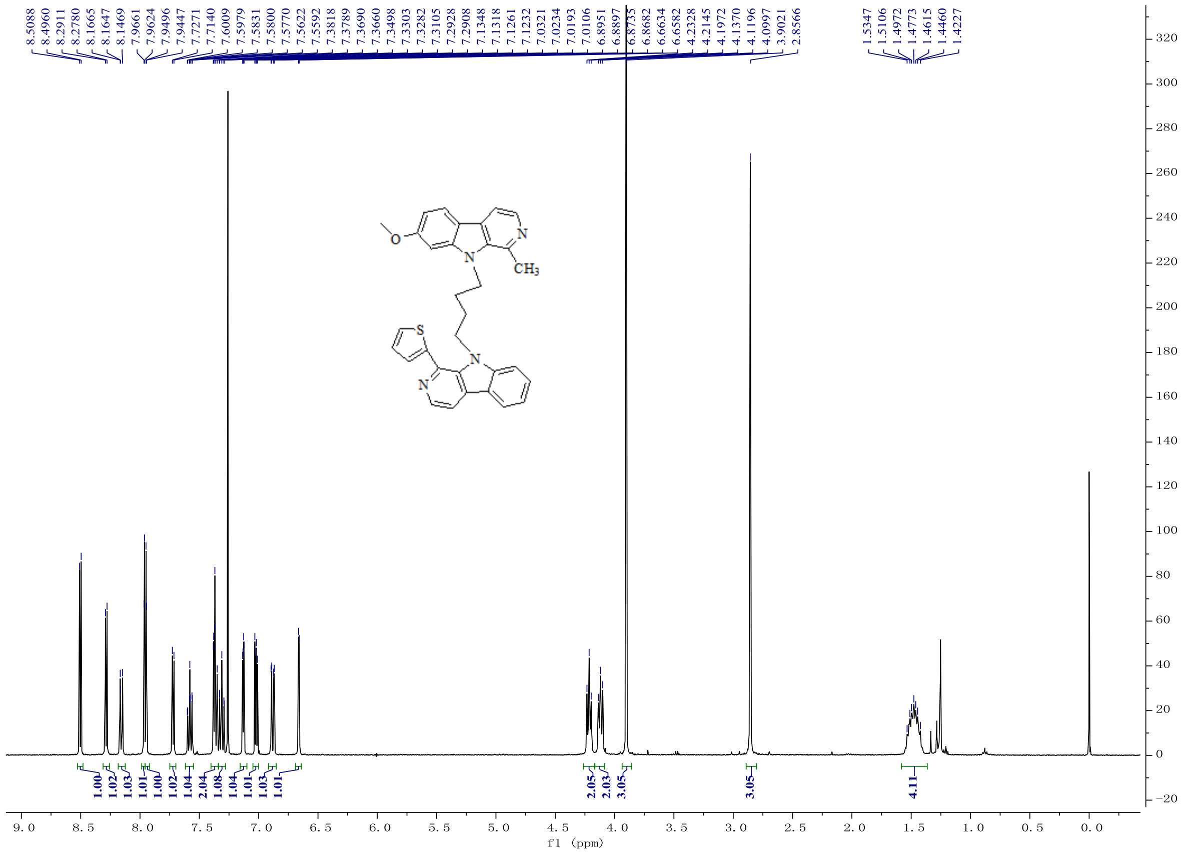

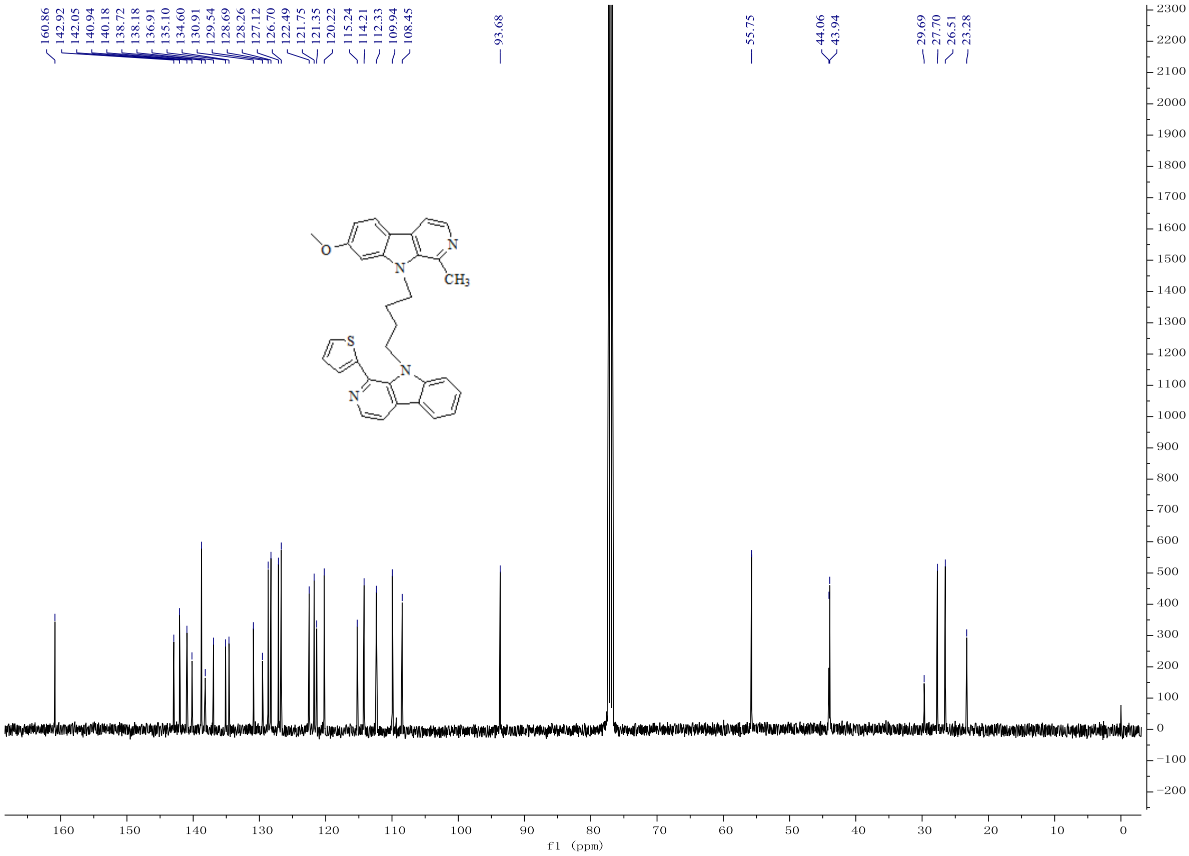

Figure S29 the ^{1}H NMR spectrum and ^{13}C NMR spectrum of compound **5w**

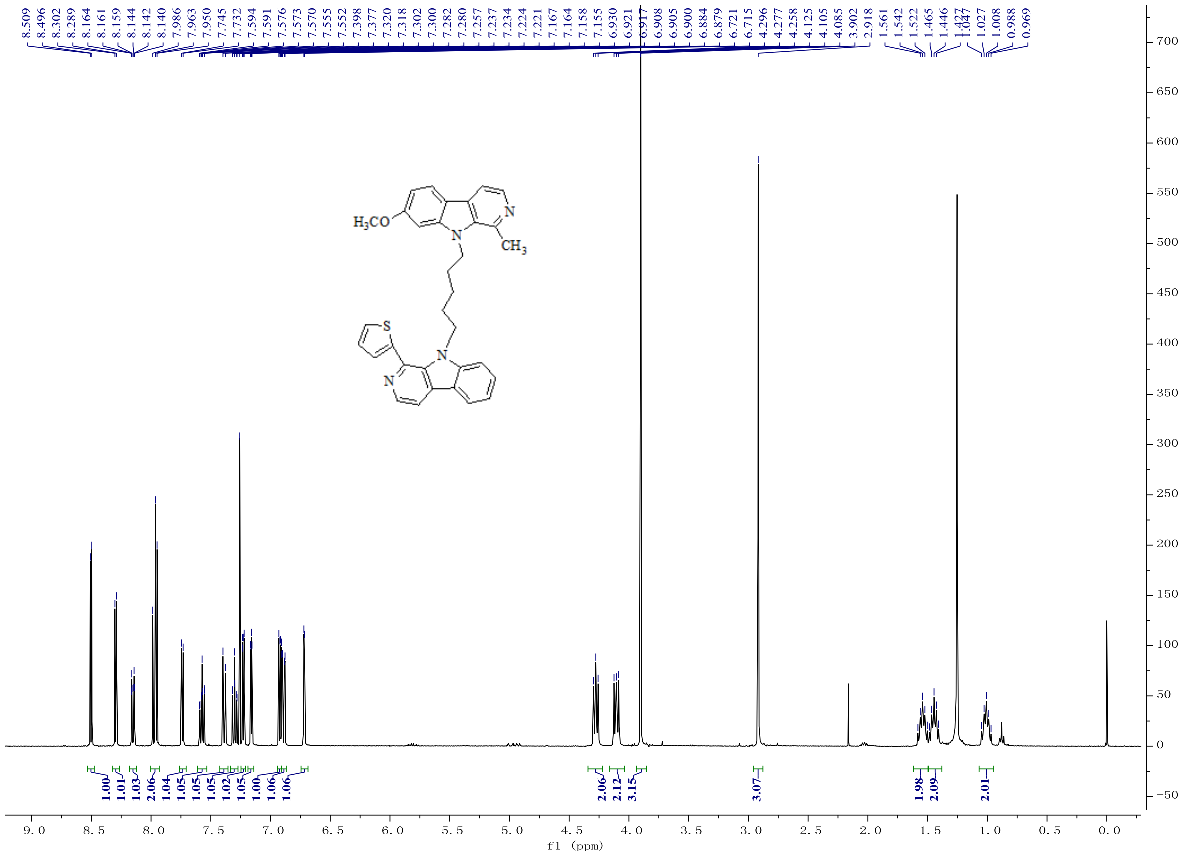

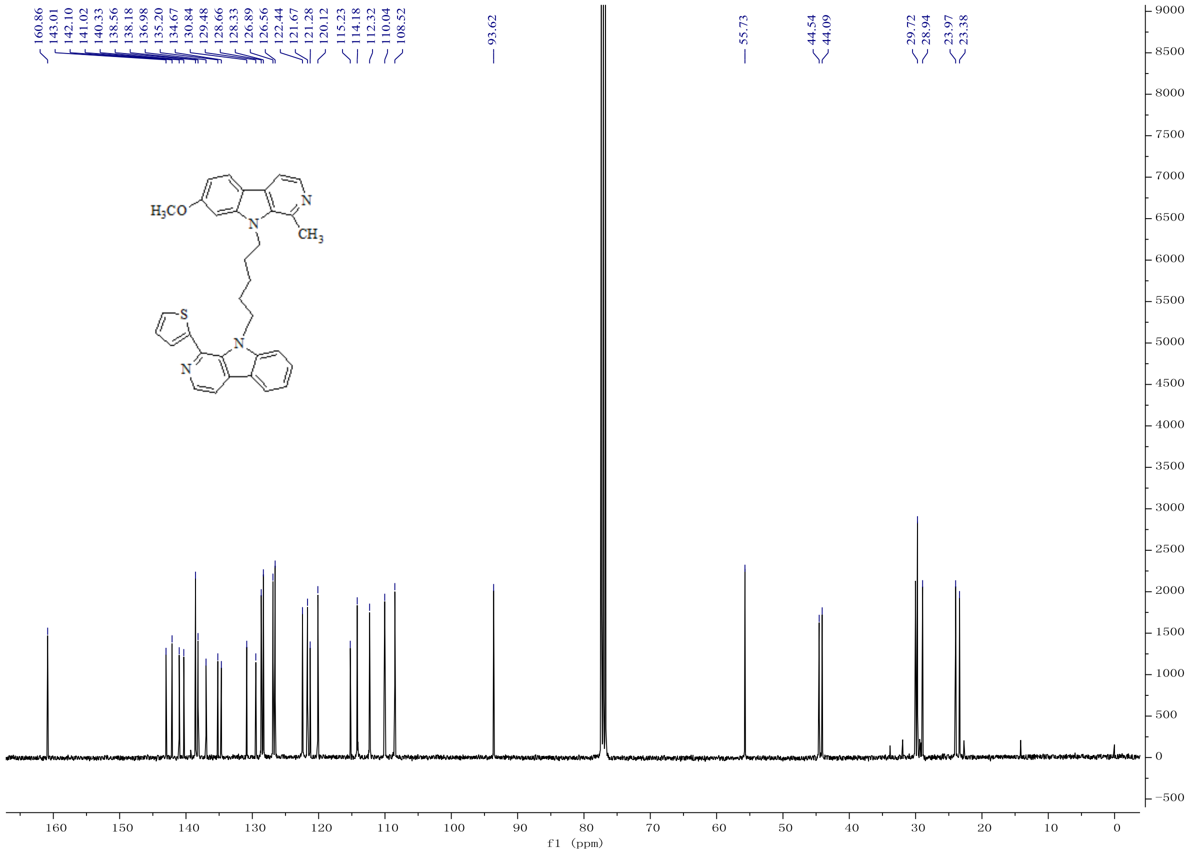

Figure S30 the ^{1}H NMR spectrum and ^{13}C NMR spectrum of compound **5x**
